# A novel variant of social spider optimization using single centroid representation and enhanced mating for data clustering

**DOI:** 10.7717/peerj-cs.201

**Published:** 2019-07-22

**Authors:** Ravichandran Thalamala, Janet Barnabas, A.V. Reddy

**Affiliations:** Department of Computer Applications, National Institute of Technology, Trichy, India

**Keywords:** Social spider optimization, Nature inspired algorithms, Swarm intelligence, Single cluster approach, Data clustering, Cluster centroids

## Abstract

Nature-inspired algorithms are based on the concepts of self-organization and complex biological systems. They have been designed by researchers and scientists to solve complex problems in various environmental situations by observing how naturally occurring phenomena behave. The introduction of nature-inspired algorithms has led to new branches of study such as neural networks, swarm intelligence, evolutionary computation, and artificial immune systems. Particle swarm optimization (PSO), social spider optimization (SSO), and other nature-inspired algorithms have found some success in solving clustering problems but they may converge to local optima due to the lack of balance between exploration and exploitation. In this paper, we propose a novel implementation of SSO, namely social spider optimization for data clustering using single centroid representation and enhanced mating operation (SSODCSC) in order to improve the balance between exploration and exploitation. In SSODCSC, we implemented each spider as a collection of a centroid and the data instances close to it. We allowed non-dominant male spiders to mate with female spiders by converting them into dominant males. We found that SSODCSC produces better values for the sum of intra-cluster distances, the average CPU time per iteration (in seconds), accuracy, the *F*-measure, and the average silhouette coefficient as compared with the *K*-means and other nature-inspired techniques. When the proposed algorithm is compared with other nature-inspired algorithms with respect to Patent corpus datasets, the overall percentage increase in the accuracy is approximately 13%. When it is compared with other nature-inspired algorithms with respect to UCI datasets, the overall percentage increase in the *F*-measure value is approximately 10%. For completeness, the best *K* cluster centroids (the best *K* spiders) returned by SSODCSC were specified. To show the significance of the proposed algorithm, we conducted a one-way ANOVA test on the accuracy values and the *F*-measure values returned by the clustering algorithms.

## Introduction

**Figure 1:**
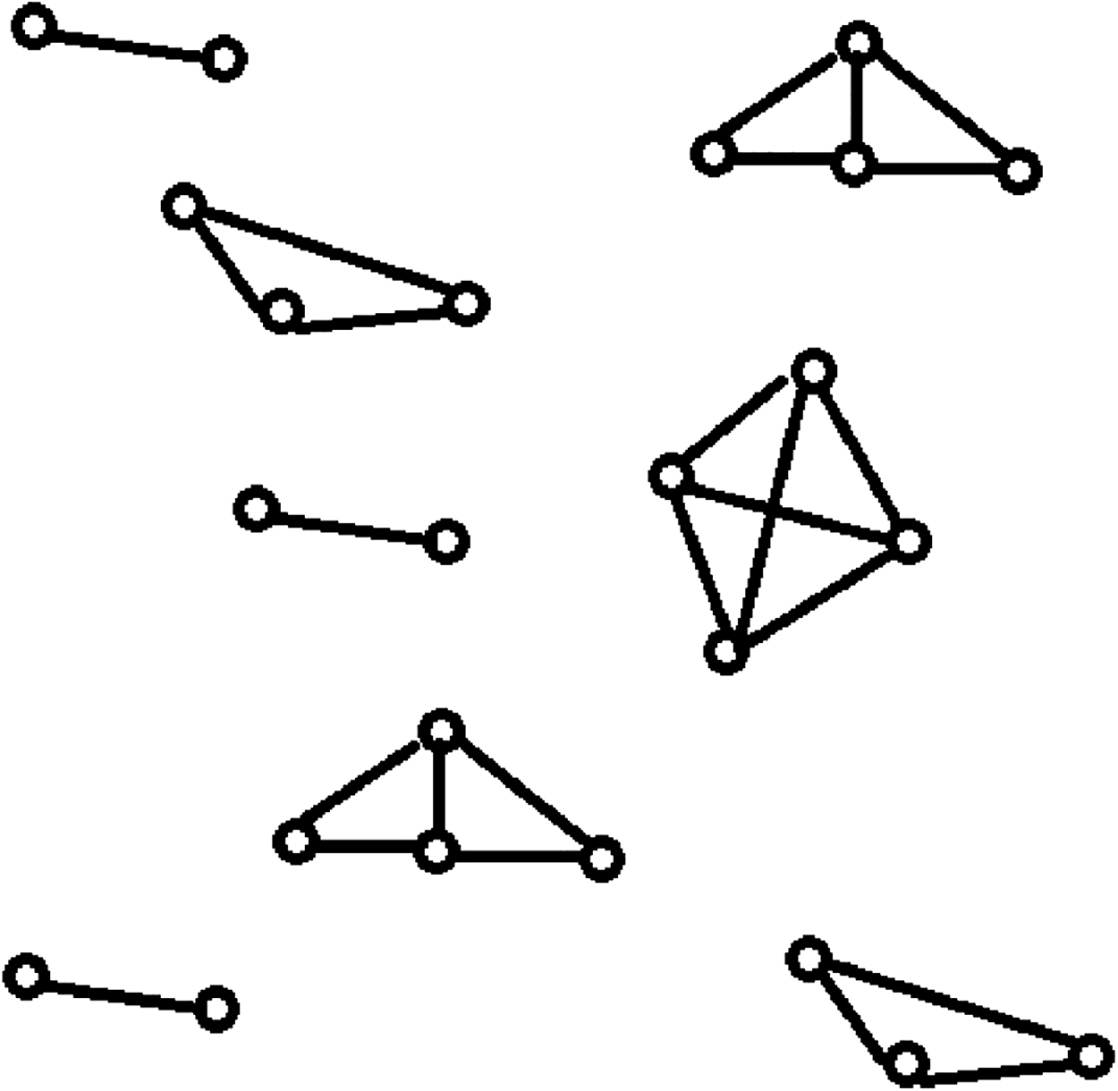
Topological structures present in a computer network: network clustering. It specifies the resultant topological structures of the network clustering when applied on a computer network.

Data clustering is one of the most popular unsupervised classification techniques in data mining. It rearranges the given data instances into groups such that the similar data instances are placed in the same group while the dissimilar data instances are placed in separate groups (Bernábe-Loranca et al., 2014). Data clustering identifies the groups present in a data set, each of which contains related data instances. Network clustering identifies the groups present in a computer network, each of which contains highly connected computers. Network clustering returns the various topological structures present in a computer network as shown in Fig. 1, whereas data clustering returns cluster sets of related data instances. The quality of data clustering is measured using metrics like intra-cluster distances (ICD), inter-cluster distances, *F*-measure, and accuracy. The quality of network clustering is measured using metrics like the global clustering coefficient and the average of the local clustering coefficients.

Data clustering is an NP-hard problem (Aloise et al., 2009) with the objective of minimizing ICD within the clusters and maximizing inter-cluster distances across the clusters (Steinley, 2006). A dataset DS is a collection of data instances. Each data instance in a dataset DS can be represented by a data vector. In a dataset of text files, each text file is a data instance and can be represented as a data vector using mechanisms like term frequency-inverse document frequency (TF-IDF) (Beil, Ester & Xu, 2002). In this paper, we use the terms “data instance” and “data vector” interchangeably and define clustering as a minimization problem that minimizes the sum of intra-cluster distances (SICD). Clustering forms a set of *K* clusters. Let CL be the set of *K* clusters where the SICD is minimized. The mathematical model for the clustering problem can be defined as shown in Eq. (1). The clustering function *F* takes DS and returns CL after minimizing SICD.

(1) $${\rm{Minimize}}\ \,F\left( {{\rm{DS}},{\rm{CL}}} \right) = \sum\limits_{i = 1}^K {\sum\limits_{j = 1}^{{n_i}} {{\rm{distance}}({c_i},{\rm{d}}{{\rm{v}}_{i,j}})} }$$

In Eq. (1), distance is the distance function that returns the distance between two given data vectors, dv_*i*, *j*_ is the *j*th data vector present in *i*th cluster of CL, *n_i_* is the number of data vectors present in *i*th cluster of CL, and *c_i_* is the centroid of *i*th cluster of CL.

The classical clustering algorithms are categorized into hierarchical and partitional algorithms. The main drawback of hierarchical clustering is that the clusters formed in an iteration cannot be undone in the next iterations (Rani & Rohil, 2013). *K*-means is one of the simplest partitional algorithms (Mihai & Mocanu, 2015) but it has two drawbacks: the number of clusters to be formed should be specified apriori, and it generally produces local optima solutions due to its high dependency on initial centroids. Examples of other classical clustering algorithms are BIRCH (Zhang, Ramakrishnan & Livny, 1996), CURE (Guha, Rastogi & Shim, 1998), CLARANS (Ng & Han, 2002), and CHAMELEON (Karypis, Han & Kumar, 1999). Classical algorithms suffer from the drawbacks like the convergence to local optima, sensitivity to initialization, and a higher computational effort to reach global optimum. In order to overcome these problems, nature-inspired meta heuristic algorithms are now used for data clustering.

In this study, we investigated the performance of social spider optimization (SSO) for data clustering using a single centroid representation and enhanced mating operation. The algorithm was experimented on using the Patent corpus5000 datasets and UCI datasets. Each data instance in the UCI dataset is a data vector but the data instances in the Patent corpus5000 datasets are text files. Before we apply the proposed algorithm on these datasets, the text files were represented as data vectors using TF-IDF mechanism. The vector representation of *i*th data instance dv*_i_* present in the dataset DS can be specified using Eq. (2).

(2) $${\rm{d}}{{\rm{v}}_i} = \left( {{w_{i,\,1}},{w_{i,\,2}},{w_{i,\,3}}, \ldots \ldots .,{w_{i,\,t}}} \right)$$

In Eq. (2), *w_i_*_, *j*_ is the term weight of *j*th distinguishable term of the dataset DS in the *i*th data instance, *t* is the total number of distinguishable terms in the dataset DS. The term weight *w_i_*_, *j*_ can be computed using Eq. (3).

(3) $${w_{i,\,j}} = {\rm{T}}{{\rm{F}}_{i,\,j}}*{\rm{ID}}{{\rm{F}}_j}$$

In Eq. (3), TF_*i*, *j*_ (term frequency of the *j*th distinguishable term of the dataset DS in *i*th data instance) is the number of times that the *j*th distinguishable term of the dataset DS occurred in the *i*th data instance, and IDF*_j_* is the inverse document frequency of the *j*th distinguishable term of the dataset DS. IDF*_j_* can be calculated using Eq. (4).

(4) $${\rm{ID}}{{\rm{F}}_j} = \log \left({{m \over n}} \right)$$

In Eq. (4), *n* is the total number of data instances in DS and *m* is the number of data instances in which the *j*th distinguishable term of the dataset DS is present.

### Contributions

In the last decade, nature-inspired algorithms have been successfully applied for solving NP-hard clustering problems. In the state-of-the-art nature inspired algorithms for solving clustering problems, each agent in the population is taken as a collection of *K* clusters. Therefore, the memory requirements and CPU times of these algorithms are very high. These algorithms return the best agents in which SICD is minimized or the average of ICD is minimized. In other words, the fitness of the agent is measured by the consideration that all *K* clusters present in it as a whole. This, however, does not mean that all clusters should have low ICD individually in order to get a low SICD, as even the globally best agent with the best fitness may contain some clusters that have very high ICD.

**Figure 2:**
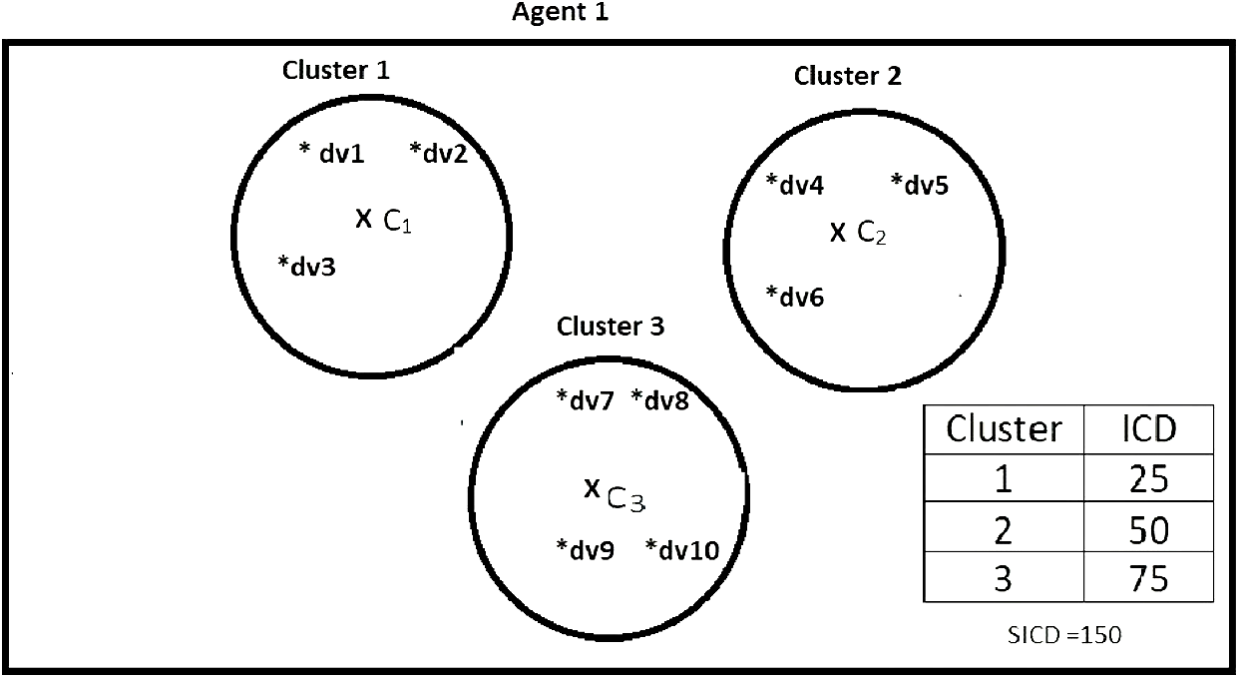
Data instances present in Agent1. It specifies data instances present in Agent1.

**Figure 3:**
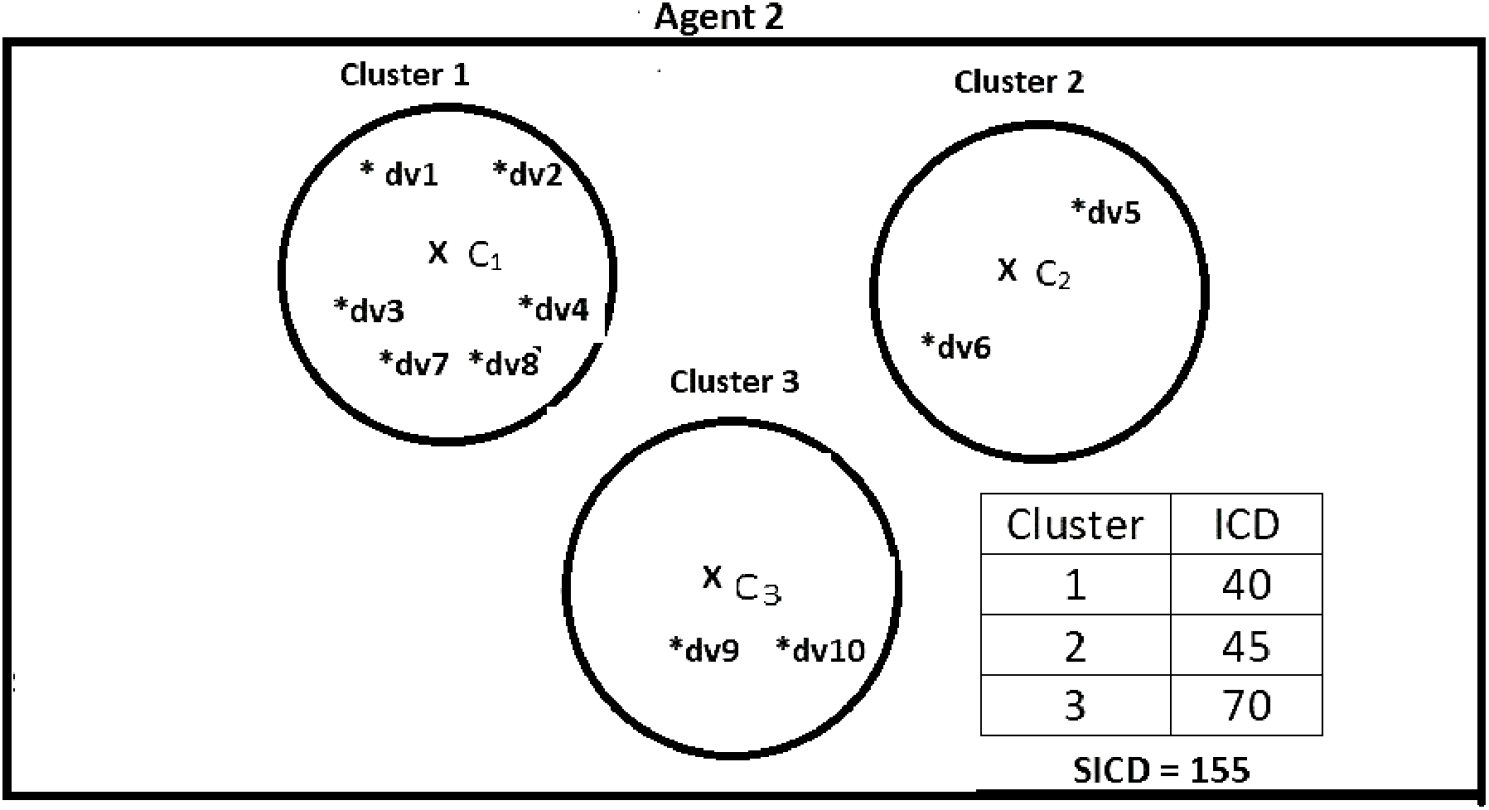
Data instances present in Agent2. It specifies data instances present in Agent2.

**Figure 4:**
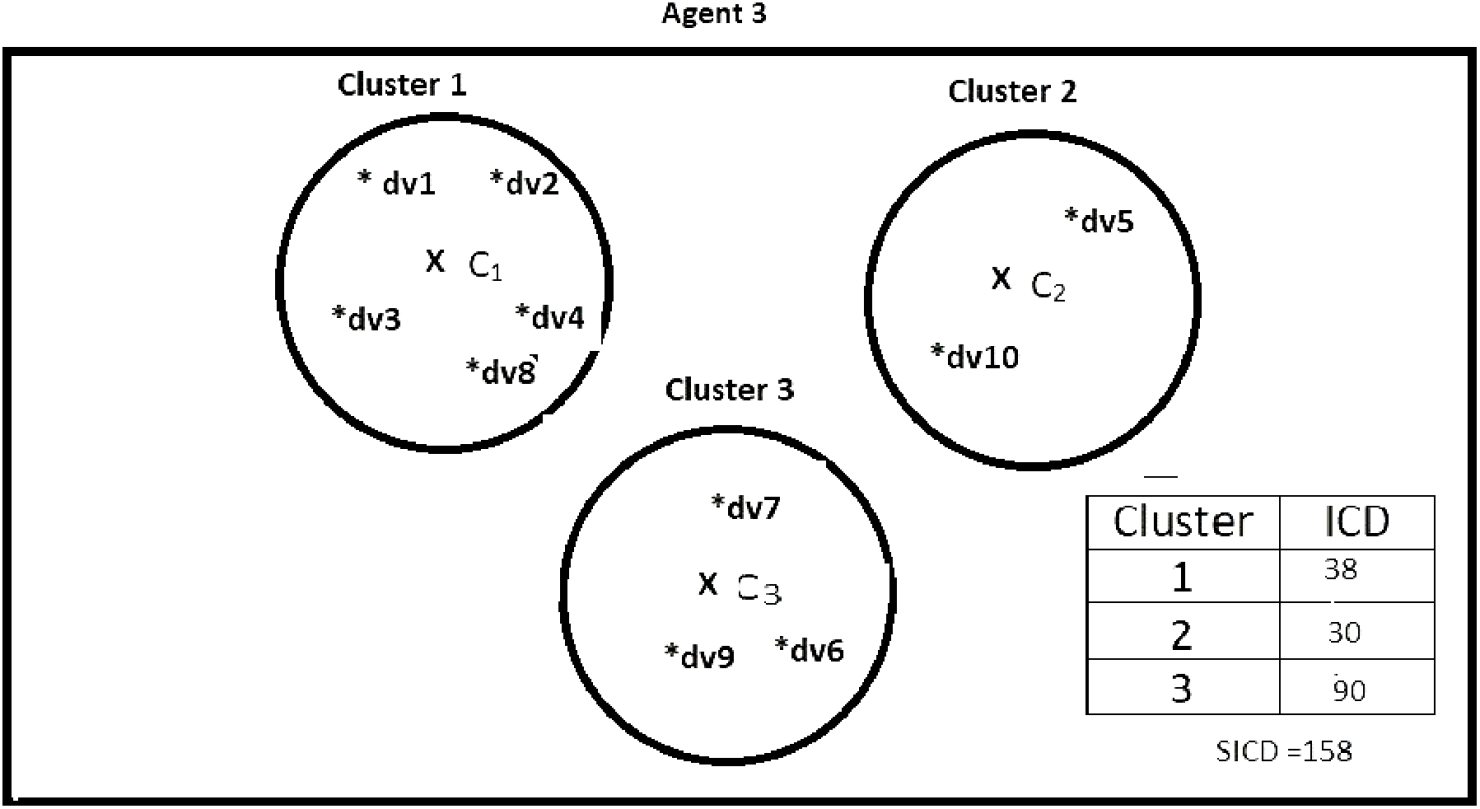
Data instances present in Agent3. It specifies data instances present in Agent3.

**Figure 5:**
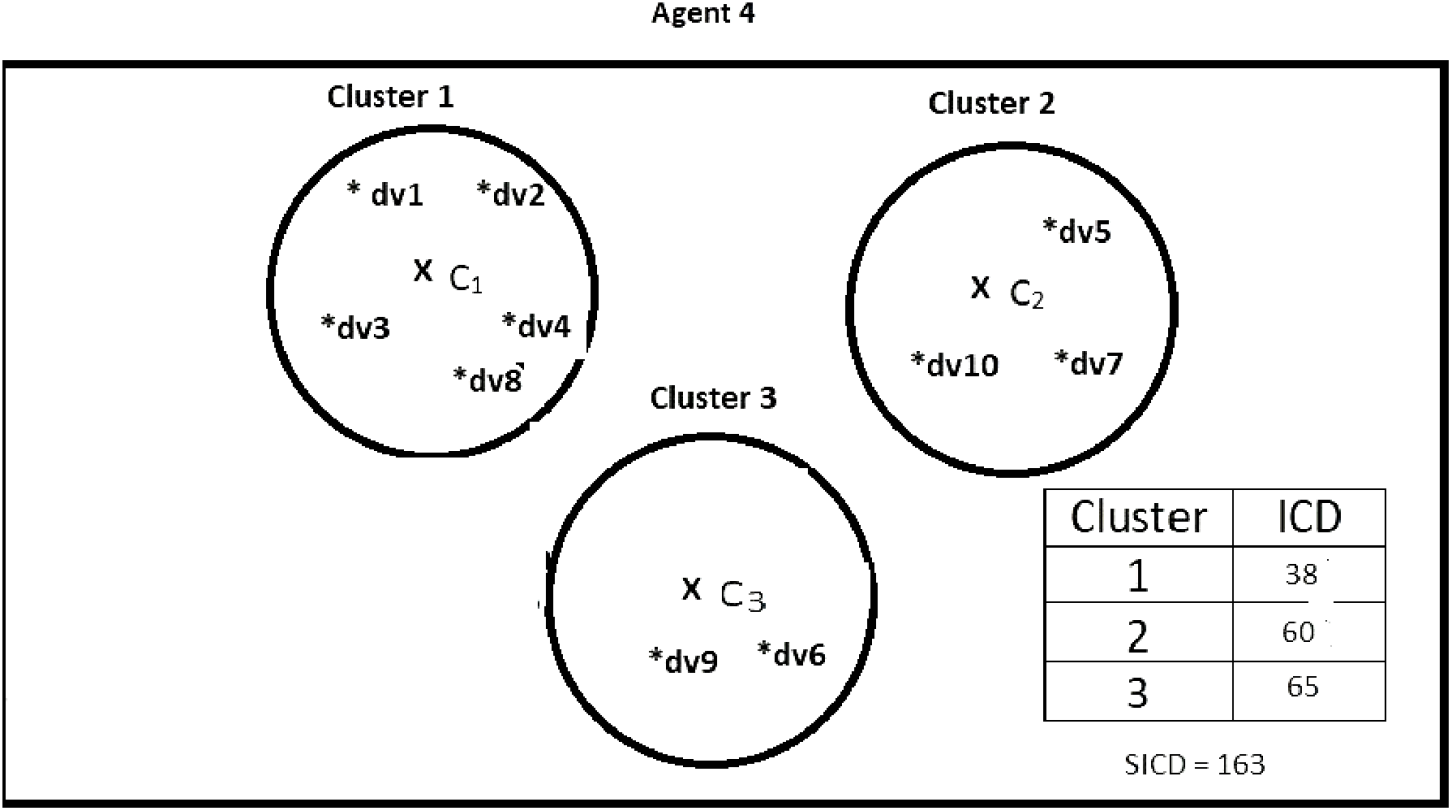
Data instances present in Agent4. It specifies data instances present in Agent4.

Suppose DS = {dv1, dv2, dv3, dv4, dv5, dv6, dv7, dv8, dv9, dv10}, *K* = 3, number of the agents = 4, and the contents of the agents are as shown in Figs. 2–5. According to all state-of-the-art nature-inspired algorithms, the best agent will be *agent1* as it has the lowest SICD value. However, these algorithms will not give any assurance that the three clusters have the lowest individual ICD. Table 1 specifies the best three agents (spiders) returned by our proposed algorithm. The SICD value of the globally best solution is 40 + 30 + 65 = 135, which is less than the SICD value of the globally best solution in the *K*-cluster representation of the agent. Therefore, the clustering results produced by the start-of-the art algorithms that use *K*-centroid representation for agents may not be highly accurate. The proposed approach not only focuses on SICD but also on the individual ICD of the clusters.

**Table 1:** The best three agents returned by SSODCSC.

Agent	Data vectors	Intra-cluster distance	Part of best solution?
Spider 1	dv1, dv2, dv3	25	No
Spider 2	dv4, dv5, dv6	50	No
Spider 3	dv7, dv8, dv9, dv10	75	No
Spider 4	dv1, dv2, dv3, dv4, dv7, dv8	40	Yes
Spider 5	dv5, dv6	45	No
Spider 6	dv9, dv10	70	No
Spider 7	dv1, dv2, dv3, dv4, dv8	38	No
Spider 8	dv5, dv10	30	Yes
Spider 9	dv6, dv7, dv9	90	No
Spider 10	dv1, dv2, dv3, dv4, dv8	38	No
Spider 11	dv5, dv7, dv10	60	No
Spider 12	dv6, dv9	65	Yes

In our proposed algorithm, social spider optimization for data clustering using single centroid (SSODCSC), each spider is represented by a single centroid and the list of data instances close to it. This representation requires *K* times less memory requirements than the representation used by the other state-of-the-art nature-inspired algorithms like SSO, as shown below. Each data instance in the dataset is given an identification number. Instead of storing data instances, we stored their identification numbers (which are integer values) in spiders.

For SSODCSC:
Number of spiders used = 50
Number of iterations for best clustering results = 300
Total number of spiders to be computed = 300 * 50 = 15,000
Memory required for storing a double value = 8 bytes
Memory required for storing a spider’s centroid (that consists of m dimension values) = 8 * m bytes, where m is the number of dimensions present in the dataset.
Memory required for storing an integer value representing identification number of a data instance = 4 bytes.
Maximum memory required for storing the list of identification numbers of data instances closer to the centroid = 4 * *n* bytes, where *n* is number of data instances present in the dataset.
Maximum memory required for a spider = 8 * *m* + 4 * *n* bytes
Therefore, total computational memory of SSODCSC = 15,000 * (8 * *m* + 4 * *n*) bytes.

For SSO:
Number of spiders used = 50
Number of iterations for best clustering results = 300
Total number of spiders to be computed = 300 * 50 = 15,000
Memory required for storing a double value = 8 bytes
Memory required for storing *K* centroids of a spider = 8 * *m* * *K* bytes, where *m* is the number of dimensions present in the dataset.
Memory required for storing an integer value representing identification number of a data instance = 4 bytes
Maximum memory required for storing *K* lists of identification numbers of data instances (where each list is associated with a centroid) = 4 * *n* * *K* bytes, where n is the number of data instances present in the dataset, and *K* is the number of centroids present in each spider.
Maximum memory required for a spider = K*(8 * *m* + 4 * *n*) bytes
Therefore, total computational memory of SSO = 15,000 * K* (8 * *m* +4 * *n*) bytes.

The time required for initiating the spiders will be less in this representation. The average CPU time per iteration depends on the time required for computing fitness values and the time required for computing the next positions of the spiders in the solution space. The fitness values and next positions of spiders can be computed in less time with single centroid representation, so that the average CPU time per iteration reduces gradually. The proposed algorithm returns best *K* spiders such that the union of the lists of data instances present in them will produce exactly all of the data instances in DS.

In the basic SSO algorithm, non-dominant males are not allowed in the mating operation because of their low weight values. They do not receive any vibrations from other spiders and have no communication in the web, as the communication is established through vibration only (Shukla & Nanda, 2016). Therefore, their presence in the solution space is questionable. Moreover, their next positions are dependent on the existing positions of the dominant male spiders (Cuevas et al., 2013). They cannot be part of the selected solution when dominant male spiders are in the solution space. In our proposed algorithm, SSODCSC, we convert them into dominant male spiders by increasing their weight values and then allowing them to participate in the mating operation to produce a new spider, better than the worst spider in the population. In SSO, each dominant male mates with a set of females and produces a new spider. The weight of the new spider may or may not be greater than that of the worst spider. But as we make the weight of each non-dominant male spider greater than the average weight of the dominant male spiders in SSODCSC, the new spider produced by the non-dominant male spider is surely better than the worst spider. In other words, not only did we convert non-dominant male spiders into dominant male spiders, but also we made them more effective than the dominant male spiders. Therefore, each spider receives vibrations from other spiders and has a chance at becoming a part of the selected solution, unlike in SSO. At each iteration of SSODCSC, the population size is the same but the spiders with greater weight values are introduced in place of the worst spiders. As a result, the current solution given by SSODCSC moves toward the globally best solution as the number of iterations is increased. We applied SSODCSC on feature-based datasets and text datasets.

This paper is organized as follows: “Related Work” describes the recent related work on solving clustering problems using nature-inspired algorithms, “Proposed Algorithm: SSODCSC” describes SSODCSC, “Results” includes experimental results, and we conclude the paper with future work in the section “Discussion.”

## Related Work

Shukla & Nanda (2016) proposed two clustering algorithms based on the original version of SSO and a parallel version of SSO (P-SSO). P-SSO computes the next position of female spiders, dominant male spiders, and non-dominant male spiders simultaneously in each iteration. They applied the two algorithms on low dimensional datasets, high dimensional datasets, overlapping datasets, and non-overlapping datasets, and found that the two algorithms are able to produce consistent clustering results as compared with other clustering algorithms. They designed a flood image segmentation application based on their proposed work and got two times better accuracy than the *K*-means when applied on NASA satellite images of flood affected areas of Chennai.

Zhou et al. (2018), used the symbiotic organism search (SOS) algorithm for solving clustering problems. The SOS algorithm mimics the interactive behavior of the organisms in nature. In SOS, new solutions are generated by imitating the biological interactions between two organisms in the ecosystem. SOS implements three phases, namely, the mutualism phase, the commensalism phase, and the parasitism phase. In the mutualism phase, the organisms interact to increase their mutual survival advantage. In the commensalism phase, the interaction benefits one organism but does not impact the other. In the parasitism phase, the organism with better fitness will kill the other. The proposed algorithm produced better clustering results when compared with other algorithms with low dimensional datasets. However, the authors did not apply SOS on high dimensional datasets. The algorithm suffers from an imbalance between exploration and exploitation due to its high randomness.

Nayak et al. (2018), hybridized the Elicit Teaching learning based optimization approach with the fuzzy c-means (FCM) clustering algorithm. At each iteration of Elicit Teaching learning-based optimization, the worst entities are replaced with the best entities in each cluster group. The best cluster centroids produced by Elicit Teaching learning based optimization are taken as the inputs for the FCM clustering algorithm. They found that the proposed algorithm produces clustering solutions of better fitness values when compared with other clustering algorithms.

Zhou et al. (2017), proposed a simplex method based social spider optimization (SMSSO) algorithm to overcome the drawbacks of SSO, namely local optima entrapment and poor convergence rates. In the proposed algorithm, the spider with the worst fitness is replaced by a reflected or extended alternate spider so that the global search may be improved. The largest dataset used in the experiments has only 13 dimensions. The proposed algorithm looks good with low dimensional datasets. It may not be as effective for high dimensional datasets as the simplex mechanism will become expensive for those datasets. The differences between SMSSO and our proposed algorithm SSODCSC are as follows: in SMSSO, the initial solution moves along the edges of the polytope until it reaches the optimal solution, in SSODCSC, the solution space will have spiders of better fitness values after every iteration. SMSSO supports the mating of dominant males only, but SSODCSC allows the mating of both dominant and non-dominant male spiders with female spiders. In SMSSO, the fitness of the new spider may or may not be better than that of the worst spider. In SSODCSC, the fitness of the new spider is always greater than that of the worst spider. In SMSSO, each spider is represented as a collection of *K*-centroids. In SSODCSC, each spider is represented as a single centroid. SMSSO returns the best spider, whereas SSODCSC returns a set of first *K* best spiders.

Han et al. (2017), proposed the bird flock gravitational search algorithm (BFGSA) to enable the gravitational search algorithm escape from sub optimal solutions. The authors used a concept called collective response of object reorientation to avoid stagnation. In that model, if the fitness of the global optimum remains the same in several subsequent iterations, the proposed algorithm defines the collective response of the object reorientation that updates the position of each object using the mean position of its nearest seven neighbors. The simulation results indicate that BFGSA can be used for both low and high dimensional datasets. The proposed algorithm convergences occur in 500 iterations, which is relatively high when compared with other algorithms.

Jothi, Mohanty & Ojha (2018), proposed minimum spanning tree (MST) based clustering on the partition-based nearest neighbor graph for reducing the computational overhead. The proposed algorithm produces a sparse local neighborhood graph (LNG) and then the approximate MST is constructed from LNG. They showed that the proposed algorithm outperforms the traditional algorithms by reducing both the size and computational time to construct the LNG. Experiments are conducted on both synthetic and real datasets.

Chen et al. (2018), proposed a novel optimum-path forest (OPF) clustering algorithm that can be used for remote sensing segmentation. They defined a probability density function using the principle that the cluster centers depend on their distances from samples with higher densities. They applied the proposed algorithm on five remote sensing land cover images. The clustering results show that the proposed algorithm outperforms the original OPF approach.

Nayak et al. (2014), proposed an improved firefly-based fuzzy c-means algorithm (improved FAFCM) to resolve the drawbacks of the FCM algorithm (FCM) using firefly algorithm. They used the firefly algorithm to minimize the objective function value of the FCM algorithm. The output centroids of the firefly algorithm are passed to the FCM algorithm as initial centroids so that it refines them further. They found that an improved FAFCM produces better clustering results as compared to FCM, particle swarm optimization (PSO), and FAFCM.

De Andrade Silva, Hruschka & Gama (2017), proposed a fast evolutionary algorithm for clustering data streams (FEAC-Stream). It is capable of estimating the number of clusters to be formed from data in an online fashion. The Page–Hinkley test is used by FEAC-Stream to identify eventual degradation in the induced cluster quality. The proposed algorithm is based on the assumption that clusters provide useful information about the dynamics of the data stream. They applied the proposed algorithm on synthetic and real-world data streams and showed that the proposed algorithm produces better clustering results than other algorithms.

Costa et al. (2015), proposed a nature-inspired approach for data clustering based on the optimum-path forest algorithm (OPFC). OPFC accepts a graph representation of the dataset.

The nodes of the graph are samples and each sample is connected to its *k*-nearest neighbors. Each node has a weight. The weight is its probability density function value, which is computed using its distances from *l k*-nearest neighbors. After the *k*-*nn* graph is constructed, OPFC finds roots which are the nodes with the maximum of probability density function values and propagates one optimum-path cluster from each root to the remaining nodes in the graph.

Alswaitti, Albughdadi & Isa (2018), presented a novel approach for data clustering based on particle swarms. For balancing exploitation and exploration, they used the kernel density estimation technique and estimated multidimensional gravitational learning coefficients. The kernel density estimation technique is used for avoiding premature convergence. They showed that the proposed algorithm produces better accuracy and better cluster compactness than other clustering algorithms when applied on benchmark datasets from the UCI Machine Learning Repository.

## Proposed Algorithm: SSODCSC

**Figure 6:**
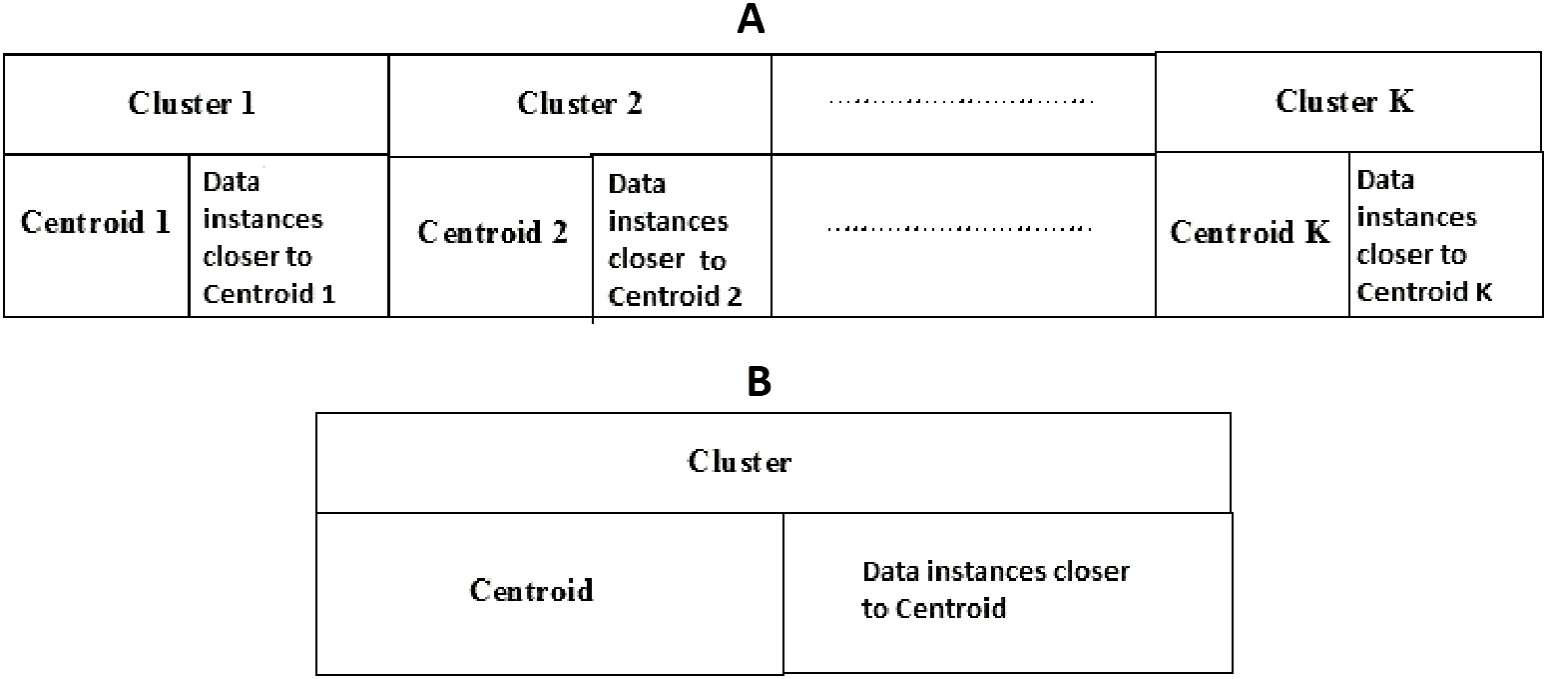
(A) *K*-cluster representation of a spider; (B) single cluster representation of a spider. The figure specifies the components of a spider in *K*-cluster and single cluster representations.

**Figure 7:**
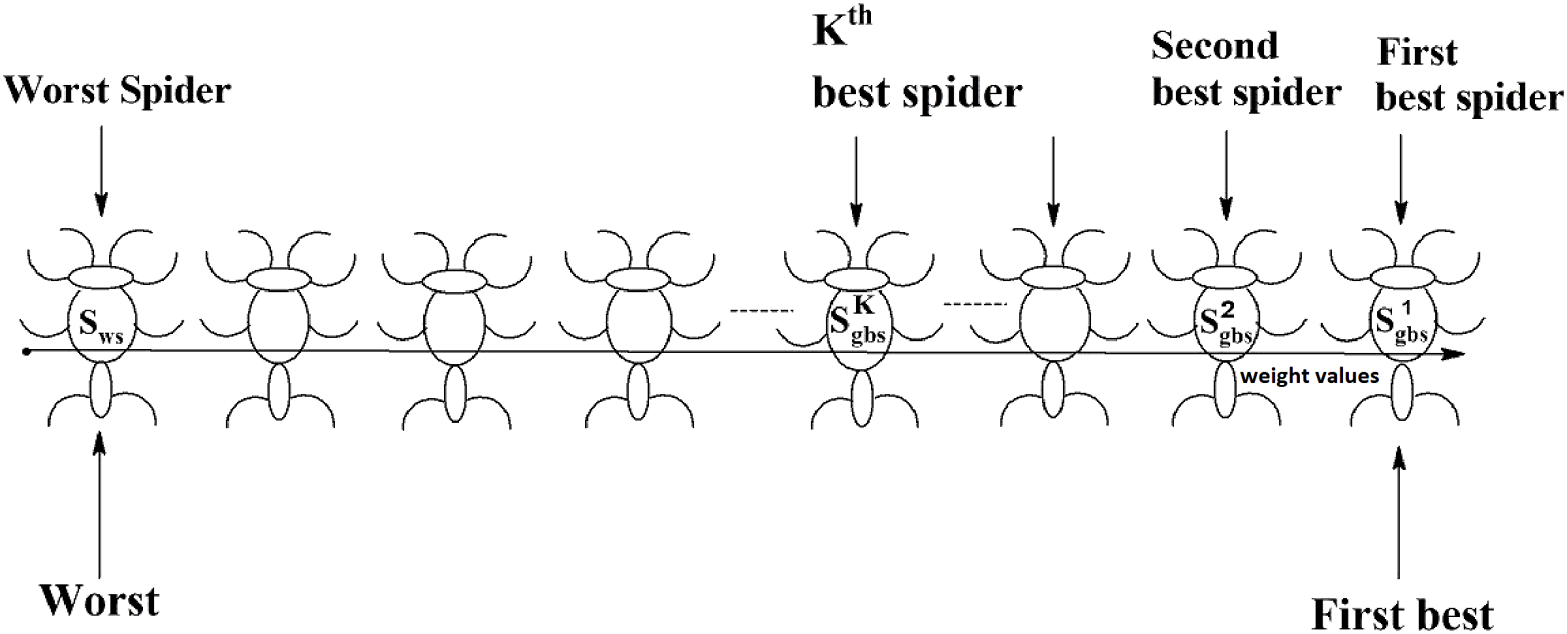
Spiders on the scale of weight values.

**Figure 8:**
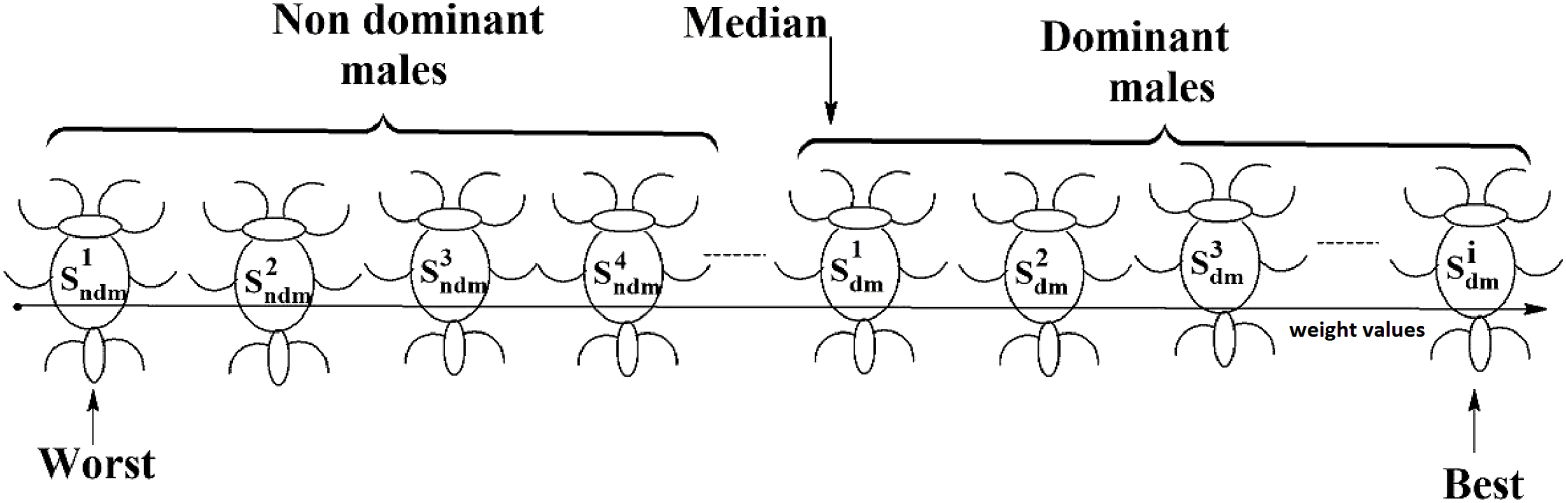
Male spiders on the scale of weight values.

Social spider optimization is based on the cooperative behavior of social spiders for obtaining a common food. They are classified into two types, namely male spiders and female spiders (Cuevas et al., 2013). Nearly 70% of the population is female. Each spider is characterized by its position, fitness, weight, and vibrations received from other spiders (Thalamala, Reddy & Janet, 2018). In *K*-centroid representation, each spider has *K*-centroids which are associated with a list of data instances closer to it as shown in Fig. 6A. In SSODCSC, each spider has two components, namely a centroid and a list of identification numbers of data instances closer to it, as shown in Fig. 6B. The number of data instances close to the centroids of spiders may be different, the length of each spider may be different; however, the length of the centroid component of each spider is fixed. Therefore, we used only centroid components of the spiders to specify their position in the solution space. When the spiders move in the solution space, only the centroid components are moved or updated. The new list of identification numbers of data instances may be found to be closer to the centroid, depending on its new location. We use the terms spider, position of spider, and centroid component of spider interchangeably in this article. The fitness of a spider is the sum of the distances of its data instances from its centroid. The weights of the spiders are computed based on their fitness values. In SSODCSC, the weight of a spider is inversely proportional to the fitness value of the spider. A spider with the first largest weight (first smallest fitness) is known as the globally best spider *s^1^_gbs_*. In this paper, we use the notations *s^1^_gbs_* and *s_gbs_* interchangeably. In SSODCSC, *s^1^_gbs_* will have the largest weight and lowest value for the sum of the distances of data instances from the centroid. The spider with the least weight (largest fitness value) is known as worst spider *s_ws_,* as shown in Fig. 7. In SSODCSC, *s_ws_* will have the least weight and largest value for the sum of the distances of the data instances from the centroid. Each spider receives vibrations from the globally best spider *s_gbs_*, the nearest better spider *s_nbs_*, and the nearest female spider *s_nfs_*. The male spiders are classified into two types, namely dominant males and non-dominant males. The weight of a dominant male spider is greater than or equal to the median weight of male spiders (Cuevas & Cienfuegos, 2014) as shown in Fig. 8. The male spiders that are not dominant males are called non-dominant males. A female spider can either attract or repulse other spiders. The weight of a spider *s* can be computed using Eq. (5). The lower the sum of the distances (i.e., fitness), the higher the weight of the spider will be in SSODCSC.

(5) $${\rm{weight}}\,{\rm{[}}s{\rm{]}} = {{{\rm{fitness}}\left(s \right)-{\rm{fitness}}\left({{s_{ws}}} \right)} \over {{\rm{fitness}}\left({{s_{gbs}}} \right)-{\rm{fitness}}\left({{s_{ws}}} \right)}}$$

The SSODCSC algorithm returns spiders *s^1^_gbs_*, *s^2^_gbs_* ... and *s^K^_gbs_* that have the first *K* largest weight values (first *K* smallest fitness values) such that the union of the data instances present in them will give exactly all of the data instances present in the dataset. We used a two-dimensional array, namely *spider*, to store the centroid components of all spiders. For example, in the case of the Iris dataset, the centroid component of first spider is stored in spider [1, 1], spider [1, 2], spider [1, 3], and spider [1, 4], as Iris has four dimensions. The number of spiders returned by SSODCSC depends on the number of clusters inherently present in the dataset. For example, in the case of the Iris dataset, though we use 50 spiders, the algorithm returns the first best, second best, and third best spiders only, because the Iris dataset inherently has three clusters. In the following subsections, we explain how the spiders are initialized in the solution space, the data instances are assigned to them, the next positions of the spiders are found, and the mating operation produces a new spider.

### Initialization

Social spider optimization for data clustering using single centroid starts with the initialization of spiders in the solution space. Initially all spiders are empty. The fitness of each spider is set to 0, and the weight is set to 1. Each spider s is initialized with a random centroid using Eq. (6).

(6) $${\rm{spider}}\,[s,d] = {\rm{lowerbound}}\,(d) + {\rm{random}}\,(0,1)*({\rm{upperbound}}\,(d) - {\rm{lowerbound}}\,(d))$$

where spider [*s*, *d*] is *d*th dimension of the centroid of spider *s*, lowerbound (*d*) and upperbound (*d*) are the smallest and largest values of the *d*th dimension of the dataset, respectively.

### Assignment of data instances

The distances of each data instance from the centroids of all spiders are calculated using the Euclidean distance function. A data instance is assigned to the spider that contains its nearest centroid.

### Next positions of spiders

The spiders are moved across the solution space in each iteration of SSODCLC based on their gender. The movement of a spider in the solution space depends on the vibrations received from other spiders. The intensity of the vibrations originated from spider *s_j_* to spider *s_i_* can be found using Eq. (7) and depends on the distance between the two spiders and the weight of spider *s_j_*.

(7) $${\rm{vibrations}}\left[ {{s_i},{s_{j}}} \right] = {\rm{weight}}\left[ {{s_j}} \right]*{e^{ - {\rm{distance}}{{\left( {{s_i},{s_j}} \right)}^2}}}$$

#### Next positions of female spiders

**Figure 9:**
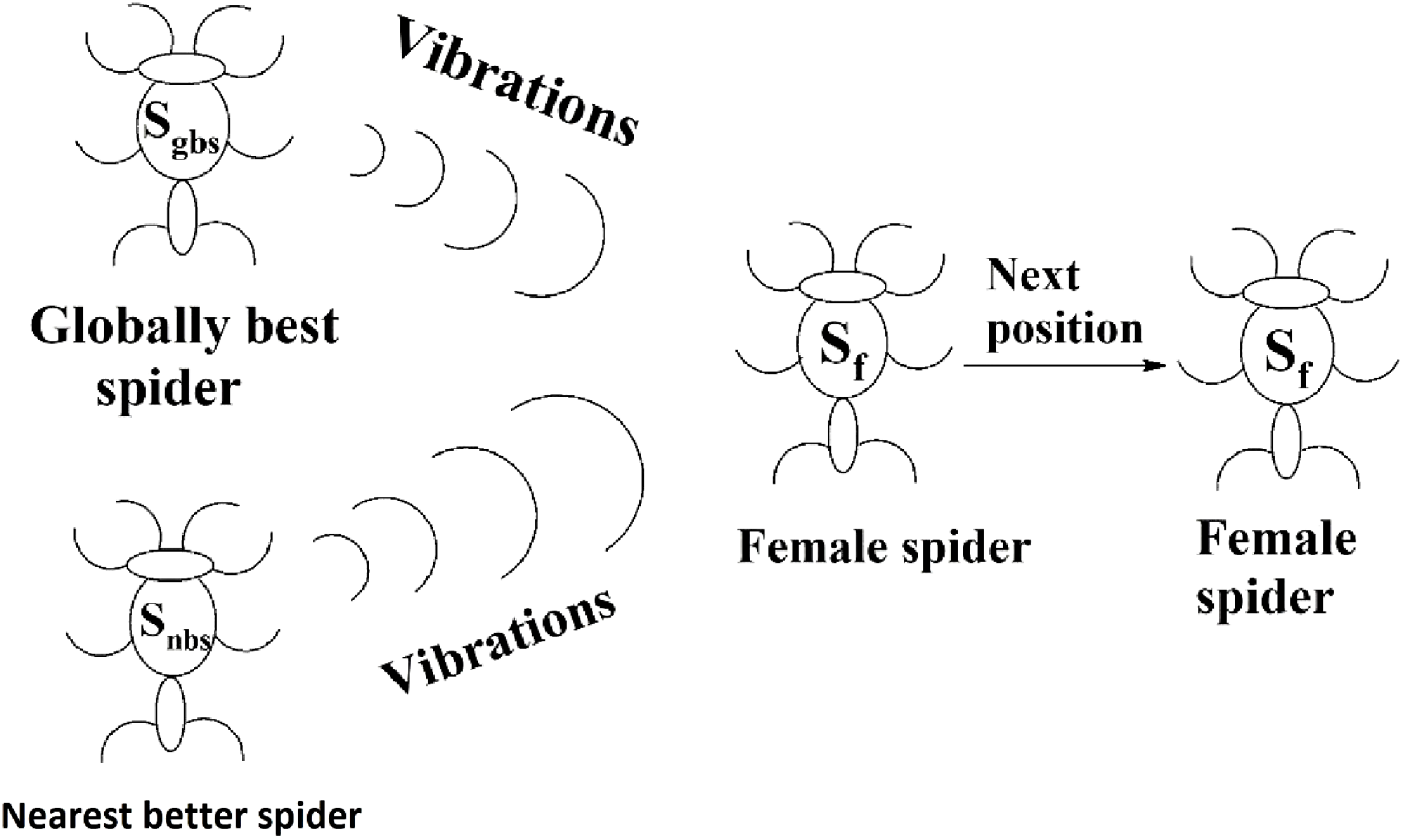
Next position of a female spider in SSODCSC. The figure specifies how the next position of a female spider is calculated in SSODCSC.

The movement of a female spider *s_f_* depends on the vibrations from the globally best spider *s_gbs_* and its nearest better spider *s_nbs_* as shown in Fig. 9. To generate the next position of a female spider *s_f_*, a random number is generated and if it is less than the threshold probability (TP), the female spider attracts other spiders and the position of it is calculated according to Eq. (8). If not, it repulses other spiders and the position of it calculated according to Eq. (9). In Eqs. (8) and (9), α, β, γ, and δ are random numbers from the interval [0, 1].

(8) $$\eqalign{
 & {\rm{spider}}{\mkern 1mu} [{s_f},d] = {\rm{spider}}{\mkern 1mu} [{s_f},d] + \alpha *\left( {{\rm{spider}}{\mkern 1mu} [{s_f},d] - {\rm{spider}}{\mkern 1mu} [{s_{gbs}},d]} \right)*{\rm{weight}}{\mkern 1mu} [{s_{gbs}}] \cr 
 & \quad \quad \quad \quad \quad \quad *{{\rm{e}}^{ - {\rm{distance}}{{({s_f},{s_{gbs}})}^2}}} + \beta *\left( {{\rm{spider}}{\mkern 1mu} [{s_f},d] - {\rm{spider}}{\mkern 1mu} [{s_{nbs}},d]} \right) \cr 
 & \quad \quad \quad \quad \quad \quad *{\rm{weight}}{\mkern 1mu} [{s_{nbs}}]*{{\rm{e}}^{ - {\rm{distance}}{{({s_f},{s_{nbs}})}^2}}} + \gamma *(\delta - 0.5) \cr} $$

(9) $$\eqalign{
 & {\rm{spider}}{\mkern 1mu} [{s_f},d] = {\rm{spider}}{\mkern 1mu} [{s_f},d] + \alpha *\left( {{\rm{spider}}{\mkern 1mu} [{s_f},d] - {\rm{spider}}{\mkern 1mu} [{s_{gbs}},d]} \right) \cr 
 & \quad \quad \quad \quad \quad \quad *{\rm{weight}}{\mkern 1mu} [{s_{gbs}}]*{e^{ - {\rm{distance}}{{({s_f},{s_{gbs}})}^2}}} - \beta *\left( {{\rm{spider}}{\mkern 1mu} [{s_f},d] - {\rm{spider}}{\mkern 1mu} [{s_{nbs}},d]} \right) \cr 
 & \quad \quad \quad \quad \quad \quad *{\rm{weight}}{\mkern 1mu} [{s_{nbs}}]*{e^{ - {\rm{distance}}{{({s_f},{s_{nbs}})}^2}}} + \gamma *(\delta - 0.5) \cr} $$

#### Next position of male spiders

The solution space consists of female spiders and male spiders. When data instances are added or removed from them, their fitness values and weight values will change. If the current weight of a male spider is greater than or equal to the median weight of dominant male spiders, it will be considered to be a dominant male spider. The male spiders that are not dominant male spiders are called non-dominant male spiders. The next position of a dominant male *s_dm_* can be calculated using Eq. (10).

(10) $$\eqalign{
 & {\rm{spider}}\,[{s_{dm}},\,d] = {\rm{spider}}\,[{s_{dm}},\,d] + \alpha *\left( {{\rm{spider}}\,[{s_{dm}},\,d] - {\rm{spider}}\,[{s_{nfs}},\,d]} \right) \cr 
 & \quad \quad \quad \quad \quad \quad \quad *{\rm{weight}}\,[{s_{nfs}}]*{{\rm{e}}^{ - {\rm{distance}}{{({s_{dm}},\,{s_{nfs}})}^2}}} + \gamma *(\delta - 0.5) \cr} $$

**Figure 10:**
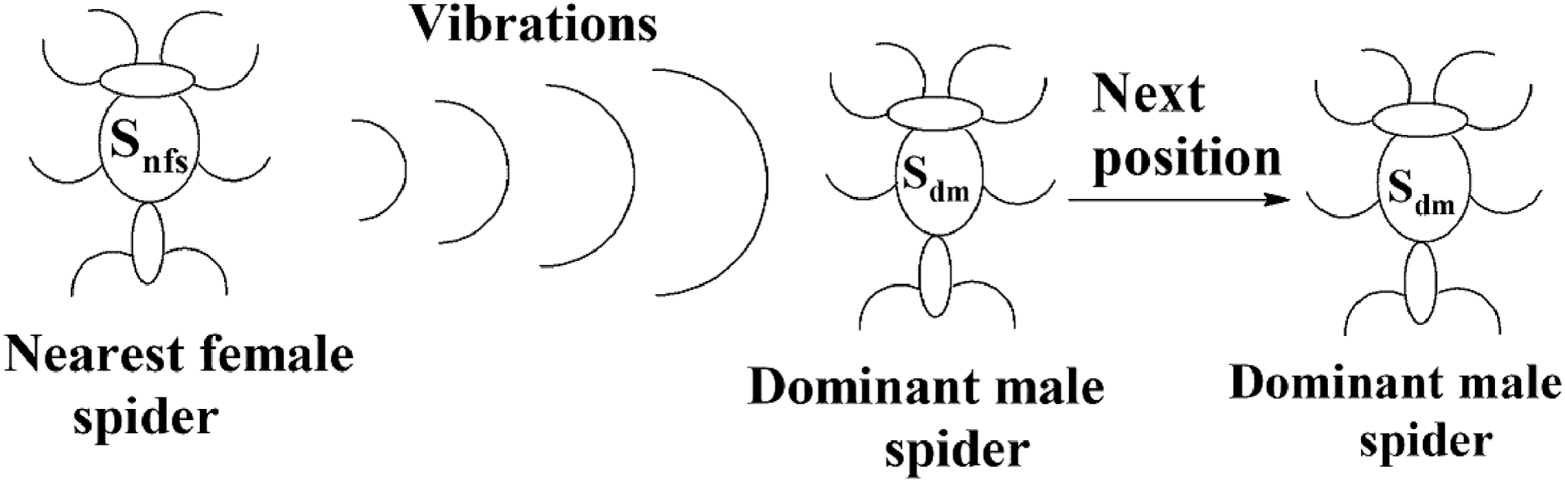
Next position of a dominant male spider in SSODCSC. The figure specifies how the next position of a dominant male spider is calculated in SSODCSC.

The position of the spider depended only on the vibrations received from its nearest female spider *s_nfs_*. The pictorial representation of this is specified in Fig. 10. The weighted mean of the male population, *W,* can be obtained using Eq. (11). Let *N_f_* be the total number of female spiders in the spider colony and *N_m_* be the total number of male spiders. Then the female spiders can be named as ${s_{{f_1}}},{s_{{f_2}}},{s_{{f_3}}}, \ldots \ldots ..,{s_{{f_{{N_f}}}}}$ and the male spiders can be named as ${s_{{m_1}}},{s_{{m_2}}},{s_{{m_3}}}, \ldots \ldots ..,{s_{{m_{Nm}}}}$.

(11) $$W = {{\sum\nolimits_{i = 1}^{{N_m}} {{\rm{spider}}\ \left[{{s_{{m_i}}},d} \right]} *{\rm{weight}}\ \left( {{s_{{m_i}}}} \right)} \over {\sum\nolimits_{i = 1}^{{N_m}} {*{\rm{weight}}\ \left( {{s_{{m_i}}}} \right)} }}$$

The next position of the non-dominant male spider *s_ndm_* can be calculated using Eq. (12) and depends on the weighted mean of the male population.

(12) $${\rm{spider}}\,[{s_{ndm}},d] = {\rm{spider}}\,[{s_{ndm}},d] + \alpha *W$$

### Mating operation

**Figure 11:**
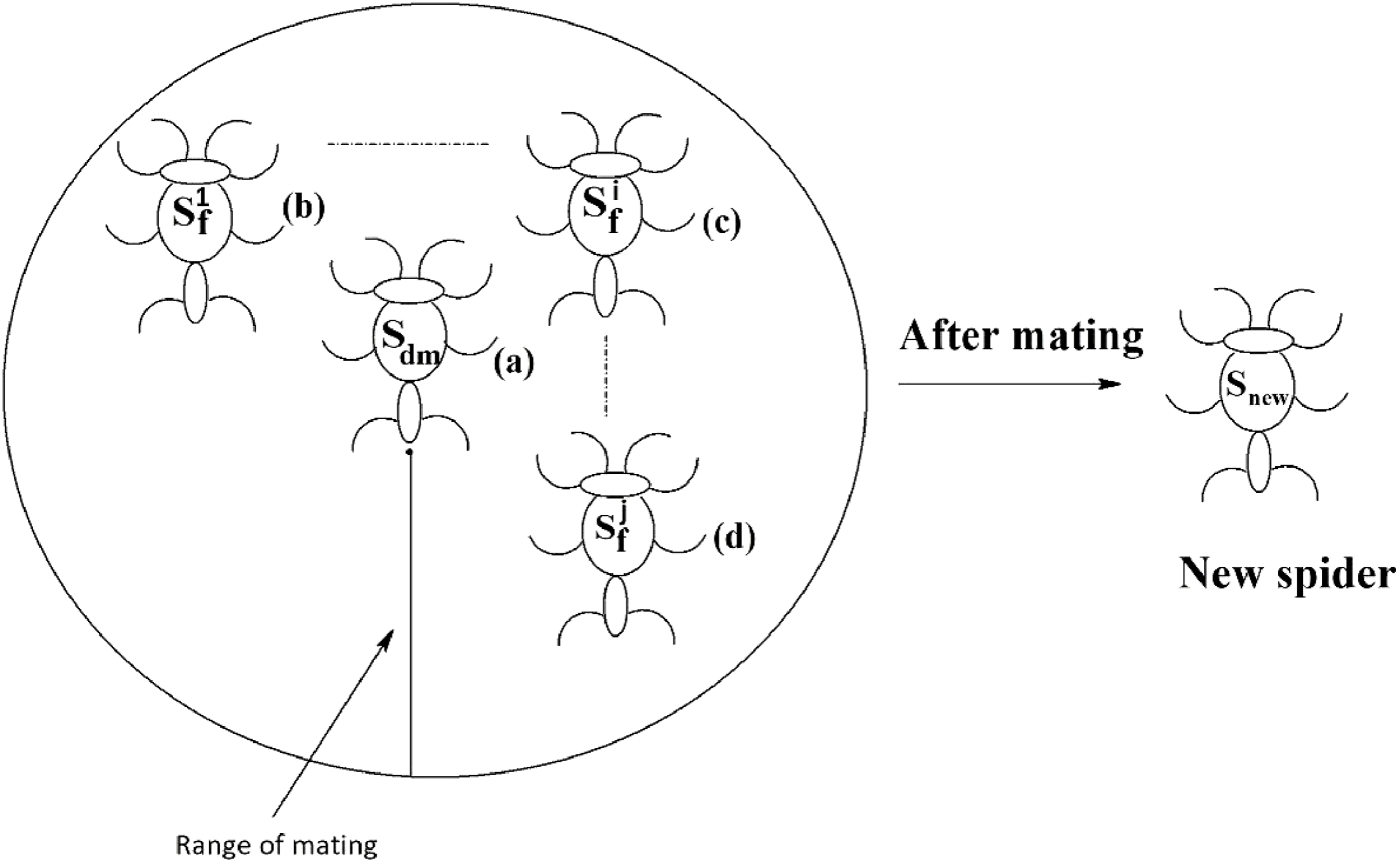
Mating of a dominant male spider in SSODCSC. The figure specifies how a dominant male spider mates with a set of female spiders to produce a new spider in SSODCSC.

Each dominant male spider mates with a set of female spiders within the specified range of mating to produce a new spider, as shown in Fig. 11. The new spider will be generated using the Roulette wheel method (Chandran, Reddy & Janet, 2018). If the weight of the new spider is better than the weight of worst spider, then the worst spider would be replaced by the new spider. The range of mating r is calculated using Eq. (13).

(13) $$r = {{{\rm{diff}}} \over {2*n}}$$

where diff is the sum of differences of the upper bound and lower bound of each dimension, and *n* is the number of dimensions of the dataset DS.

**Figure 12:**
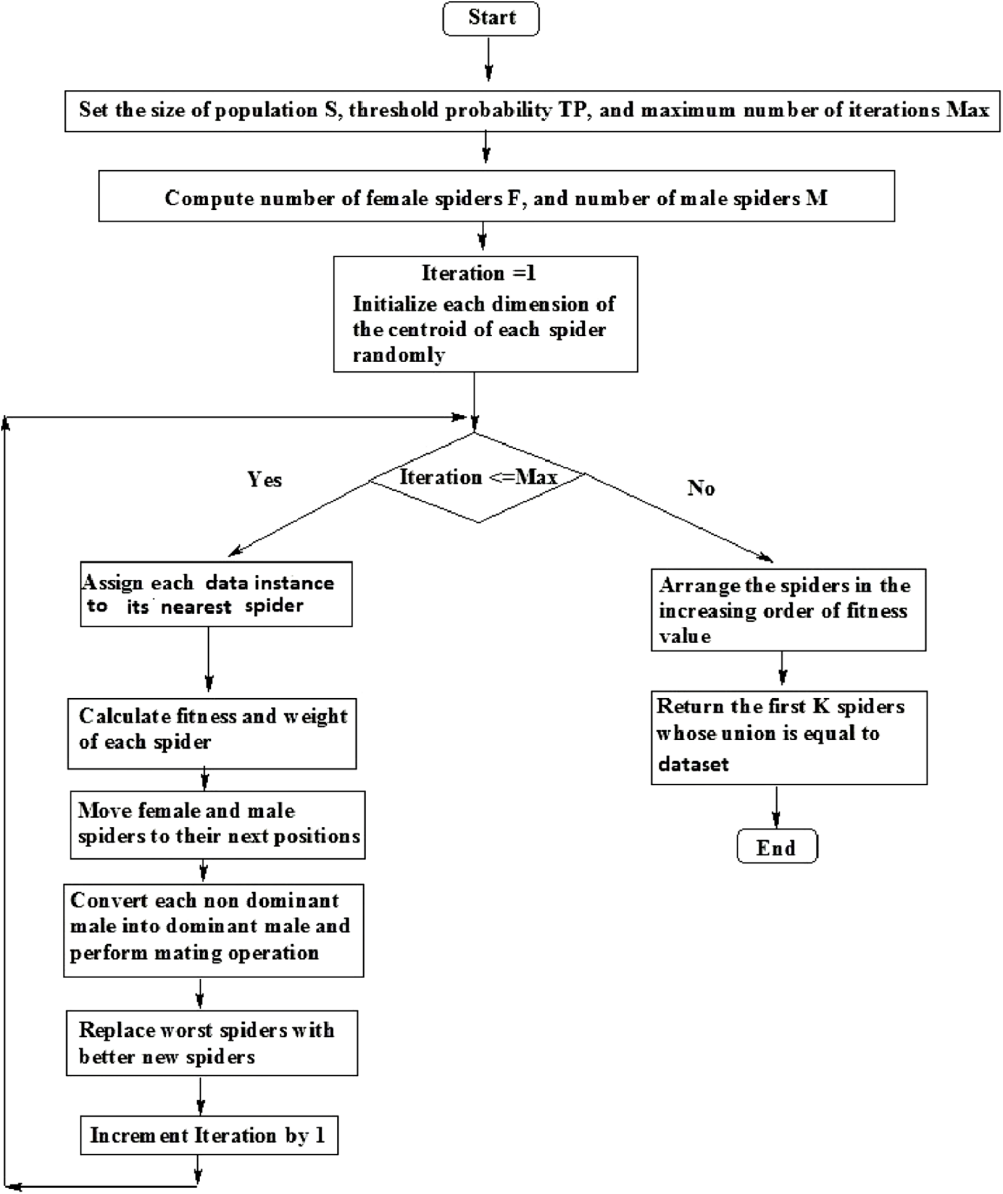
Flowchart of SSODCSC. The flowchart specifies the various steps in SSODCSC.

In SSO, the non-dominant male spiders are not allowed to mate with female spiders, as they would produce new spiders having low weights. In SSODCSC, a non-dominant male spider is converted into dominant male spider by making sure that its weight becomes greater than or equal to the average weight of dominant male spiders so that it participates in the mating process and produces a new spider whose weight is better than that of at least one other spider. The theoretical proof for the possibility of converting a non-dominant male spider into a dominant male spider is provided in Theorem 1. Thus, non-dominant male spiders become more powerful than dominant male spiders as they are made to produce new spiders that surely replace worst spiders in the population. The theoretical proof for the possibility of obtaining a new spider that is better than the worst spider, after a non-dominant male spider mates with the female spiders is provided in Theorem 2. The following steps are used to convert a non-dominant male spider into a dominant male spider:

Step 1: Create a list consisting of data instances of the non-dominant male spider *s_ndm_* in the decreasing order of their distances from its centroid.
Step 2: Delete the top-most data instance (i.e., the data instance which is the greatest distance from the centroid) from the list.
Step 3: Find the weight of the non-dominant male spider *s_ndm_*.
Step 4: If the weight of non-dominant male is less than the average weight of dominant male spiders, go to Step 2.
The flowchart for SSODCSC is specified in Fig. 12.
**Theorem 1.** *A non-dominant male spider can be converted into a dominant male spider in single centroid representation of SSO.*
**Proof:** Let *s_ndm_* be the non-dominant male spider whose weight is *w_ndm_*.
Let *medwgt* be the median weight of male spiders (which is always less than or equal to 1).
But according to definition of the non-dominant male spider,
  (14) $${w_{ndm}} < medwgt$$
Assume that the theorem is false.
⇒ *s_ndm_* can not be converted into a dominant male spider
⇒ During the movement of *s_ndm_* in the solution space,
  (15) $${w_{ndm}} < 1$$
Let Sum be the sum of distances of data instances from the centroid of *s_ndm_*.
If the data instance that is the furthest distance from the centroid of *s_ndm_* is removed from *s_ndm_*, then
⇒ sum of distances of data instances from the centroid of *s_ndm_* will decrease, as
Sum = Sum-distance of removed data instance from the centroid of *s_ndm_*.
⇒ fitness of *s_ndm_* will decrease, as
 fitness of *s_ndm_* is proportional to Sum
⇒ the weight of *s_ndm_* will increase as
 the weight of *s_ndm_* is inversely proportional to fitness of *s_ndm_*
Similarly,
  If a data instance is added to *s_ndm_*, then
⇒ sum of distances of data instances from centroid of *s_ndm_* will increase.
⇒ fitness of *s_ndm_* will increase.
⇒ the weight of *s_ndm_* will decrease.
Therefore,
  (16) $${w_{ndm}} = 1 - \sum\limits_{i = 1}^n {{w_{{d_i}}}} $$
  where 1 is the initial weight of *s_ndm_*, n is the total number of data instances added to *s_ndm_*, and ${w_{{d_i}}}$ is the decrease in the weight of *s_ndm_* when *i*th data instance was added to *s_ndm_*.
When all the data instances are removed from *s_ndm_*,
  (17) $${w_{ndm}} = {w_{ndm}} + \sum\limits_{i = 1}^n {{w_{{d_i}}} = 1 - } \sum\limits_{i = 1}^n {{w_{{d_i}}} + \sum\limits_{i = 1}^n {{w_{{d_i}}}} } $$
But according to Eq. (15), *w_ndm_* can never be 1 during the movement of *s_ndm_* in the solution space.
Hence, our assumption is wrong.
So, we can conclude that a non-dominant male spider can be converted into dominant male spider in single centroid representation of SSO.
**Theorem 2.** *The weight of the new spider resulting from the mating of a non-dominant male spider with a weight greater than or equal to the average weight of dominant male spiders will be better than at least one spider in the population.*
**Proof:** Let *s_ndm_* be the non-dominant male spider whose weight became greater than or equal to the average weight of dominant male spiders.
Let ${s_{{f_1}}},{s_{{f_2}}},{s_{{f_3}}}, \ldots \ldots ..,{s_{{f_m}}}$ be female spiders that participated in the mating.
Let *s*_new_ be the resulting new spider of the mating operation.
Let *N* be the total number of spiders in the colony.
Assume that the theorem is false.
It implies:
  (18) $\sum\limits_{i = 1}^N {{\rm{weight}}\left({{s_i}} \right) \le {\rm{weight}}\left({{s_{{\rm{new}}}}} \right)?1:0 = 0} $
In other words, the total number of spiders whose weight is less than or equal to that of *s*_new_ is zero.
But according to the Roulette wheel method:
  (19) $${s_{{\rm{new}}}} = {{\left( {\sum\nolimits_{i = 1}^m {{s_{{f_i}}}*{\rm{weight}}\left( {{s_{{f_i}}}} \right)} } \right) + {s_{ndm}}*{\rm{weight}}\left( {{s_{ndm}}} \right)} \over {\left( {\sum\nolimits_{i = 1}^m {{\rm{weight}}\left( {{s_{{f_i}}}} \right)} } \right) + {\rm{weight}}\left( {{s_{ndm}}} \right)}}$$
  ⇒ $\mathop {\lim }\limits_{{\rm{weight}}\left({{s_{{f_i}}}} \right) \to 0 \wedge {\rm{weight}}\left({{s_{ndm}}} \right) \to 1} {s_{{\rm{new}}}}$
  = *s_ndm_* with weight equal to 1 = *s_gbs_* (since any spider whose weight is 1 is always *s_gbs_*)
And
  $$\matrix{
 {\,\,\,\,\,\,\,\mathop {\lim }\limits_{{\rm{weight}}\left( {{s_{{f_i}}}} \right) \to 1 \wedge {\rm{weight}}\left( {{s_{ndm}}} \right) \to 1} {s_{{\rm{new}}}}} \hfill \cr 
 { = {{\left( {m\,\,{\rm{femalespiderswhoseweightis1}}} \right) + \left( {{s_{ndm}}\,{\rm{whoseweightis}}1} \right)} \over {m + 1}}} \hfill \cr 
 { = {{\left( {m + 1} \right)*{s_{gbs}}} \over {m + 1}}} \hfill \cr 
 { = {s_{gbs}}} \hfill \cr 
 } $$
So, when weight $\left( {{s_{{f_i}}}} \right)$ tends to 0, and weight (*s_ndm_*) tends to 1, *s*_new_ becomes *s_gbs_.*
When weight $\left( {{s_{{f_i}}}} \right)$ tends to 1, and weight (*s_ndm_*) (*s_ndm_*) tends to 1, *s*_new_ becomes *s_gbs_*.
Similarly,
  When weight $\left( {{s_{{f_i}}}} \right)$ tends to 1, and weight (*s_ndm_*) tends to 0, *s*_new_ becomes *s_gbs_*.
Substituting *s_gbs_* in place of *s*_new_ in Eq. (18),
  (20) $$\sum\limits_{i = 1}^N {{\rm{weight}}\left({{s_i}} \right) \le {\rm{weight}}\left({{s_{gbs}}} \right)?1:0 = 0} $$

According to Eq. (20), the number of spiders whose weight is less than or equal to the weight of *s_gbs_* is zero. But according to the definition of *s_gbs_*, its weight is greater than or equal to the weights of all remaining spiders. So, there are spiders whose weights are less than or equal to the weight of *s_gbs_*. Therefore Eq. (20) is false.

Hence, our assumption is wrong. Therefore, we can conclude that the weight of *s*_new_ produced by *s_ndm_* is greater than that of at least one spider in the population.

## Results

The proposed algorithm and the algorithms used in the comparison were implemented in the Java Run Time Environment, version 1.7.0.51, and the experiments were run on Intel Xeon CPU E3 1270 v3 with a 3.50-GHz processor with a 160 GB RAM. The Windows 7 Professional Operating System was used.

### Applying SSODCSC on patent datasets

At first, we applied SSODCSC on six Patent corpus datasets. The description of the data sets is given in Table 2. Patent corpus5000 contains 5,000 text documents with technical descriptions of the patents that belong to 50 different classes. Each class has exactly 100 text documents. Each text document contains only a technical description of the patent. All text documents were prepared using the ASCII format.

**Table 2:** Description of Patent corpus5000 datasets.

	Patent corpus1	Patent corpus2	Patent corpus3	Patent corpus4	Patent corpus5	Patent corpus6
Number of text documents	100	150	200	250	300	350
Number of clusters	6	7	9	9	8	7

As SSODCSC returned *K* best spiders, the SICD of clusters in those spiders was calculated. Table 3 specifies the clustering results of SSODCSC when applied on Patent corpus datasets. For each dataset the SICD value, Cosine similarity value, *F*-measure value and accuracy obtained were specified.

**Table 3:** Clustering results of SSODCSC: Patent corpus5000 datasets.

Dataset	SICD	Cosine similarity	*F*-Measure	Accuracy
Patent corpus1	10,263.55	0.8643	0.8666	87.53
Patent corpus2	12,813.98	0.7517	0.7611	79.24
Patent corpus3	16,600.41	0.7123	0.7316	74.29
Patent corpus4	20,580.11	0.9126	0.9315	94.05
Patent corpus5	23,163.24	0.8143	0.8255	83.17
Patent corpus6	28,426.86	0.8551	0.8703	86.25

Table 4 specifies the relationship between the SICD values and number of iterations. Lower SICD value indicate a higher clustering quality. It was found that as we increased the number of iterations, the SICD decreased and thereby, the clustering quality increased.

**Table 4:** Relationship between SICD and number of iterations: SSODCSC: Patent corpus5000 datasets.

Dataset	100 iterations	150 iterations	200 iterations	250 iterations	300 iterations
Patent corpus1	27,500.23	21,256.45	16,329.59	13,260.72	**10,263.55**
Patent corpus2	23,464.44	21,501.16	17,467.15	15,254.33	**12,813.98**
Patent corpus3	25,731.05	22,150.15	19,456.25	18,204.42	**16,600.41**
Patent corpus4	31,189.46	28,506.72	27,155.68	24,638.83	**20,580.11**
Patent corpus5	36,124.30	33,854.35	30,109.52	26,138.59	**23,163.24**
Patent corpus6	41,201.22	37,367.33	33,632.63	31,007.25	**28,426.86**

**Note:**

The best values are specified in bold.

To find the distance between data instances, we used the Euclidean distance function and Manhattan distance function. Data instances having small differences were placed in same cluster by the Euclidean distance function, as it ignores the small differences. It was found that SSODCSC produced a slightly better clustering result with the Euclidean distance function as shown in Table 5.

**Table 5:** Comparison between distance functions: SSODCSC: Patent corpus5000 datasets.

Dataset	Euclidean distance function		Manhattan distance function	
	Accuracy	Avg. cosine similarity	Accuracy	Avg. cosine similarity
Patent corpus1	**87.53**	**0.8643**	82.05	0.8198
Patent corpus2	**79.24**	**0.7517**	73.33	0.7344
Patent corpus3	**74.29**	**0.7123**	68.03	0.69.95
Patent corpus4	**94.05**	**0.9126**	85.27	0.8637
Patent corpus5	**83.17**	**0.8143**	76.49	0.7743
Patent corpus6	**86.25**	**0.8551**	80.46	0.8142

**Note:**

The best values are specified in bold.

Table 6 specifies the comparison between clustering algorithms with respect to SICD values. Table 7 specifies the comparison between clustering algorithms with respect to accuracy. SSODCSC produces better accuracy for all datasets. The overall percentage increase in the accuracy is approximately 13%.

**Table 6:** Comparison between clustering algorithms in terms of SICD: Patent corpus5000 datasets.

Dataset	*K*-means	PSO	GA	ABC	IBCO	ACO	SMSSO	BFGSA	SOS	SSO	SSODCSC
Patent corpus1	13,004.21	13,256.55	13,480.76	13,705.09	14,501.76	14,794.09	12,884.53	13,250.71	13,024.83	12,159.98	**10,263.55**
Patent corpus2	15,598.25	15,997.44	16,044.05	15,800.55	16,895.58	17,034.29	14,057.22	16,842.83	15,803.19	14,809.66	**12,813.98**
Patent corpus3	20,007.12	21,255.77	23,903.11	24,589.19	19,956.44	19,543.05	18,183.14	21,259.03	19,045.42	18,656.93	**16,600.41**
Patent corpus4	24,175.19	25,023.52	27,936.76	28,409.58	24,498.32	25,759.48	23,637.83	25,109.06	24,264.31	23,447.12	**20,580.11**
Patent corpus5	31,064.62	29,879.76	31,007.15	31,588.66	27,442.28	30,015.64	28,268.55	30,129.24	29,176.48	26,289.88	**23,163.24**
Patent corpus6	29,846.53	32,226.51	33,509.84	34,185.35	31,993.79	32,753.55	30,005.81	32,208.31	31,804.89	31,615.35	**28,426.86**

**Note:**

The best values are specified in bold.

**Table 7:** Comparison of clustering algorithms in terms of accuracy: Patent corpus5000 datasets.

Dataset	*K*-means	PSO	GA	ABC	IBCO	ACO	SMSSO	BFGSA	SOS	SSO	SSODCSC
Patent corpus1	68.29	57.97	57.06	58.26	56.08	54.15	68.28	76.03	49.14	70.22	**87.53**
Patent corpus2	70.21	69.38	67.15	68.57	67.88	67.05	62.25	69.92	60.05	69.45	**79.24**
Patent corpus3	65.15	64.95	62.99	63.25	67.09	66.98	51.19	68.28	64.03	67.69	**74.29**
Patent corpus4	64.93	61.03	58.78	59.11	58.12	69.49	55.28	68.87	62.49	71.10	**84.05**
Patent corpus5	69.72	57.38	55.80	56.07	44.67	68.05	61.51	64.62	68.55	71.16	**83.17**
Patent corpus6	58.35	62.59	60.65	61.47	54.95	69.51	64.63	69.55	72.01	70.29	**85.25**

**Note:**

The best values are specified in bold.

The silhouette coefficient SC of a data instance *d_i_* can be calculated using Eq. (21).

(21) $${\rm{SC}} = {{b - a} \over {{\rm{max}}\ \left( {a,b} \right)}}$$

where *a* is the average of distances between data instance *d_i_* and other data instances present in its containing cluster, and *b* is the minimum of distances between data instance *d_i_* and data instances present in other clusters. The range of the silhouette coefficient is [−1, 1]. When it is closer to 1, better clustering results will be produced. Table 8 specifies the comparison between clustering algorithms with respect to the average silhouette coefficient values of datasets. SSODCSC produces better average silhouette coefficient values for all Patent corpus datasets.

**Table 8:** Average silhouette coefficient value: Patent corpus datasets.

Dataset	*K*-means	PSO	GA	ABC	IBCO	ACO	SMSSO	BFGSA	SOS	SSO	SSODCSC
Patent corpus1	0.5043	0.5990	0.4001	0.4109	0.4844	0.3184	0.4908	0.7015	0.5804	0.7159	**0.7405**
Patent corpus2	0.5107	0.6220	0.5922	0.3906	0.4335	0.4577	0.5388	0.6799	0.6496	0.6884	**0.7797**
Patent corpus3	0.4498	0.4411	0.4804	0.4188	0.5913	0.4990	0.6588	0.6731	0.6005	0.6691	**0.7551**
Patent corpus4	0.3466	0.6618	0.5269	0.4401	0.4548	0.4018	0.6106	0.7177	0.5985	0.6994	**0.8009**
Patent corpus5	0.4082	0.3933	0.4005	0.4905	0.3997	0.4833	0.6933	0.7269	0.6208	0.7280	**0.7648**
Patent corpus6	0.3225	0.4119	0.5507	0.5055	0.4883	0.4397	0.7045	0.6894	0.7328	0.7448	**0.8397**

**Note:**

The best values are specified in bold.

**Figure 13:**
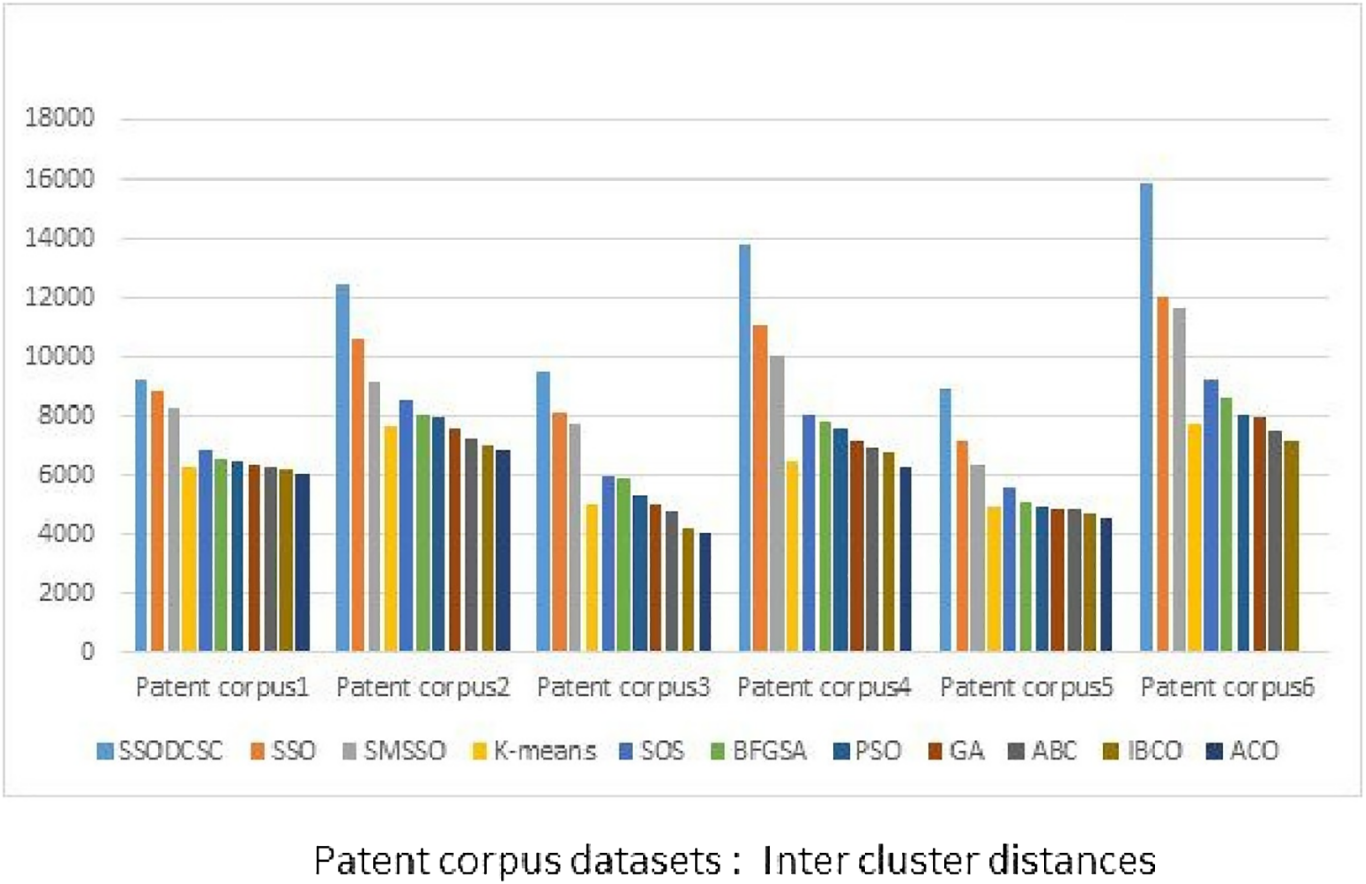
Inter-cluster distances: Patent corpus5000 datasets. The figure specifies inter-cluster distances returned by clustering algorithms when applied on Patent corpus5000 datasets.

**Figure 14:**
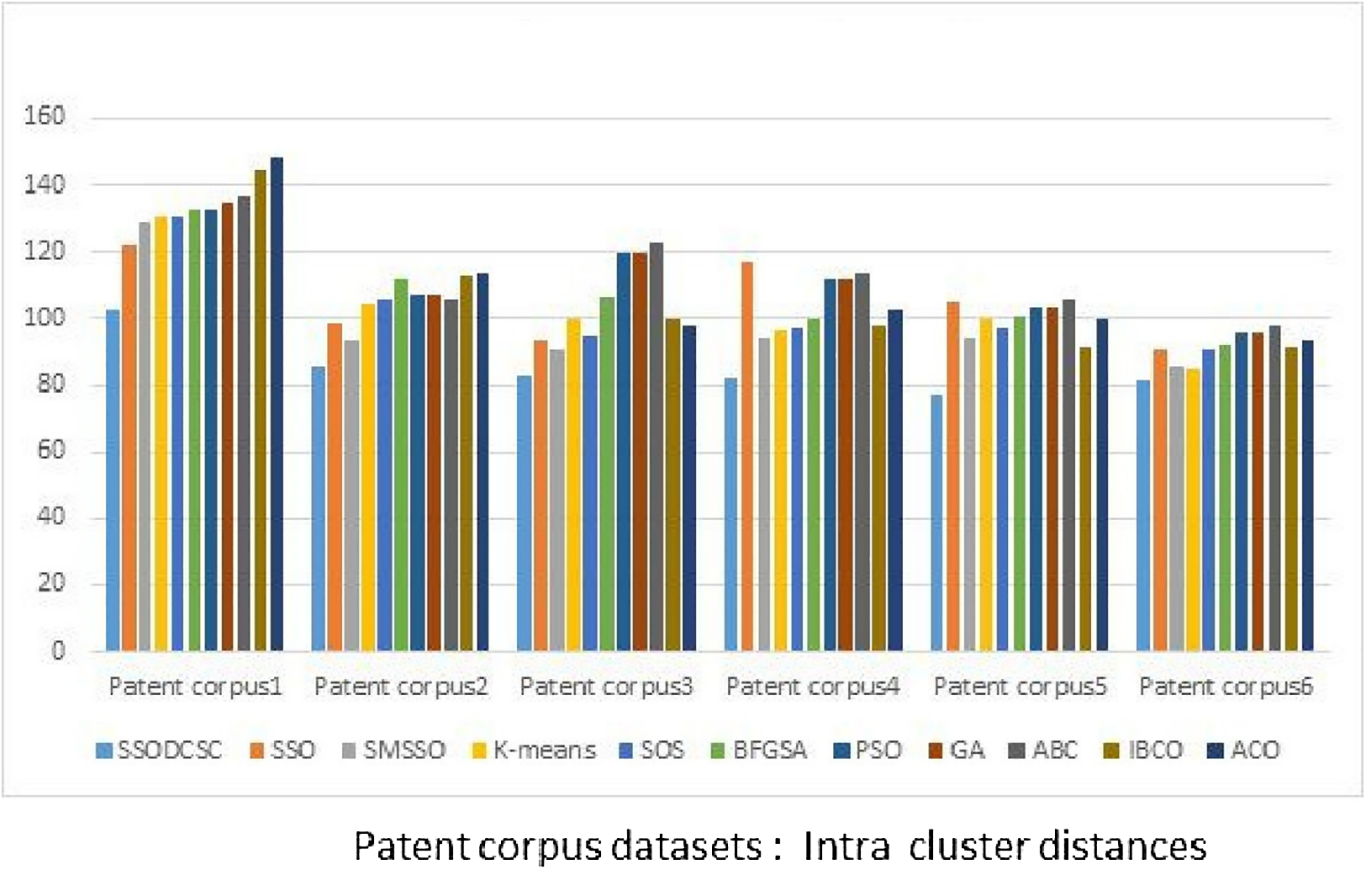
Intra-cluster distances: Patent corpus5000 datasets. The figure specifies intra-cluster distances returned by clustering algorithms when applied on Patent corpus5000 datasets.

From Figs. 13 to 14, it is obvious that SSODCSC produces the largest inter-cluster distances and smallest ICD for Patent corpus datasets.

### Applying SSODCSC on UCI datasets

We applied SSODCSC on UCI data sets as well. The description of the data sets is given in Table 9. Table 10 specifies the relationship between SICD values and the number of iterations. As we increase the number of iterations, the SICD is also reduced. For the Iris dataset, the SICD value is 125.7045 at 100 iterations but as we increase the number of iterations, the SICD value of the clustering result also decreases until it reaches 95.2579 at 300 iterations. However, it remains at 95.2579, after 300 iterations and it becomes obvious that SSODCSC converges in 300 iterations.

**Table 9:** Description of UCI datasets.

Dataset	Number of classes	Number of attributes	Number of instances
Iris	3	4	150
Wine	3	13	178
Glass	6	9	214
Vowel	6	3	871
Cancer	2	9	683
CMC	3	9	1,473
Haberman	2	3	306
Bupa	2	6	345

**Table 10:** Relationship between SICD and number of iterations: SSODCSC: UCI datasets.

Dataset	100 iterations	150 iterations	200 iterations	250 iterations	300 iterations
Iris	125.7045	118.9034	107.0844	100.3683	**95.2579**
Vowel	147,257.5582	147,001.1863	146,948.7469	146,893.7569	**146,859.1084**
CMC	6,206.8186	6,127.4439	5,986.2964	5,574.6241	**5,501.2642**
Glass	387.5241	340.3885	301.0084	258.3053	**207.2091**
Wine	17,358.0946	17,150.6084	16,998.4387	16,408.5572	**16,270.1427**

**Note:**

The best values are specified in bold.

Table 11 specifies the best three spiders for the Iris dataset. We initialized a solution space with 50 spiders, among which, the first 30 spiders were females and the remaining were males. Our proposed algorithm returned spider 21, spider 35, and spider 16. Spider 21 and spider 16 were females and spider 35 was a male spider. The centroids of these spiders were (6.7026, 3.0001, 5.4820, 2.018), (5.193, 3.5821, 1.4802, 0.2402), and (5.8849, 2.8009, 4.4045, 1.4152), respectively. These centroids have four values as Iris dataset consists of four attributes. The sum of the distances between the 150 data instances present in the Iris dataset and their nearest centroids in Table 11 was found to be 95.2579, as shown in Table 10.

**Table 11:** Best three spiders of Iris dataset: SSODCSC.

Best spiders	Dimension 1	Dimension 2	Dimension 3	Dimension 4
Spider 21	6.7026	3.0001	5.482	2.018
Spider 35	5.193	3.5821	1.4802	0.2402
Spider 16	5.8849	2.8009	4.4045	1.4152

Table 12 specifies the best six spiders for vowel dataset. Our proposed algorithm returned spider 10, spider 25, spider 42, spider 22, spider 48, and spider 5. Spider 10, spider 25, spider 22, and spider 5 were females, and spiders 42 and spider 48 were male spiders. The sum of the distance between the 871 data instances present in the Vowel data set and their nearest centroids in Table 12 was found to be 146,859.1084, as shown in Table 10.

**Table 12:** Best six spiders of Vowel dataset: SSODCSC.

Best spiders	Dimension 1	Dimension 2	Dimension 3
Spider 10	508.4185	1,838.7035	2,558.1605
Spider 25	408.0024	1,013.0002	2,310.9836
Spider 42	624.0367	1,308.0523	2,333.8023
Spider 22	357.1078	2,292.1580	2,976.9458
Spider 48	377.2070	2,150.0418	2,678.0003
Spider 5	436.8024	993.0034	2,659.0012

Table 13 specifies the best three spiders for the CMC dataset. Our proposed algorithm returned spider 23, spider 38, and spider 16 among which spider 23 and spider 16 were females and spider 38 was a male spider. The centroids of these spiders are specified. These centroids had nine values as the CMC dataset consists of nine attributes. The sum of the distance between the 1,473 data instances present in the CMC data set and their nearest centroids in Table 13 was found to be 5,501.2642, as shown in Table 10.

**Table 13:** Best three spiders of CMC dataset: SSODCSC.

Best spiders	Dimension 1	Dimension 2	Dimension 3	Dimension 4	Dimension 5	Dimension 6	Dimension 7	Dimension 8	Dimension 9
Spider 23	24.4001	3.0699	3.4986	1.8021	0.9303	0.8206	2.2985	2.9584	0.0271
Spider 38	43.7015	2.9929	3.4602	3.4568	0.8209	0.8330	1.8215	3.4719	3.306
Spider 16	33.4894	3.0934	3.5599	3.5844	0.8015	0.6629	2.169	3.2901	0.0704

Tables 14 and 15 specify the best spiders and their centroids for the Glass and Wine datasets, respectively. The sum of the distances between the 214 data instances present in the Glass dataset and their nearest centroids in Table 14 was found to be equal to 207.2091, as shown in Table 10. If we find the sum of the distances between the 178 data instances present in the Wine dataset and their nearest centroids in Table 15, it would be equal to 16,270.1427, as shown in Table 10.

**Table 14:** Best six spiders of Glass dataset: SSODCSC.

Best spiders	Dimension 1	Dimension 2	Dimension 3	Dimension 4	Dimension 5	Dimension 6	Dimension 7	Dimension 8	Dimension 9
Spider 13	1.5201	14.6023	0.06803	2.2617	73.3078	0.0094	8.7136	1.01392	0.0125
Spider 29	1.5306	13.8005	3.5613	0.9603	71.8448	0.1918	9.5572	0.0827	0.0071
Spider 35	1.5169	13.3158	3.6034	1.4236	72.7014	0.5771	8.2178	0.0076	0.0321
Spider 42	1.4138	13.0092	0.0036	3.0253	70.6672	6.2470	6.9489	0.0078	0.0004
Spider 48	1.5205	12.8409	3.4601	1.3091	73.0315	0.6178	8.5902	0.0289	0.0579
Spider 7	1.5214	13.0315	0.2703	1.5193	72.7601	0.3615	11.995	0.0472	0.0309

**Table 15:** Best three spiders of Wine dataset: SSODCSC.

Best spiders	Dimension 1	Dimension 2	Dimension 3	Dimension 4	Dimension 5	Dimension 6	Dimension 7	Dimension 8	Dimension 9	Dimension 10	Dimension 11	Dimension 12	Dimension 13
Spider 33	12.89	2.12	2.41	19.51	98.89	2.06	1.46	0.47	1.52	5.41	0.89	2.15	686.95
Spider 4	12.68	2.45	2.41	21.31	92.41	2.13	1.62	0.45	1.14	4.92	0.82	2.71	463.71
Spider 3	13.37	2.31	2.62	17.38	105.08	2.85	3.28	0.29	2.67	5.29	1.04	3.39	1,137.5

Table 16 specifies the average CPU time per iteration (in seconds), when clustering algorithms were applied on the CMC dataset. It was found that SSODCSC produces clustering results with the shortest average CPU time per iteration.

**Table 16:** Comparison of clustering algorithms in terms of average CPU time per iteration (in seconds): CMC dataset.

	*K*-means	PSO	IBCO	ACO	SMSSO	BFGSA	SOS	SSO	SSODCSC
Best	0.0041	0.0151	0.0168	0.0186	0.0097	0.0148	0.0116	0.0065	0.0048
Average	0.0068	0.0192	0.0205	0.0215	0.0126	0.0172	0.0129	0.0082	0.0055
Worst	0.0072	0.0235	0.0245	0.0278	0.0138	0.0194	0.0144	0.0097	0.0069

Table 17 specifies the average CPU time per iteration (in seconds), when clustering algorithms were applied on the Vowel dataset. It was found that *K*-means produces clustering results with the shortest average CPU time per iteration. The SSODCSC has the second shortest average CPU time per iteration.

**Table 17:** Comparison of clustering algorithms in terms of average CPU time per iteration (in seconds): Vowel dataset.

	*K*-means	PSO	IBCO	ACO	SMSSO	BFGSA	SOS	SSO	SSODCSC
Best	0.0125	0.1263	0.1455	0.1602	0.0188	0.0206	0.0215	0.0178	0.0145
Average	0.0136	0.1923	0.2034	0.1698	0.0204	0.0218	0.0228	0.0195	0.0172
Worst	0.0155	0.2056	0.2245	0.1893	0.0219	0.0231	0.0239	0.0227	0.0198

Table 18 specifies *F*-measure values obtained by the clustering algorithms when they are applied on the Iris, Glass, Vowel, Wine, Cancer, and CMC datasets, respectively. It is evident that SSODCSC produces the best *F*-measure values. The overall percentage increase in the *F*-measure value is approximately 10%.

**Table 18:** Comparison of clustering algorithms in terms of *F*-measure values: UCI datasets.

Dataset	*K*-means	PSO	GA	ABC	IBCO	ACO	SMSSO	BFGSA	SOS	SSO	SSODCSC
Wine	82.25	78.79	70.25	72.48	63.34	64.88	60.10	67.88	63.78	78.42	**94.98**
Cancer	76.95	83.42	71.38	70.55	62.98	60.34	61.95	62.03	64.80	74.34	**96.49**
CMC	50.25	51.49	55.15	57.79	51.92	50.49	51.98	52.92	52.00	51.45	**61.01**
Vowel	66.10	68.11	60.69	64.74	62.12	68.13	54.00	68.68	65.56	70.85	**90.46**
Iris	94.43	90.95	62.41	62.58	60.43	71.95	64.43	62.47	62.43	85.81	**96.95**
Glass	52.88	44.94	45.01	43.72	54.66	43.36	55.48	42.21	44.46	58.54	**70.92**

**Note:**

The best values are specified in bold.

**Figure 15:**
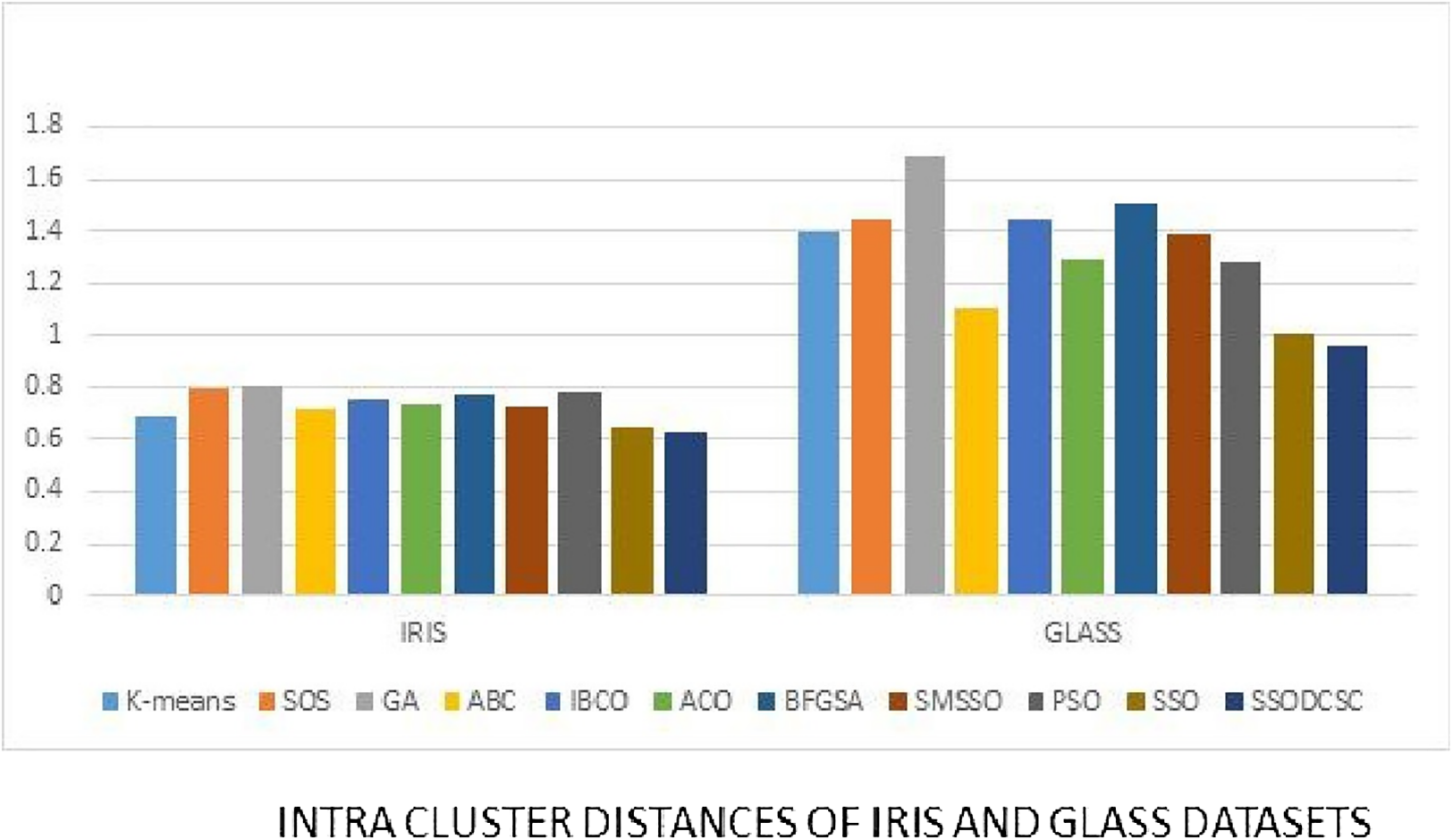
Intra-cluster distances: UCI datasets: Iris and Glass datasets. The figure compares the clustering algorithms based on intra-cluster distances when applied on Iris and Glass datasets.

**Figure 16:**
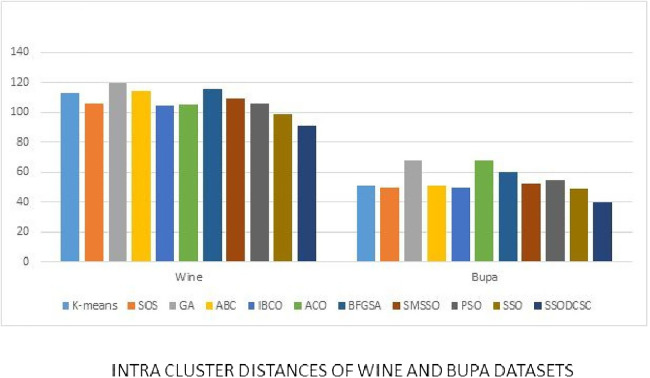
Intra-cluster distances: UCI datasets: Wine and Bupa datasets. The figure compares intra-cluster distances of clustering algorithms when applied on Wine and Bupa datasets.

**Figure 17:**
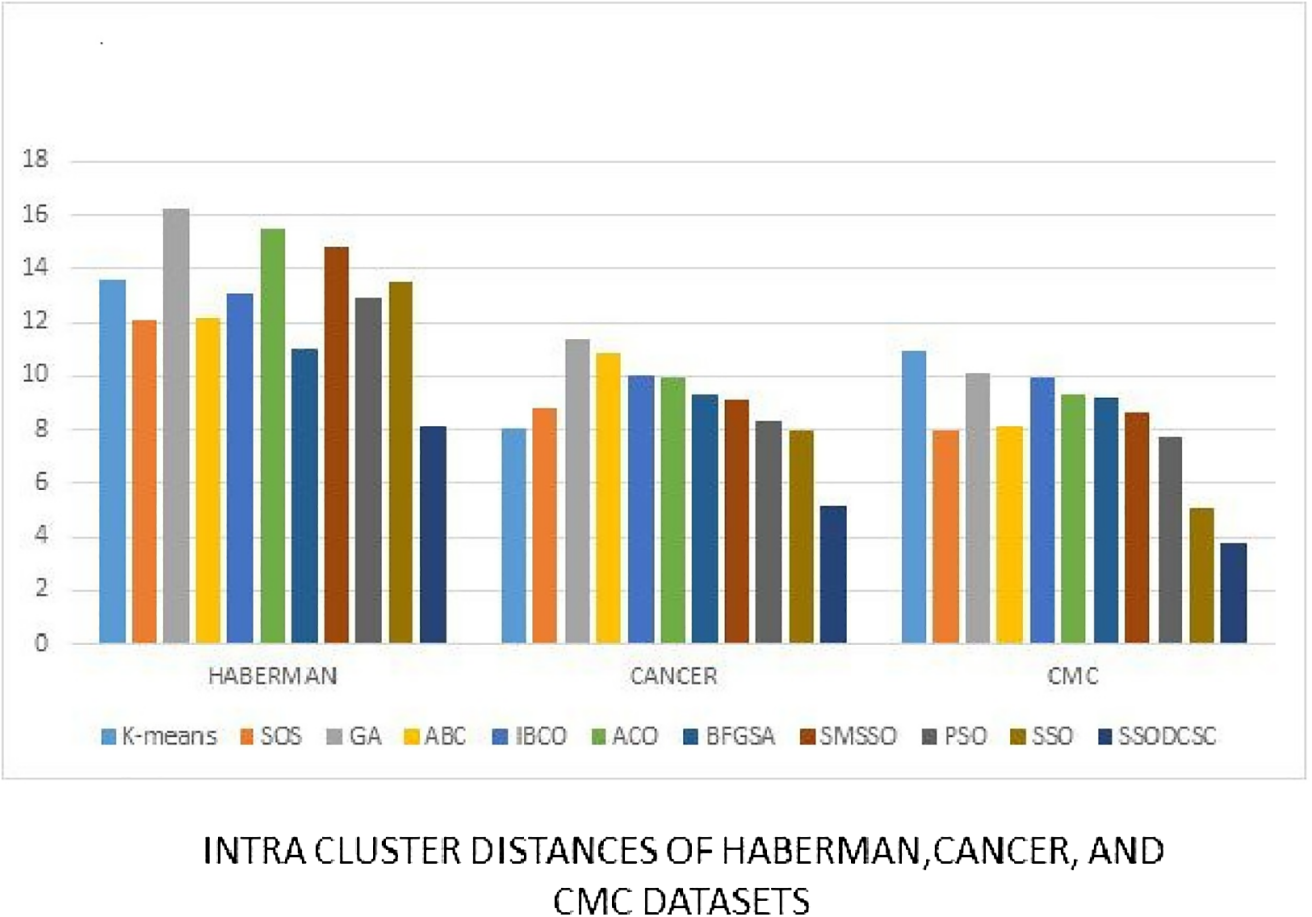
Intra-cluster distances: UCI datasets: Haberman, Cancer, and CMC datasets. The figure compares intra-cluster distances of clustering algorithms when applied on Haberman, Cancer, and CMC datasets.

**Figure 18:**
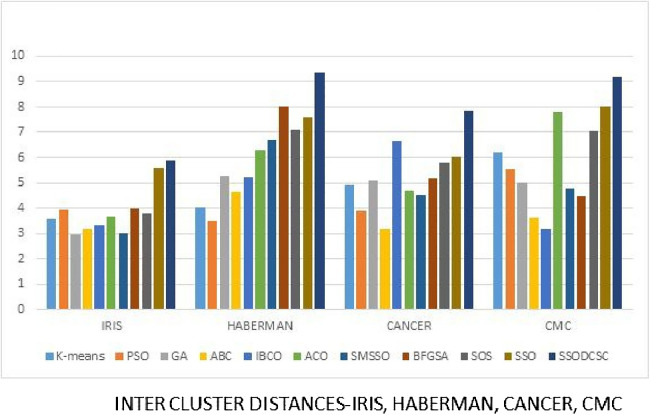
Inter-cluster distances: UCI datasets: Iris, Haberman, Cancer, and CMC. The figure compares inter-cluster distances of clustering algorithms when applied on Iris, Haberman, Cancer, and CMC datasets.

**Figure 19:**
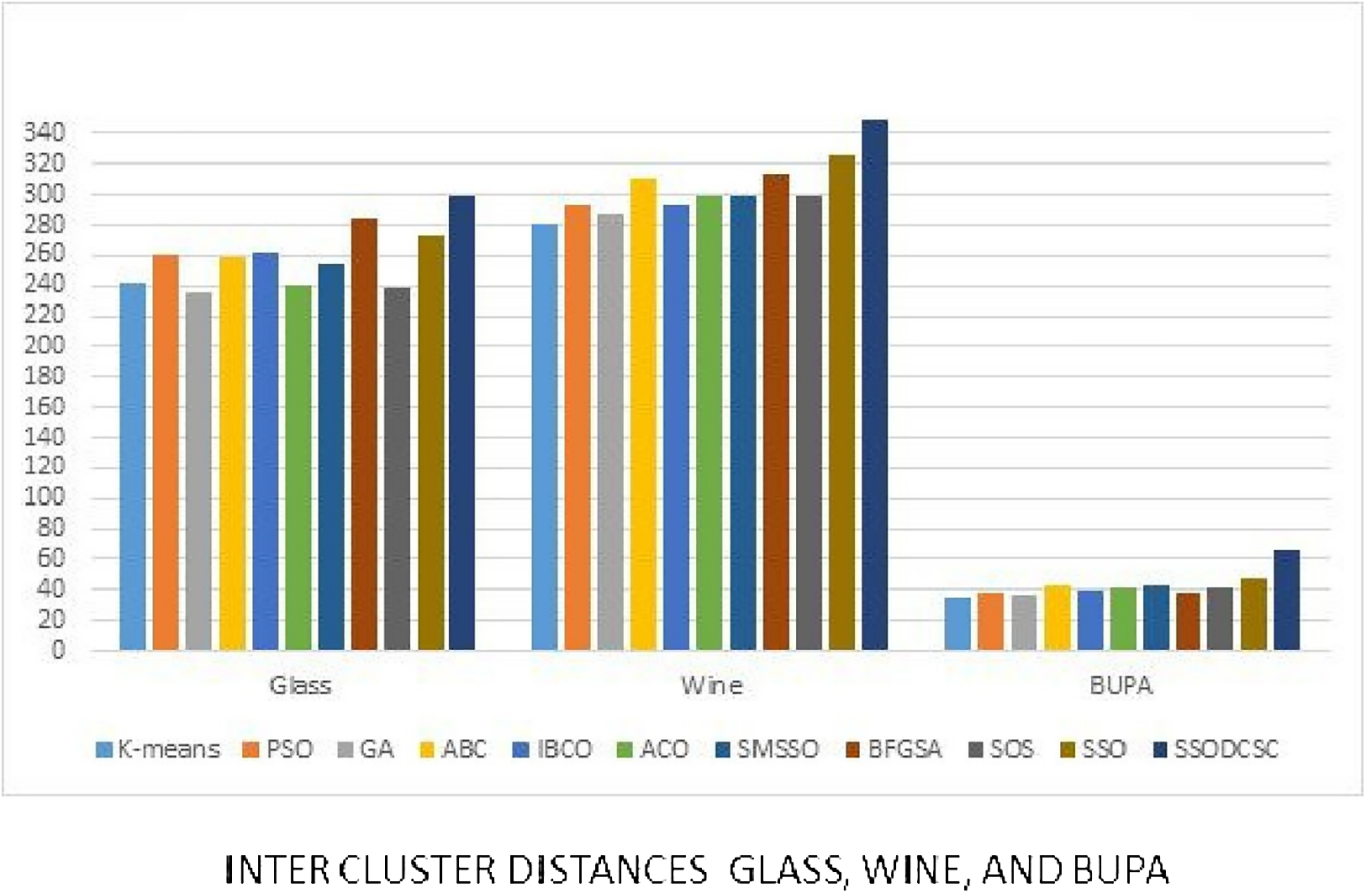
Inter-cluster distances: UCI datasets: Glass, Wine, and Bupa datasets. The figure compares inter-cluster distances of clustering algorithms when applied on Glass, Wine, and Bupa datasets.

We computed the ICD and inter-cluster distances of the resultant clusters of the clustering algorithms when applied on UCI datasets. From Figs. 15 to 17 it is obvious that SSODCSC produces the smallest ICD for UCI datasets. From Figs. 18 to 19 it can be concluded that SSODCSC produces the largest inter-cluster distances for UCI datasets, as compared with other clustering algorithms.

Table 19 specifies the comparison between clustering algorithms with respect to the average silhouette coefficient values of UCI datasets. SSODCSC produces better average silhouette coefficient values for UCI datasets also. The average silhouette coefficient values produced by SSODCSC are 0.7505, 0.6966, 0.7889, 0.7148, 0.8833, and 0.6264 for Wine, Cancer, CMC, Vowel, Iris, and Glass datasets, respectively.

**Table 19:** Average silhouette coefficient value: UCI datasets.

Dataset	*K*-means	PSO	GA	ABC	IBCO	ACO	SMSSO	BFGSA	SOS	SSO	SSODCSC
Wine	0.6490	0.5629	0.5008	0.5226	0.4151	0.4488	0.6003	0.6109	0.6417	0.6885	**0.7505**
Cancer	0.5894	0.6228	0.5277	0.5848	0.4492	0.4852	0.5995	0.5651	0.5999	0.6107	**0.6966**
CMC	0.3733	0.3281	0.3162	0.3726	0.3447	0.4984	0.4805	0.4900	0.4788	0.5111	**0.7889**
Vowel	0.4588	0.4079	0.4277	0.4011	0.6212	0.4105	0.5822	0.6255	0.6020	0.6492	**0.7148**
Iris	0.7099	0.7165	0.4388	0.4736	0.6043	0.5059	0.6253	0.5796	0.6511	0.6333	**0.8833**
Glass	0.3661	0.2805	0.2996	0.2070	0.5466	0.2900	0.4896	0.4155	0.4011	0.4419	**0.6264**

**Note:**

The best values are specified in bold.

### Statistical analysis: Patent corpus datasets

To show the significance of the proposed algorithm, we applied a one-way ANOVA test on the accuracy values shown in Table 7. Sum, Sum squared, Mean, and Variance of the clustering algorithms are specified in Table 20.

**Table 20:** Statistical results of one-way ANOVA test when applied on accuracy values returned by clustering algorithms for Patent corpus5000 datasets.

Dataset	Sum	Sum squared	Mean	Variance
*K*-means	396.650	26,318.996	66.108	19.425
PSO	373.300	23,327.241	62.217	20.352
GA	362.430	21,979.857	60.405	17.455
ABC	366.730	22,513.033	61.122	19.577
IBCO	348.790	20,646.575	58.132	74.166
ACO	395.230	26,205.548	65.872	34.218
SMSSO	363.140	22,174.032	60.523	39.118
BFGSA	417.270	29,087.550	69.545	13.701
SOS	376.270	23,910.126	62.712	62.721
SSO	419.910	29,395.727	69.985	1.665
SSODCSC	493.530	40,708.696	69.985	22.677

Degrees of freedom: df1 = 10, df2 = 55
Sum of squares for treatment (SSTR) = 2,761.313
Sum of squares for error (SSE) = 1,625.378
Total sum of squares (SST = SSE + SSTR) = 4,386.691
Mean square treatment (MSTR = SSTR/df1) = 276.131
Mean square error (MSE = SSE/df2) = 29.552
*F* (= MSTR/MSE) = 9.344
Probability of calculated *F* = 0.0000000080
*F* critical (5% one tailed) = 2.008
We can reject the null hypothesis as calculated *F* (9.344) is greater than *F* critical (2.008).

1) Post-hoc analysis using Tukeys’ honestly significant difference method:
Assuming significance level of 5%.
Studentized range for df1 = 10 and df2 = 55 is 4.663.
Tukey honestly significant difference = 10.349.

Mean of *K*-means and SSODCSC differs as 16.14667 is greater than 10.349.
Mean of PSO and SSODCSC differs as 20.03833 is greater than 10.349.
Mean of genetic algorithms (GA) and SSODCSC differs as 21.85000 is greater than 10.349.
Mean of artificial bee colony (ABC) and SSODCSC differs as 21.13333 is greater than 10.349.
Mean of improved bee colony optimization (IBCO) and SSODCSC differs as 24.12333 is greater than 10.349.
Mean of ACO and SSODCSC differs as 16.38333 is greater than 10.349.
Mean of SMSSO and SSODCSC differs as 21.73167 is greater than 10.349.
Mean of BFGSA and SSODCSC differs as 12.71000 is greater than 10.349.
Mean of SOS and SSODCSC differs as 19.54333 is greater than 10.349.
Mean of SSO and SSODCSC differs as 12.27000 is greater than 10.349.
Therefore, it may be concluded that SSODCSC significantly differs from other clustering algorithms.

### Statistical analysis: UCI datasets

To show the significance of the proposed algorithm, we applied a one-way ANOVA test on the *F*-measure values shown in Table 17. Sum, Sum squared, Mean, and Variance of the clustering algorithms are specified in Table 21.

**Table 21:** Statistical results of one-way ANOVA test when applied on *F*-measure values returned by clustering algorithms for UCI datasets.

Dataset	Sum	Sum squared	Mean	Variance
*K*-means	422.860	31,293.957	70.477	298.439
PSO	417.700	30,748.459	69.617	333.915
GA	364.890	22,675.874	60.815	97.018
ABC	371.860	23,589.299	61.977	108.531
IBCO	355.450	21,172.517	59.242	23.013
ACO	359.150	22,098.159	59.858	120.008
SMSSO	347.940	20,296.988	57.990	23.990
BFGSA	356.190	21,657.069	59.365	102.370
SOS	353.030	21,143.238	58.939	74.308
SSO	419.410	30,133.245	69.902	163.157
SSODCSC	510.810	44,665.701	85.135	235.578

Degrees of freedom: df1 = 10, df2 = 55.
Sum of squares for treatment (SSTR) = 4,113.431.
Sum of squares for error (SSE) = 7,901.638.
Total sum of squares (SST = SSE + SSTR) = 12,015.069.
Mean square treatment (MSTR = SSTR/df1) = 411.343.
Mean square error (MSE = SSE/df2) = 143.666.
*F* (= MSTR/MSE) = 2.863.
Probability of calculated *F* = 0.0060723031.
*F* critical (5% one tailed) = 2.008.
So, we can reject the null hypothesis as calculated *F* (2.863) is greater than *F* critical (2.008).
1) Post-hoc analysis using Tukeys’ honestly significant difference method
Assuming significance level of 5%.
Studentized range for df1 = 10 and df2 = 55 is 4.663.
Tukey honestly significant difference = 22.819.
Means of GA and SSODCSC differ as 24.32000 is greater than 22.819.
Means of ABC and SSODCSC differ as 23.15833 is greater than 22.819.
Means of IBCO and SSODCSC differ as 25.89333 is greater than 22.819.
Means of ACO and SSODCSC differ as 25.27667 is greater than 22.819.
Means of SMSSO and SSODCSC differ as 27.14500 is greater than 22.819.
Means of BFGSA and SSODCSC differ as 25.77000 is greater than 22.819.
Means of SOS and SSODCSC differ as 26.29667 is greater than 22.819.

Therefore, it is obvious that SSODCSC significantly differs from most of the other clustering algorithms when applied on UCI datasets.

## Discussion

We applied our proposed algorithm on Patent corpus datasets (PatentCorpus5000, 2012) and UCI datasets (Lickman, 2013). If the population size is small then the optimal solution is hard to find. If it is large, then the optimal solution is guaranteed with a side effect of higher computational complexity. We used 50 spiders to obtain the optimal solution without the side effect of higher computational complexity. Among these spiders, 70% were female spiders and the remaining were male spiders. The TP value was set to 0.7.

We compared the clustering results of SSODCSC with other clustering algorithms such as *K*-means, PSO, GA, ABC optimization, ACO, IBCO (Forsati, Keikha & Shamsfard, 2015), SMSSO, BFGSA, SOS, and SSO implementation in which each spider is a collection of *K* centroids, and found that SSODCSC produces better clustering results.

In order to conduct experiments, we formed the Patent corpus1 dataset by taking 100 text documents that belong to six different classes, Patent corpus2 dataset by taking 150 text documents that belong to seven different classes, Patent corpus3 dataset by taking 200 text documents that belong to nine different classes, Patent corpus4 dataset by taking 250 text documents that belong to nine different classes, Patent corpus5 dataset by taking 300 text documents that belong to eight different classes, and Patent corpus6 dataset by taking 350 text documents that belong to seven different classes of Patent corpus5000 data repository.

The clustering quality can be validated using ICD and inter-cluster distances. The smaller value for intra-cluster distance and a larger value for inter-cluster distance are the requirements for any clustering algorithm. We computed the ICD and inter-cluster distances of the resultant clusters of the clustering algorithms, when applied on Patent corpus datasets and UCI datasets, and found that SSODCSC produces better results than the other clustering algorithms.

We compared the clustering algorithms on the basis of average CPU time per iteration (in seconds). We found that SSODCSC has the shortest average CPU time per iteration with respect to most of the datasets. The reasons for this are its ability to produce a better solution space after every iteration, to initialize the solution space in less time, to compute fitness values of the spiders in less time, and to find the next positions of the spiders in less time.

We compared the clustering algorithms on the basis of the average silhouette coefficient value. We found that SSODCSC produces better average silhouette coefficient values for both Patent corpus datasets and UCI datasets.

We conducted a one-way ANOVA test separately on the clustering results of Patent corpus datasets and UCI datasets to show the superiority and applicability of the proposed method with respect to text datasets and feature based datasets.

## Conclusion

In this paper, we proposed a novel implementation of SSO for data clustering using a single centroid representation and enhanced mating. Additionally, we allowed non-dominant male spiders to mate with female spiders by converting them into dominant males. As a result, the explorative power of the algorithm has been increased and thereby the chance of getting a global optimum has been improved. We compared SSODCSC with other state-of-the-art algorithms and found that it produces better clustering results. We applied SSODCSC on Patent corpus text datasets and UCI datasets and got better clustering results than other algorithms. We conducted a one-way ANOVA test to show its superiority and applicability with respect to text datasets and feature-based datasets. Future work will include the study of applicability of SSODCSC in data classification of brain computer interfaces.

## Supplemental Information

10.7717/peerj-cs.201/supp-1 Supplemental Information 1 PATENTCORPUSandUCI. The raw data used in the experiments.

10.7717/peerj-cs.201/supp-2 Supplemental Information 2 SSODCSC SOURCE CODE. SSODCSC was written in Java. To run it, only JDK is required.The data files should be stored with .txt extension in current working directory. The program works for both text datasets and attribute based datasets.

## Additional Information and Declarations

### Data Availability

The following information was supplied regarding data availability: The two datasets are available in the Supplemental Files. The proposed algorithm was applied on text data files in PATENTCORPUS5000 datasets and feature/attribute-based UCI datasets.
