# Supplementary material for: A novel variant of social spider optimization using single centroid representation and enhanced mating for data clustering
Source: PeerJ Comput Sci. 2019 Jul 22;5:e201. doi: 10.7717/peerj-cs.201 (PMC7924720; doi:10.7717/peerj-cs.201)
Supplement: Supplemental Information 1 — The raw data used in the experiments. [file peerj-cs-05-201-s001.zip › datasets for peerj/UCI/cancer.docx]

1000025,5,1,1,1,2,1,3,1,1,2

1002945,5,4,4,5,7,10,3,2,1,2

1015425,3,1,1,1,2,2,3,1,1,2

1016277,6,8,8,1,3,4,3,7,1,2

1017023,4,1,1,3,2,1,3,1,1,2

1017122,8,10,10,8,7,10,9,7,1,4

1018099,1,1,1,1,2,10,3,1,1,2

1018561,2,1,2,1,2,1,3,1,1,2

1033078,2,1,1,1,2,1,1,1,5,2

1033078,4,2,1,1,2,1,2,1,1,2

1035283,1,1,1,1,1,1,3,1,1,2

1036172,2,1,1,1,2,1,2,1,1,2

1041801,5,3,3,3,2,3,4,4,1,4

1043999,1,1,1,1,2,3,3,1,1,2

1044572,8,7,5,10,7,9,5,5,4,4

1047630,7,4,6,4,6,1,4,3,1,4

1048672,4,1,1,1,2,1,2,1,1,2

1049815,4,1,1,1,2,1,3,1,1,2

1050670,10,7,7,6,4,10,4,1,2,4

1050718,6,1,1,1,2,1,3,1,1,2

1054590,7,3,2,10,5,10,5,4,4,4

1054593,10,5,5,3,6,7,7,10,1,4

1056784,3,1,1,1,2,1,2,1,1,2

1057013,8,4,5,1,2,?,7,3,1,4

1059552,1,1,1,1,2,1,3,1,1,2

1065726,5,2,3,4,2,7,3,6,1,4

1066373,3,2,1,1,1,1,2,1,1,2

1066979,5,1,1,1,2,1,2,1,1,2

1067444,2,1,1,1,2,1,2,1,1,2

1070935,1,1,3,1,2,1,1,1,1,2

1070935,3,1,1,1,1,1,2,1,1,2

1071760,2,1,1,1,2,1,3,1,1,2

1072179,10,7,7,3,8,5,7,4,3,4

1074610,2,1,1,2,2,1,3,1,1,2

1075123,3,1,2,1,2,1,2,1,1,2

1079304,2,1,1,1,2,1,2,1,1,2

1080185,10,10,10,8,6,1,8,9,1,4

1081791,6,2,1,1,1,1,7,1,1,2

1084584,5,4,4,9,2,10,5,6,1,4

1091262,2,5,3,3,6,7,7,5,1,4

1096800,6,6,6,9,6,?,7,8,1,2

1099510,10,4,3,1,3,3,6,5,2,4

1100524,6,10,10,2,8,10,7,3,3,4

1102573,5,6,5,6,10,1,3,1,1,4

1103608,10,10,10,4,8,1,8,10,1,4

1103722,1,1,1,1,2,1,2,1,2,2

1105257,3,7,7,4,4,9,4,8,1,4

1105524,1,1,1,1,2,1,2,1,1,2

1106095,4,1,1,3,2,1,3,1,1,2

1106829,7,8,7,2,4,8,3,8,2,4

1108370,9,5,8,1,2,3,2,1,5,4

1108449,5,3,3,4,2,4,3,4,1,4

1110102,10,3,6,2,3,5,4,10,2,4

1110503,5,5,5,8,10,8,7,3,7,4

1110524,10,5,5,6,8,8,7,1,1,4

1111249,10,6,6,3,4,5,3,6,1,4

1112209,8,10,10,1,3,6,3,9,1,4

1113038,8,2,4,1,5,1,5,4,4,4

1113483,5,2,3,1,6,10,5,1,1,4

1113906,9,5,5,2,2,2,5,1,1,4

1115282,5,3,5,5,3,3,4,10,1,4

1115293,1,1,1,1,2,2,2,1,1,2

1116116,9,10,10,1,10,8,3,3,1,4

1116132,6,3,4,1,5,2,3,9,1,4

1116192,1,1,1,1,2,1,2,1,1,2

1116998,10,4,2,1,3,2,4,3,10,4

1117152,4,1,1,1,2,1,3,1,1,2

1118039,5,3,4,1,8,10,4,9,1,4

1120559,8,3,8,3,4,9,8,9,8,4

1121732,1,1,1,1,2,1,3,2,1,2

1121919,5,1,3,1,2,1,2,1,1,2

1123061,6,10,2,8,10,2,7,8,10,4

1124651,1,3,3,2,2,1,7,2,1,2

1125035,9,4,5,10,6,10,4,8,1,4

1126417,10,6,4,1,3,4,3,2,3,4

1131294,1,1,2,1,2,2,4,2,1,2

1132347,1,1,4,1,2,1,2,1,1,2

1133041,5,3,1,2,2,1,2,1,1,2

1133136,3,1,1,1,2,3,3,1,1,2

1136142,2,1,1,1,3,1,2,1,1,2

1137156,2,2,2,1,1,1,7,1,1,2

1143978,4,1,1,2,2,1,2,1,1,2

1143978,5,2,1,1,2,1,3,1,1,2

1147044,3,1,1,1,2,2,7,1,1,2

1147699,3,5,7,8,8,9,7,10,7,4

1147748,5,10,6,1,10,4,4,10,10,4

1148278,3,3,6,4,5,8,4,4,1,4

1148873,3,6,6,6,5,10,6,8,3,4

1152331,4,1,1,1,2,1,3,1,1,2

1155546,2,1,1,2,3,1,2,1,1,2

1156272,1,1,1,1,2,1,3,1,1,2

1156948,3,1,1,2,2,1,1,1,1,2

1157734,4,1,1,1,2,1,3,1,1,2

1158247,1,1,1,1,2,1,2,1,1,2

1160476,2,1,1,1,2,1,3,1,1,2

1164066,1,1,1,1,2,1,3,1,1,2

1165297,2,1,1,2,2,1,1,1,1,2

1165790,5,1,1,1,2,1,3,1,1,2

1165926,9,6,9,2,10,6,2,9,10,4

1166630,7,5,6,10,5,10,7,9,4,4

1166654,10,3,5,1,10,5,3,10,2,4

1167439,2,3,4,4,2,5,2,5,1,4

1167471,4,1,2,1,2,1,3,1,1,2

1168359,8,2,3,1,6,3,7,1,1,4

1168736,10,10,10,10,10,1,8,8,8,4

1169049,7,3,4,4,3,3,3,2,7,4

1170419,10,10,10,8,2,10,4,1,1,4

1170420,1,6,8,10,8,10,5,7,1,4

1171710,1,1,1,1,2,1,2,3,1,2

1171710,6,5,4,4,3,9,7,8,3,4

1171795,1,3,1,2,2,2,5,3,2,2

1171845,8,6,4,3,5,9,3,1,1,4

1172152,10,3,3,10,2,10,7,3,3,4

1173216,10,10,10,3,10,8,8,1,1,4

1173235,3,3,2,1,2,3,3,1,1,2

1173347,1,1,1,1,2,5,1,1,1,2

1173347,8,3,3,1,2,2,3,2,1,2

1173509,4,5,5,10,4,10,7,5,8,4

1173514,1,1,1,1,4,3,1,1,1,2

1173681,3,2,1,1,2,2,3,1,1,2

1174057,1,1,2,2,2,1,3,1,1,2

1174057,4,2,1,1,2,2,3,1,1,2

1174131,10,10,10,2,10,10,5,3,3,4

1174428,5,3,5,1,8,10,5,3,1,4

1175937,5,4,6,7,9,7,8,10,1,4

1176406,1,1,1,1,2,1,2,1,1,2

1176881,7,5,3,7,4,10,7,5,5,4

1177027,3,1,1,1,2,1,3,1,1,2

1177399,8,3,5,4,5,10,1,6,2,4

1177512,1,1,1,1,10,1,1,1,1,2

1178580,5,1,3,1,2,1,2,1,1,2

1179818,2,1,1,1,2,1,3,1,1,2

1180194,5,10,8,10,8,10,3,6,3,4

1180523,3,1,1,1,2,1,2,2,1,2

1180831,3,1,1,1,3,1,2,1,1,2

1181356,5,1,1,1,2,2,3,3,1,2

1182404,4,1,1,1,2,1,2,1,1,2

1182410,3,1,1,1,2,1,1,1,1,2

1183240,4,1,2,1,2,1,2,1,1,2

1183246,1,1,1,1,1,?,2,1,1,2

1183516,3,1,1,1,2,1,1,1,1,2

1183911,2,1,1,1,2,1,1,1,1,2

1183983,9,5,5,4,4,5,4,3,3,4

1184184,1,1,1,1,2,5,1,1,1,2

1184241,2,1,1,1,2,1,2,1,1,2

1184840,1,1,3,1,2,?,2,1,1,2

1185609,3,4,5,2,6,8,4,1,1,4

1185610,1,1,1,1,3,2,2,1,1,2

1187457,3,1,1,3,8,1,5,8,1,2

1187805,8,8,7,4,10,10,7,8,7,4

1188472,1,1,1,1,1,1,3,1,1,2

1189266,7,2,4,1,6,10,5,4,3,4

1189286,10,10,8,6,4,5,8,10,1,4

1190394,4,1,1,1,2,3,1,1,1,2

1190485,1,1,1,1,2,1,1,1,1,2

1192325,5,5,5,6,3,10,3,1,1,4

1193091,1,2,2,1,2,1,2,1,1,2

1193210,2,1,1,1,2,1,3,1,1,2

1193683,1,1,2,1,3,?,1,1,1,2

1196295,9,9,10,3,6,10,7,10,6,4

1196915,10,7,7,4,5,10,5,7,2,4

1197080,4,1,1,1,2,1,3,2,1,2

1197270,3,1,1,1,2,1,3,1,1,2

1197440,1,1,1,2,1,3,1,1,7,2

1197510,5,1,1,1,2,?,3,1,1,2

1197979,4,1,1,1,2,2,3,2,1,2

1197993,5,6,7,8,8,10,3,10,3,4

1198128,10,8,10,10,6,1,3,1,10,4

1198641,3,1,1,1,2,1,3,1,1,2

1199219,1,1,1,2,1,1,1,1,1,2

1199731,3,1,1,1,2,1,1,1,1,2

1199983,1,1,1,1,2,1,3,1,1,2

1200772,1,1,1,1,2,1,2,1,1,2

1200847,6,10,10,10,8,10,10,10,7,4

1200892,8,6,5,4,3,10,6,1,1,4

1200952,5,8,7,7,10,10,5,7,1,4

1201834,2,1,1,1,2,1,3,1,1,2

1201936,5,10,10,3,8,1,5,10,3,4

1202125,4,1,1,1,2,1,3,1,1,2

1202812,5,3,3,3,6,10,3,1,1,4

1203096,1,1,1,1,1,1,3,1,1,2

1204242,1,1,1,1,2,1,1,1,1,2

1204898,6,1,1,1,2,1,3,1,1,2

1205138,5,8,8,8,5,10,7,8,1,4

1205579,8,7,6,4,4,10,5,1,1,4

1206089,2,1,1,1,1,1,3,1,1,2

1206695,1,5,8,6,5,8,7,10,1,4

1206841,10,5,6,10,6,10,7,7,10,4

1207986,5,8,4,10,5,8,9,10,1,4

1208301,1,2,3,1,2,1,3,1,1,2

1210963,10,10,10,8,6,8,7,10,1,4

1211202,7,5,10,10,10,10,4,10,3,4

1212232,5,1,1,1,2,1,2,1,1,2

1212251,1,1,1,1,2,1,3,1,1,2

1212422,3,1,1,1,2,1,3,1,1,2

1212422,4,1,1,1,2,1,3,1,1,2

1213375,8,4,4,5,4,7,7,8,2,2

1213383,5,1,1,4,2,1,3,1,1,2

1214092,1,1,1,1,2,1,1,1,1,2

1214556,3,1,1,1,2,1,2,1,1,2

1214966,9,7,7,5,5,10,7,8,3,4

1216694,10,8,8,4,10,10,8,1,1,4

1216947,1,1,1,1,2,1,3,1,1,2

1217051,5,1,1,1,2,1,3,1,1,2

1217264,1,1,1,1,2,1,3,1,1,2

1218105,5,10,10,9,6,10,7,10,5,4

1218741,10,10,9,3,7,5,3,5,1,4

1218860,1,1,1,1,1,1,3,1,1,2

1218860,1,1,1,1,1,1,3,1,1,2

1219406,5,1,1,1,1,1,3,1,1,2

1219525,8,10,10,10,5,10,8,10,6,4

1219859,8,10,8,8,4,8,7,7,1,4

1220330,1,1,1,1,2,1,3,1,1,2

1221863,10,10,10,10,7,10,7,10,4,4

1222047,10,10,10,10,3,10,10,6,1,4

1222936,8,7,8,7,5,5,5,10,2,4

1223282,1,1,1,1,2,1,2,1,1,2

1223426,1,1,1,1,2,1,3,1,1,2

1223793,6,10,7,7,6,4,8,10,2,4

1223967,6,1,3,1,2,1,3,1,1,2

1224329,1,1,1,2,2,1,3,1,1,2

1225799,10,6,4,3,10,10,9,10,1,4

1226012,4,1,1,3,1,5,2,1,1,4

1226612,7,5,6,3,3,8,7,4,1,4

1227210,10,5,5,6,3,10,7,9,2,4

1227244,1,1,1,1,2,1,2,1,1,2

1227481,10,5,7,4,4,10,8,9,1,4

1228152,8,9,9,5,3,5,7,7,1,4

1228311,1,1,1,1,1,1,3,1,1,2

1230175,10,10,10,3,10,10,9,10,1,4

1230688,7,4,7,4,3,7,7,6,1,4

1231387,6,8,7,5,6,8,8,9,2,4

1231706,8,4,6,3,3,1,4,3,1,2

1232225,10,4,5,5,5,10,4,1,1,4

1236043,3,3,2,1,3,1,3,6,1,2

1241232,3,1,4,1,2,?,3,1,1,2

1241559,10,8,8,2,8,10,4,8,10,4

1241679,9,8,8,5,6,2,4,10,4,4

1242364,8,10,10,8,6,9,3,10,10,4

1243256,10,4,3,2,3,10,5,3,2,4

1270479,5,1,3,3,2,2,2,3,1,2

1276091,3,1,1,3,1,1,3,1,1,2

1277018,2,1,1,1,2,1,3,1,1,2

128059,1,1,1,1,2,5,5,1,1,2

1285531,1,1,1,1,2,1,3,1,1,2

1287775,5,1,1,2,2,2,3,1,1,2

144888,8,10,10,8,5,10,7,8,1,4

145447,8,4,4,1,2,9,3,3,1,4

167528,4,1,1,1,2,1,3,6,1,2

169356,3,1,1,1,2,?,3,1,1,2

183913,1,2,2,1,2,1,1,1,1,2

191250,10,4,4,10,2,10,5,3,3,4

1017023,6,3,3,5,3,10,3,5,3,2

1100524,6,10,10,2,8,10,7,3,3,4

1116116,9,10,10,1,10,8,3,3,1,4

1168736,5,6,6,2,4,10,3,6,1,4

1182404,3,1,1,1,2,1,1,1,1,2

1182404,3,1,1,1,2,1,2,1,1,2

1198641,3,1,1,1,2,1,3,1,1,2

242970,5,7,7,1,5,8,3,4,1,2

255644,10,5,8,10,3,10,5,1,3,4

263538,5,10,10,6,10,10,10,6,5,4

274137,8,8,9,4,5,10,7,8,1,4

303213,10,4,4,10,6,10,5,5,1,4

314428,7,9,4,10,10,3,5,3,3,4

1182404,5,1,4,1,2,1,3,2,1,2

1198641,10,10,6,3,3,10,4,3,2,4

320675,3,3,5,2,3,10,7,1,1,4

324427,10,8,8,2,3,4,8,7,8,4

385103,1,1,1,1,2,1,3,1,1,2

390840,8,4,7,1,3,10,3,9,2,4

411453,5,1,1,1,2,1,3,1,1,2

320675,3,3,5,2,3,10,7,1,1,4

428903,7,2,4,1,3,4,3,3,1,4

431495,3,1,1,1,2,1,3,2,1,2

432809,3,1,3,1,2,?,2,1,1,2

434518,3,1,1,1,2,1,2,1,1,2

452264,1,1,1,1,2,1,2,1,1,2

456282,1,1,1,1,2,1,3,1,1,2

476903,10,5,7,3,3,7,3,3,8,4

486283,3,1,1,1,2,1,3,1,1,2

486662,2,1,1,2,2,1,3,1,1,2

488173,1,4,3,10,4,10,5,6,1,4

492268,10,4,6,1,2,10,5,3,1,4

508234,7,4,5,10,2,10,3,8,2,4

527363,8,10,10,10,8,10,10,7,3,4

529329,10,10,10,10,10,10,4,10,10,4

535331,3,1,1,1,3,1,2,1,1,2

543558,6,1,3,1,4,5,5,10,1,4

555977,5,6,6,8,6,10,4,10,4,4

560680,1,1,1,1,2,1,1,1,1,2

561477,1,1,1,1,2,1,3,1,1,2

563649,8,8,8,1,2,?,6,10,1,4

601265,10,4,4,6,2,10,2,3,1,4

606140,1,1,1,1,2,?,2,1,1,2

606722,5,5,7,8,6,10,7,4,1,4

616240,5,3,4,3,4,5,4,7,1,2

61634,5,4,3,1,2,?,2,3,1,2

625201,8,2,1,1,5,1,1,1,1,2

63375,9,1,2,6,4,10,7,7,2,4

635844,8,4,10,5,4,4,7,10,1,4

636130,1,1,1,1,2,1,3,1,1,2

640744,10,10,10,7,9,10,7,10,10,4

646904,1,1,1,1,2,1,3,1,1,2

653777,8,3,4,9,3,10,3,3,1,4

659642,10,8,4,4,4,10,3,10,4,4

666090,1,1,1,1,2,1,3,1,1,2

666942,1,1,1,1,2,1,3,1,1,2

667204,7,8,7,6,4,3,8,8,4,4

673637,3,1,1,1,2,5,5,1,1,2

684955,2,1,1,1,3,1,2,1,1,2

688033,1,1,1,1,2,1,1,1,1,2

691628,8,6,4,10,10,1,3,5,1,4

693702,1,1,1,1,2,1,1,1,1,2

704097,1,1,1,1,1,1,2,1,1,2

704168,4,6,5,6,7,?,4,9,1,2

706426,5,5,5,2,5,10,4,3,1,4

709287,6,8,7,8,6,8,8,9,1,4

718641,1,1,1,1,5,1,3,1,1,2

721482,4,4,4,4,6,5,7,3,1,2

730881,7,6,3,2,5,10,7,4,6,4

733639,3,1,1,1,2,?,3,1,1,2

733639,3,1,1,1,2,1,3,1,1,2

733823,5,4,6,10,2,10,4,1,1,4

740492,1,1,1,1,2,1,3,1,1,2

743348,3,2,2,1,2,1,2,3,1,2

752904,10,1,1,1,2,10,5,4,1,4

756136,1,1,1,1,2,1,2,1,1,2

760001,8,10,3,2,6,4,3,10,1,4

760239,10,4,6,4,5,10,7,1,1,4

76389,10,4,7,2,2,8,6,1,1,4

764974,5,1,1,1,2,1,3,1,2,2

770066,5,2,2,2,2,1,2,2,1,2

785208,5,4,6,6,4,10,4,3,1,4

785615,8,6,7,3,3,10,3,4,2,4

792744,1,1,1,1,2,1,1,1,1,2

797327,6,5,5,8,4,10,3,4,1,4

798429,1,1,1,1,2,1,3,1,1,2

704097,1,1,1,1,1,1,2,1,1,2

806423,8,5,5,5,2,10,4,3,1,4

809912,10,3,3,1,2,10,7,6,1,4

810104,1,1,1,1,2,1,3,1,1,2

814265,2,1,1,1,2,1,1,1,1,2

814911,1,1,1,1,2,1,1,1,1,2

822829,7,6,4,8,10,10,9,5,3,4

826923,1,1,1,1,2,1,1,1,1,2

830690,5,2,2,2,3,1,1,3,1,2

831268,1,1,1,1,1,1,1,3,1,2

832226,3,4,4,10,5,1,3,3,1,4

832567,4,2,3,5,3,8,7,6,1,4

836433,5,1,1,3,2,1,1,1,1,2

837082,2,1,1,1,2,1,3,1,1,2

846832,3,4,5,3,7,3,4,6,1,2

850831,2,7,10,10,7,10,4,9,4,4

855524,1,1,1,1,2,1,2,1,1,2

857774,4,1,1,1,3,1,2,2,1,2

859164,5,3,3,1,3,3,3,3,3,4

859350,8,10,10,7,10,10,7,3,8,4

866325,8,10,5,3,8,4,4,10,3,4

873549,10,3,5,4,3,7,3,5,3,4

877291,6,10,10,10,10,10,8,10,10,4

877943,3,10,3,10,6,10,5,1,4,4

888169,3,2,2,1,4,3,2,1,1,2

888523,4,4,4,2,2,3,2,1,1,2

896404,2,1,1,1,2,1,3,1,1,2

897172,2,1,1,1,2,1,2,1,1,2

95719,6,10,10,10,8,10,7,10,7,4

160296,5,8,8,10,5,10,8,10,3,4

342245,1,1,3,1,2,1,1,1,1,2

428598,1,1,3,1,1,1,2,1,1,2

492561,4,3,2,1,3,1,2,1,1,2

493452,1,1,3,1,2,1,1,1,1,2

493452,4,1,2,1,2,1,2,1,1,2

521441,5,1,1,2,2,1,2,1,1,2

560680,3,1,2,1,2,1,2,1,1,2

636437,1,1,1,1,2,1,1,1,1,2

640712,1,1,1,1,2,1,2,1,1,2

654244,1,1,1,1,1,1,2,1,1,2

657753,3,1,1,4,3,1,2,2,1,2

685977,5,3,4,1,4,1,3,1,1,2

805448,1,1,1,1,2,1,1,1,1,2

846423,10,6,3,6,4,10,7,8,4,4

1002504,3,2,2,2,2,1,3,2,1,2

1022257,2,1,1,1,2,1,1,1,1,2

1026122,2,1,1,1,2,1,1,1,1,2

1071084,3,3,2,2,3,1,1,2,3,2

1080233,7,6,6,3,2,10,7,1,1,4

1114570,5,3,3,2,3,1,3,1,1,2

1114570,2,1,1,1,2,1,2,2,1,2

1116715,5,1,1,1,3,2,2,2,1,2

1131411,1,1,1,2,2,1,2,1,1,2

1151734,10,8,7,4,3,10,7,9,1,4

1156017,3,1,1,1,2,1,2,1,1,2

1158247,1,1,1,1,1,1,1,1,1,2

1158405,1,2,3,1,2,1,2,1,1,2

1168278,3,1,1,1,2,1,2,1,1,2

1176187,3,1,1,1,2,1,3,1,1,2

1196263,4,1,1,1,2,1,1,1,1,2

1196475,3,2,1,1,2,1,2,2,1,2

1206314,1,2,3,1,2,1,1,1,1,2

1211265,3,10,8,7,6,9,9,3,8,4

1213784,3,1,1,1,2,1,1,1,1,2

1223003,5,3,3,1,2,1,2,1,1,2

1223306,3,1,1,1,2,4,1,1,1,2

1223543,1,2,1,3,2,1,1,2,1,2

1229929,1,1,1,1,2,1,2,1,1,2

1231853,4,2,2,1,2,1,2,1,1,2

1234554,1,1,1,1,2,1,2,1,1,2

1236837,2,3,2,2,2,2,3,1,1,2

1237674,3,1,2,1,2,1,2,1,1,2

1238021,1,1,1,1,2,1,2,1,1,2

1238464,1,1,1,1,1,?,2,1,1,2

1238633,10,10,10,6,8,4,8,5,1,4

1238915,5,1,2,1,2,1,3,1,1,2

1238948,8,5,6,2,3,10,6,6,1,4

1239232,3,3,2,6,3,3,3,5,1,2

1239347,8,7,8,5,10,10,7,2,1,4

1239967,1,1,1,1,2,1,2,1,1,2

1240337,5,2,2,2,2,2,3,2,2,2

1253505,2,3,1,1,5,1,1,1,1,2

1255384,3,2,2,3,2,3,3,1,1,2

1257200,10,10,10,7,10,10,8,2,1,4

1257648,4,3,3,1,2,1,3,3,1,2

1257815,5,1,3,1,2,1,2,1,1,2

1257938,3,1,1,1,2,1,1,1,1,2

1258549,9,10,10,10,10,10,10,10,1,4

1258556,5,3,6,1,2,1,1,1,1,2

1266154,8,7,8,2,4,2,5,10,1,4

1272039,1,1,1,1,2,1,2,1,1,2

1276091,2,1,1,1,2,1,2,1,1,2

1276091,1,3,1,1,2,1,2,2,1,2

1276091,5,1,1,3,4,1,3,2,1,2

1277629,5,1,1,1,2,1,2,2,1,2

1293439,3,2,2,3,2,1,1,1,1,2

1293439,6,9,7,5,5,8,4,2,1,2

1294562,10,8,10,1,3,10,5,1,1,4

1295186,10,10,10,1,6,1,2,8,1,4

527337,4,1,1,1,2,1,1,1,1,2

558538,4,1,3,3,2,1,1,1,1,2

566509,5,1,1,1,2,1,1,1,1,2

608157,10,4,3,10,4,10,10,1,1,4

677910,5,2,2,4,2,4,1,1,1,2

734111,1,1,1,3,2,3,1,1,1,2

734111,1,1,1,1,2,2,1,1,1,2

780555,5,1,1,6,3,1,2,1,1,2

827627,2,1,1,1,2,1,1,1,1,2

1049837,1,1,1,1,2,1,1,1,1,2

1058849,5,1,1,1,2,1,1,1,1,2

1182404,1,1,1,1,1,1,1,1,1,2

1193544,5,7,9,8,6,10,8,10,1,4

1201870,4,1,1,3,1,1,2,1,1,2

1202253,5,1,1,1,2,1,1,1,1,2

1227081,3,1,1,3,2,1,1,1,1,2

1230994,4,5,5,8,6,10,10,7,1,4

1238410,2,3,1,1,3,1,1,1,1,2

1246562,10,2,2,1,2,6,1,1,2,4

1257470,10,6,5,8,5,10,8,6,1,4

1259008,8,8,9,6,6,3,10,10,1,4

1266124,5,1,2,1,2,1,1,1,1,2

1267898,5,1,3,1,2,1,1,1,1,2

1268313,5,1,1,3,2,1,1,1,1,2

1268804,3,1,1,1,2,5,1,1,1,2

1276091,6,1,1,3,2,1,1,1,1,2

1280258,4,1,1,1,2,1,1,2,1,2

1293966,4,1,1,1,2,1,1,1,1,2

1296572,10,9,8,7,6,4,7,10,3,4

1298416,10,6,6,2,4,10,9,7,1,4

1299596,6,6,6,5,4,10,7,6,2,4

1105524,4,1,1,1,2,1,1,1,1,2

1181685,1,1,2,1,2,1,2,1,1,2

1211594,3,1,1,1,1,1,2,1,1,2

1238777,6,1,1,3,2,1,1,1,1,2

1257608,6,1,1,1,1,1,1,1,1,2

1269574,4,1,1,1,2,1,1,1,1,2

1277145,5,1,1,1,2,1,1,1,1,2

1287282,3,1,1,1,2,1,1,1,1,2

1296025,4,1,2,1,2,1,1,1,1,2

1296263,4,1,1,1,2,1,1,1,1,2

1296593,5,2,1,1,2,1,1,1,1,2

1299161,4,8,7,10,4,10,7,5,1,4

1301945,5,1,1,1,1,1,1,1,1,2

1302428,5,3,2,4,2,1,1,1,1,2

1318169,9,10,10,10,10,5,10,10,10,4

474162,8,7,8,5,5,10,9,10,1,4

787451,5,1,2,1,2,1,1,1,1,2

1002025,1,1,1,3,1,3,1,1,1,2

1070522,3,1,1,1,1,1,2,1,1,2

1073960,10,10,10,10,6,10,8,1,5,4

1076352,3,6,4,10,3,3,3,4,1,4

1084139,6,3,2,1,3,4,4,1,1,4

1115293,1,1,1,1,2,1,1,1,1,2

1119189,5,8,9,4,3,10,7,1,1,4

1133991,4,1,1,1,1,1,2,1,1,2

1142706,5,10,10,10,6,10,6,5,2,4

1155967,5,1,2,10,4,5,2,1,1,2

1170945,3,1,1,1,1,1,2,1,1,2

1181567,1,1,1,1,1,1,1,1,1,2

1182404,4,2,1,1,2,1,1,1,1,2

1204558,4,1,1,1,2,1,2,1,1,2

1217952,4,1,1,1,2,1,2,1,1,2

1224565,6,1,1,1,2,1,3,1,1,2

1238186,4,1,1,1,2,1,2,1,1,2

1253917,4,1,1,2,2,1,2,1,1,2

1265899,4,1,1,1,2,1,3,1,1,2

1268766,1,1,1,1,2,1,1,1,1,2

1277268,3,3,1,1,2,1,1,1,1,2

1286943,8,10,10,10,7,5,4,8,7,4

1295508,1,1,1,1,2,4,1,1,1,2

1297327,5,1,1,1,2,1,1,1,1,2

1297522,2,1,1,1,2,1,1,1,1,2

1298360,1,1,1,1,2,1,1,1,1,2

1299924,5,1,1,1,2,1,2,1,1,2

1299994,5,1,1,1,2,1,1,1,1,2

1304595,3,1,1,1,1,1,2,1,1,2

1306282,6,6,7,10,3,10,8,10,2,4

1313325,4,10,4,7,3,10,9,10,1,4

1320077,1,1,1,1,1,1,1,1,1,2

1320077,1,1,1,1,1,1,2,1,1,2

1320304,3,1,2,2,2,1,1,1,1,2

1330439,4,7,8,3,4,10,9,1,1,4

333093,1,1,1,1,3,1,1,1,1,2

369565,4,1,1,1,3,1,1,1,1,2

412300,10,4,5,4,3,5,7,3,1,4

672113,7,5,6,10,4,10,5,3,1,4

749653,3,1,1,1,2,1,2,1,1,2

769612,3,1,1,2,2,1,1,1,1,2

769612,4,1,1,1,2,1,1,1,1,2

798429,4,1,1,1,2,1,3,1,1,2

807657,6,1,3,2,2,1,1,1,1,2

8233704,4,1,1,1,1,1,2,1,1,2

837480,7,4,4,3,4,10,6,9,1,4

867392,4,2,2,1,2,1,2,1,1,2

869828,1,1,1,1,1,1,3,1,1,2

1043068,3,1,1,1,2,1,2,1,1,2

1056171,2,1,1,1,2,1,2,1,1,2

1061990,1,1,3,2,2,1,3,1,1,2

1113061,5,1,1,1,2,1,3,1,1,2

1116192,5,1,2,1,2,1,3,1,1,2

1135090,4,1,1,1,2,1,2,1,1,2

1145420,6,1,1,1,2,1,2,1,1,2

1158157,5,1,1,1,2,2,2,1,1,2

1171578,3,1,1,1,2,1,1,1,1,2

1174841,5,3,1,1,2,1,1,1,1,2

1184586,4,1,1,1,2,1,2,1,1,2

1186936,2,1,3,2,2,1,2,1,1,2

1197527,5,1,1,1,2,1,2,1,1,2

1222464,6,10,10,10,4,10,7,10,1,4

1240603,2,1,1,1,1,1,1,1,1,2

1240603,3,1,1,1,1,1,1,1,1,2

1241035,7,8,3,7,4,5,7,8,2,4

1287971,3,1,1,1,2,1,2,1,1,2

1289391,1,1,1,1,2,1,3,1,1,2

1299924,3,2,2,2,2,1,4,2,1,2

1306339,4,4,2,1,2,5,2,1,2,2

1313658,3,1,1,1,2,1,1,1,1,2

1313982,4,3,1,1,2,1,4,8,1,2

1321264,5,2,2,2,1,1,2,1,1,2

1321321,5,1,1,3,2,1,1,1,1,2

1321348,2,1,1,1,2,1,2,1,1,2

1321931,5,1,1,1,2,1,2,1,1,2

1321942,5,1,1,1,2,1,3,1,1,2

1321942,5,1,1,1,2,1,3,1,1,2

1328331,1,1,1,1,2,1,3,1,1,2

1328755,3,1,1,1,2,1,2,1,1,2

1331405,4,1,1,1,2,1,3,2,1,2

1331412,5,7,10,10,5,10,10,10,1,4

1333104,3,1,2,1,2,1,3,1,1,2

1334071,4,1,1,1,2,3,2,1,1,2

1343068,8,4,4,1,6,10,2,5,2,4

1343374,10,10,8,10,6,5,10,3,1,4

1344121,8,10,4,4,8,10,8,2,1,4

142932,7,6,10,5,3,10,9,10,2,4

183936,3,1,1,1,2,1,2,1,1,2

324382,1,1,1,1,2,1,2,1,1,2

378275,10,9,7,3,4,2,7,7,1,4

385103,5,1,2,1,2,1,3,1,1,2

690557,5,1,1,1,2,1,2,1,1,2

695091,1,1,1,1,2,1,2,1,1,2

695219,1,1,1,1,2,1,2,1,1,2

824249,1,1,1,1,2,1,3,1,1,2

871549,5,1,2,1,2,1,2,1,1,2

878358,5,7,10,6,5,10,7,5,1,4

1107684,6,10,5,5,4,10,6,10,1,4

1115762,3,1,1,1,2,1,1,1,1,2

1217717,5,1,1,6,3,1,1,1,1,2

1239420,1,1,1,1,2,1,1,1,1,2

1254538,8,10,10,10,6,10,10,10,1,4

1261751,5,1,1,1,2,1,2,2,1,2

1268275,9,8,8,9,6,3,4,1,1,4

1272166,5,1,1,1,2,1,1,1,1,2

1294261,4,10,8,5,4,1,10,1,1,4

1295529,2,5,7,6,4,10,7,6,1,4

1298484,10,3,4,5,3,10,4,1,1,4

1311875,5,1,2,1,2,1,1,1,1,2

1315506,4,8,6,3,4,10,7,1,1,4

1320141,5,1,1,1,2,1,2,1,1,2

1325309,4,1,2,1,2,1,2,1,1,2

1333063,5,1,3,1,2,1,3,1,1,2

1333495,3,1,1,1,2,1,2,1,1,2

1334659,5,2,4,1,1,1,1,1,1,2

1336798,3,1,1,1,2,1,2,1,1,2

1344449,1,1,1,1,1,1,2,1,1,2

1350568,4,1,1,1,2,1,2,1,1,2

1352663,5,4,6,8,4,1,8,10,1,4

188336,5,3,2,8,5,10,8,1,2,4

352431,10,5,10,3,5,8,7,8,3,4

353098,4,1,1,2,2,1,1,1,1,2

411453,1,1,1,1,2,1,1,1,1,2

557583,5,10,10,10,10,10,10,1,1,4

636375,5,1,1,1,2,1,1,1,1,2

736150,10,4,3,10,3,10,7,1,2,4

803531,5,10,10,10,5,2,8,5,1,4

822829,8,10,10,10,6,10,10,10,10,4

1016634,2,3,1,1,2,1,2,1,1,2

1031608,2,1,1,1,1,1,2,1,1,2

1041043,4,1,3,1,2,1,2,1,1,2

1042252,3,1,1,1,2,1,2,1,1,2

1057067,1,1,1,1,1,?,1,1,1,2

1061990,4,1,1,1,2,1,2,1,1,2

1073836,5,1,1,1,2,1,2,1,1,2

1083817,3,1,1,1,2,1,2,1,1,2

1096352,6,3,3,3,3,2,6,1,1,2

1140597,7,1,2,3,2,1,2,1,1,2

1149548,1,1,1,1,2,1,1,1,1,2

1174009,5,1,1,2,1,1,2,1,1,2

1183596,3,1,3,1,3,4,1,1,1,2

1190386,4,6,6,5,7,6,7,7,3,4

1190546,2,1,1,1,2,5,1,1,1,2

1213273,2,1,1,1,2,1,1,1,1,2

1218982,4,1,1,1,2,1,1,1,1,2

1225382,6,2,3,1,2,1,1,1,1,2

1235807,5,1,1,1,2,1,2,1,1,2

1238777,1,1,1,1,2,1,1,1,1,2

1253955,8,7,4,4,5,3,5,10,1,4

1257366,3,1,1,1,2,1,1,1,1,2

1260659,3,1,4,1,2,1,1,1,1,2

1268952,10,10,7,8,7,1,10,10,3,4

1275807,4,2,4,3,2,2,2,1,1,2

1277792,4,1,1,1,2,1,1,1,1,2

1277792,5,1,1,3,2,1,1,1,1,2

1285722,4,1,1,3,2,1,1,1,1,2

1288608,3,1,1,1,2,1,2,1,1,2

1290203,3,1,1,1,2,1,2,1,1,2

1294413,1,1,1,1,2,1,1,1,1,2

1299596,2,1,1,1,2,1,1,1,1,2

1303489,3,1,1,1,2,1,2,1,1,2

1311033,1,2,2,1,2,1,1,1,1,2

1311108,1,1,1,3,2,1,1,1,1,2

1315807,5,10,10,10,10,2,10,10,10,4

1318671,3,1,1,1,2,1,2,1,1,2

1319609,3,1,1,2,3,4,1,1,1,2

1323477,1,2,1,3,2,1,2,1,1,2

1324572,5,1,1,1,2,1,2,2,1,2

1324681,4,1,1,1,2,1,2,1,1,2

1325159,3,1,1,1,2,1,3,1,1,2

1326892,3,1,1,1,2,1,2,1,1,2

1330361,5,1,1,1,2,1,2,1,1,2

1333877,5,4,5,1,8,1,3,6,1,2

1334015,7,8,8,7,3,10,7,2,3,4

1334667,1,1,1,1,2,1,1,1,1,2

1339781,1,1,1,1,2,1,2,1,1,2

1339781,4,1,1,1,2,1,3,1,1,2

13454352,1,1,3,1,2,1,2,1,1,2

1345452,1,1,3,1,2,1,2,1,1,2

1345593,3,1,1,3,2,1,2,1,1,2

1347749,1,1,1,1,2,1,1,1,1,2

1347943,5,2,2,2,2,1,1,1,2,2

1348851,3,1,1,1,2,1,3,1,1,2

1350319,5,7,4,1,6,1,7,10,3,4

1350423,5,10,10,8,5,5,7,10,1,4

1352848,3,10,7,8,5,8,7,4,1,4

1353092,3,2,1,2,2,1,3,1,1,2

1354840,2,1,1,1,2,1,3,1,1,2

1354840,5,3,2,1,3,1,1,1,1,2

1355260,1,1,1,1,2,1,2,1,1,2

1365075,4,1,4,1,2,1,1,1,1,2

1365328,1,1,2,1,2,1,2,1,1,2

1368267,5,1,1,1,2,1,1,1,1,2

1368273,1,1,1,1,2,1,1,1,1,2

1368882,2,1,1,1,2,1,1,1,1,2

1369821,10,10,10,10,5,10,10,10,7,4

1371026,5,10,10,10,4,10,5,6,3,4

1371920,5,1,1,1,2,1,3,2,1,2

466906,1,1,1,1,2,1,1,1,1,2

466906,1,1,1,1,2,1,1,1,1,2

534555,1,1,1,1,2,1,1,1,1,2

536708,1,1,1,1,2,1,1,1,1,2

566346,3,1,1,1,2,1,2,3,1,2

603148,4,1,1,1,2,1,1,1,1,2

654546,1,1,1,1,2,1,1,1,8,2

654546,1,1,1,3,2,1,1,1,1,2

695091,5,10,10,5,4,5,4,4,1,4

714039,3,1,1,1,2,1,1,1,1,2

763235,3,1,1,1,2,1,2,1,2,2

776715,3,1,1,1,3,2,1,1,1,2

841769,2,1,1,1,2,1,1,1,1,2

888820,5,10,10,3,7,3,8,10,2,4

897471,4,8,6,4,3,4,10,6,1,4

897471,4,8,8,5,4,5,10,4,1,4
