# Supplementary material for: A novel variant of social spider optimization using single centroid representation and enhanced mating for data clustering
Source: PeerJ Comput Sci. 2019 Jul 22;5:e201. doi: 10.7717/peerj-cs.201 (PMC7924720; doi:10.7717/peerj-cs.201)
Supplement: Supplemental Information 1 — The raw data used in the experiments. [file peerj-cs-05-201-s001.zip › datasets for peerj/UCI/glass.pdf]

```
1,14.23,1.71,2.43,15.6,127,2.8,3.06,.28,2.29,5.64,1.04,3.92,1065
1,13.2,1.78,2.14,11.2,100,2.65,2.76,.26,1.28,4.38,1.05,3.4,1050
1,13.16,2.36,2.67,18.6,101,2.8,3.24,.3,2.81,5.68,1.03,3.17,1185
1,14.37,1.95,2.5,16.8,113,3.85,3.49,.24,2.18,7.8,.86,3.45,1480
1,13.24,2.59,2.87,21,118,2.8,2.69,.39,1.82,4.32,1.04,2.93,735
1,14.2,1.76,2.45,15.2,112,3.27,3.39,.34,1.97,6.75,1.05,2.85,1450
1,14.39,1.87,2.45,14.6,96,2.5,2.52,.3,1.98,5.25,1.02,3.58,1290
1,14.06,2.15,2.61,17.6,121,2.6,2.51,.31,1.25,5.05,1.06,3.58,1295
1,14.83,1.64,2.17,14,97,2.8,2.98,.29,1.98,5.2,1.08,2.85,1045
1,13.86,1.35,2.27,16,98,2.98,3.15,.22,1.85,7.22,1.01,3.55,1045
1,14.1,2.16,2.3,18,105,2.95,3.32,.22,2.38,5.75,1.25,3.17,1510
1,14.12,1.48,2.32,16.8,95,2.2,2.43,.26,1.57,5,1.17,2.82,1280
1,13.75,1.73,2.41,16,89,2.6,2.76,.29,1.81,5.6,1.15,2.9,1320
1,14.75,1.73,2.39,11.4,91,3.1,3.69,.43,2.81,5.4,1.25,2.73,1150
1,14.38,1.87,2.38,12,102,3.3,3.64,.29,2.96,7.5,1.2,3,1547
1,13.63,1.81,2.7,17.2,112,2.85,2.91,.3,1.46,7.3,1.28,2.88,1310
1,14.3,1.92,2.72,20,120,2.8,3.14,.33,1.97,6.2,1.07,2.65,1280
1,13.83,1.57,2.62,20,115,2.95,3.4,.4,1.72,6.6,1.13,2.57,1130
1,14.19,1.59,2.48,16.5,108,3.3,3.93,.32,1.86,8.7,1.23,2.82,1680
1,13.64,3.1,2.56,15.2,116,2.7,3.03,.17,1.66,5.1,.96,3.36,845
1,14.06,1.63,2.28,16,126,3,3.17,.24,2.1,5.65,1.09,3.71,780
1,12.93,3.8,2.65,18.6,102,2.41,2.41,.25,1.98,4.5,1.03,3.52,770
1,13.71,1.86,2.36,16.6,101,2.61,2.88,.27,1.69,3.8,1.11,4,1035
1,12.85,1.6,2.52,17.8,95,2.48,2.37,.26,1.46,3.93,1.09,3.63,1015
1,13.5,1.81,2.61,20,96,2.53,2.61,.28,1.66,3.52,1.12,3.82,845
1,13.05,2.05,3.22,25,124,2.63,2.68,.47,1.92,3.58,1.13,3.2,830
1,13.39,1.77,2.62,16.1,93,2.85,2.94,.34,1.45,4.8,.92,3.22,1195
1,13.3,1.72,2.14,17,94,2.4,2.19,.27,1.35,3.95,1.02,2.77,1285
1,13.87,1.9,2.8,19.4,107,2.95,2.97,.37,1.76,4.5,1.25,3.4,915
1,14.02,1.68,2.21,16,96,2.65,2.33,.26,1.98,4.7,1.04,3.59,1035
1,13.73,1.5,2.7,22.5,101,3,3.25,.29,2.38,5.7,1.19,2.71,1285
1,13.58,1.66,2.36,19.1,106,2.86,3.19,.22,1.95,6.9,1.09,2.88,1515
1,13.68,1.83,2.36,17.2,104,2.42,2.69,.42,1.97,3.84,1.23,2.87,990
1,13.76,1.53,2.7,19.5,132,2.95,2.74,.5,1.35,5.4,1.25,3,1235
1,13.51,1.8,2.65,19,110,2.35,2.53,.29,1.54,4.2,1.1,2.87,1095
1,13.48,1.81,2.41,20.5,100,2.7,2.98,.26,1.86,5.1,1.04,3.47,920
1,13.28,1.64,2.84,15.5,110,2.6,2.68,.34,1.36,4.6,1.09,2.78,880
1,13.05,1.65,2.55,18,98,2.45,2.43,.29,1.44,4.25,1.12,2.51,1105
1,13.07,1.5,2.1,15.5,98,2.4,2.64,.28,1.37,3.7,1.18,2.69,1020
1,14.22,3.99,2.51,13.2,128,3,3.04,.2,2.08,5.1,.89,3.53,760
1,13.56,1.71,2.31,16.2,117,3.15,3.29,.34,2.34,6.13,.95,3.38,795
1,13.41,3.84,2.12,18.8,90,2.45,2.68,.27,1.48,4.28,.91,3,1035
1,13.88,1.89,2.59,15,101,3.25,3.56,.17,1.7,5.43,.88,3.56,1095
1,13.24,3.98,2.29,17.5,103,2.64,2.63,.32,1.66,4.36,.82,3,680
1,13.05,1.77,2.1,17,107,3,3,.28,2.03,5.04,.88,3.35,885
1,14.21,4.04,2.44,18.9,111,2.85,2.65,.3,1.25,5.24,.87,3.33,1080
1,14.38,3.59,2.28,16,102,3.25,3.17,.27,2.19,4.9,1.04,3.44,1065
1,13.9,1.68,2.12,16,101,3.1,3.39,.21,2.14,6.1,.91,3.33,985
1,14.1,2.02,2.4,18.8,103,2.75,2.92,.32,2.38,6.2,1.07,2.75,1060
1,13.94,1.73,2.27,17.4,108,2.88,3.54,.32,2.08,8.90,1.12,3.1,1260
1,13.05,1.73,2.04,12.4,92,2.72,3.27,.17,2.91,7.2,1.12,2.91,1150
1,13.83,1.65,2.6,17.2,94,2.45,2.99,.22,2.29,5.6,1.24,3.37,1265
1,13.82,1.75,2.42,14,111,3.88,3.74,.32,1.87,7.05,1.01,3.26,1190
1,13.77,1.9,2.68,17.1,115,3,2.79,.39,1.68,6.3,1.13,2.93,1375
1,13.74,1.67,2.25,16.4,118,2.6,2.9,.21,1.62,5.85,.92,3.2,1060
1,13.56,1.73,2.46,20.5,116,2.96,2.78,.2,2.45,6.25,.98,3.03,1120
1,14.22,1.7,2.3,16.3,118,3.2,3,.26,2.03,6.38,.94,3.31,970
1,13.29,1.97,2.68,16.8,102,3,3.23,.31,1.66,6,1.07,2.84,1270
1,13.72,1.43,2.5,16.7,108,3.4,3.67,.19,2.04,6.8,.89,2.87,1285
2,12.37,.94,1.36,10.6,88,1.98,.57,.28,.42,1.95,1.05,1.82,520
2,12.33,1.1,2.28,16,101,2.05,1.09,.63,.41,3.27,1.25,1.67,680
2,12.64,1.36,2.02,16.8,100,2.02,1.41,.53,.62,5.75,.98,1.59,450
2,13.67,1.25,1.92,18,94,2.1,1.79,.32,.73,3.8,1.23,2.46,630
2,12.37,1.13,2.16,19,87,3.5,3.1,.19,1.87,4.45,1.22,2.87,420
```

2,12.17,1.45,2.53,19,104,1.89,1.75,.45,1.03,2.95,1.45,2.23,355
2,12.37,1.21,2.56,18.1,98,2.42,2.65,.37,2.08,4.6,1.19,2.3,678
2,13.11,1.01,1.7,15,78,2.98,3.18,.26,2.28,5.3,1.12,3.18,502
2,12.37,1.17,1.92,19.6,78,2.11,2,.27,1.04,4.68,1.12,3.48,510
2,13.34,.94,2.36,17,110,2.53,1.3,.55,.42,3.17,1.02,1.93,750
2,12.21,1.19,1.75,16.8,151,1.85,1.28,.14,2.5,2.85,1.28,3.07,718
2,12.29,1.61,2.21,20.4,103,1.1,1.02,.37,1.46,3.05,.906,1.82,870
2,13.86,1.51,2.67,25,86,2.95,2.86,.21,1.87,3.38,1.36,3.16,410
2,13.49,1.66,2.24,24,87,1.88,1.84,.27,1.03,3.74,.98,2.78,472
2,12.99,1.67,2.6,30,139,3.3,2.89,.21,1.96,3.35,1.31,3.5,985
2,11.96,1.09,2.3,21,101,3.38,2.14,.13,1.65,3.21,.99,3.13,886
2,11.66,1.88,1.92,16,97,1.61,1.57,.34,1.15,3.8,1.23,2.14,428
2,13.03,.9,1.71,16,86,1.95,2.03,.24,1.46,4.6,1.19,2.48,392
2,11.84,2.89,2.23,18,112,1.72,1.32,.43,.95,2.65,.96,2.52,500
2,12.33,.99,1.95,14.8,136,1.9,1.85,.35,2.76,3.4,1.06,2.31,750
2,12.7,3.87,2.4,23,101,2.83,2.55,.43,1.95,2.57,1.19,3.13,463
2,12,.92,2,19,86,2.42,2.26,.3,1.43,2.5,1.38,3.12,278
2,12.72,1.81,2.2,18.8,86,2.2,2.53,.26,1.77,3.9,1.16,3.14,714
2,12.08,1.13,2.51,24,78,2,1.58,.4,1.4,2.2,1.31,2.72,630
2,13.05,3.86,2.32,22.5,85,1.65,1.59,.61,1.62,4.8,.84,2.01,515
2,11.84,.89,2.58,18,94,2.2,2.21,.22,2.35,3.05,.79,3.08,520
2,12.67,.98,2.24,18,99,2.2,1.94,.3,1.46,2.62,1.23,3.16,450
2,12.16,1.61,2.31,22.8,90,1.78,1.69,.43,1.56,2.45,1.33,2.26,495
2,11.65,1.67,2.62,26,88,1.92,1.61,.4,1.34,2.6,1.36,3.21,562
2,11.64,2.06,2.46,21.6,84,1.95,1.69,.48,1.35,2.8,1,2.75,680
2,12.08,1.33,2.3,23.6,70,2.2,1.59,.42,1.38,1.74,1.07,3.21,625
2,12.08,1.83,2.32,18.5,81,1.6,1.5,.52,1.64,2.4,1.08,2.27,480
2,12,1.51,2.42,22,86,1.45,1.25,.5,1.63,3.6,1.05,2.65,450
2,12.69,1.53,2.26,20.7,80,1.38,1.46,.58,1.62,3.05,.96,2.06,495
2,12.29,2.83,2.22,18,88,2.45,2.25,.25,1.99,2.15,1.15,3.3,290
2,11.62,1.99,2.28,18,98,3.02,2.26,.17,1.35,3.25,1.16,2.96,345
2,12.47,1.52,2.2,19,162,2.5,2.27,.32,3.28,2.6,1.16,2.63,937
2,11.81,2.12,2.74,21.5,134,1.6,.99,.14,1.56,2.5,.95,2.26,625
2,12.29,1.41,1.98,16,85,2.55,2.5,.29,1.77,2.9,1.23,2.74,428
2,12.37,1.07,2.1,18.5,88,3.52,3.75,.24,1.95,4.5,1.04,2.77,660
2,12.29,3.17,2.21,18,88,2.85,2.99,.45,2.81,2.3,1.42,2.83,406
2,12.08,2.08,1.7,17.5,97,2.23,2.17,.26,1.4,3.3,1.27,2.96,710
2,12.6,1.34,1.9,18.5,88,1.45,1.36,.29,1.35,2.45,1.04,2.77,562
2,12.34,2.45,2.46,21,98,2.56,2.11,.34,1.31,2.8,.8,3.38,438
2,11.82,1.72,1.88,19.5,86,2.5,1.64,.37,1.42,2.06,.94,2.44,415
2,12.51,1.73,1.98,20.5,85,2.2,1.92,.32,1.48,2.94,1.04,3.57,672
2,12.42,2.55,2.27,22,90,1.68,1.84,.66,1.42,2.7,.86,3.3,315
2,12.25,1.73,2.12,19,80,1.65,2.03,.37,1.63,3.4,1,3.17,510
2,12.72,1.75,2.28,22.5,84,1.38,1.76,.48,1.63,3.3,.88,2.42,488
2,12.22,1.29,1.94,19,92,2.36,2.04,.39,2.08,2.7,.86,3.02,312
2,11.61,1.35,2.7,20,94,2.74,2.92,.29,2.49,2.65,.96,3.26,680
2,11.46,3.74,1.82,19.5,107,3.18,2.58,.24,3.58,2.9,.75,2.81,562
2,12.52,2.43,2.17,21,88,2.55,2.27,.26,1.22,2,.9,2.78,325
2,11.76,2.68,2.92,20,103,1.75,2.03,.6,1.05,3.8,1.23,2.5,607
2,11.41,.74,2.5,21,88,2.48,2.01,.42,1.44,3.08,1.1,2.31,434
2,12.08,1.39,2.5,22.5,84,2.56,2.29,.43,1.04,2.9,.93,3.19,385
2,11.03,1.51,2.2,21.5,85,2.46,2.17,.52,2.01,1.9,1.71,2.87,407
2,11.82,1.47,1.99,20.8,86,1.98,1.6,.3,1.53,1.95,.95,3.33,495
2,12.42,1.61,2.19,22.5,108,2,2.09,.34,1.61,2.06,1.06,2.96,345
2,12.77,3.43,1.98,16,80,1.63,1.25,.43,.83,3.4,.7,2.12,372
2,12,3.43,2,19,87,2,1.64,.37,1.87,1.28,.93,3.05,564
2,11.45,2.4,2.42,20,96,2.9,2.79,.32,1.83,3.25,.8,3.39,625
2,11.56,2.05,3.23,28.5,119,3.18,5.08,.47,1.87,6,.93,3.69,465
2,12.42,4.43,2.73,26.5,102,2.2,2.13,.43,1.71,2.08,.92,3.12,365
2,13.05,5.8,2.13,21.5,86,2.62,2.65,.3,2.01,2.6,.73,3.1,380
2,11.87,4.31,2.39,21,82,2.86,3.03,.21,2.91,2.8,.75,3.64,380
2,12.07,2.16,2.17,21,85,2.6,2.65,.37,1.35,2.76,.86,3.28,378
2,12.43,1.53,2.29,21.5,86,2.74,3.15,.39,1.77,3.94,.69,2.84,352
2,11.79,2.13,2.78,28.5,92,2.13,2.24,.58,1.76,3,.97,2.44,466
2,12.37,1.63,2.3,24.5,88,2.22,2.45,.4,1.9,2.12,.89,2.78,342

```
2,12.04,4.3,2.38,22,80,2.1,1.75,.42,1.35,2.6,.79,2.57,580
3,12.86,1.35,2.32,18,122,1.51,1.25,.21,.94,4.1,.76,1.29,630
3,12.88,2.99,2.4,20,104,1.3,1.22,.24,.83,5.4,.74,1.42,530
3,12.81,2.31,2.4,24,98,1.15,1.09,.27,.83,5.7,.66,1.36,560
3,12.7,3.55,2.36,21.5,106,1.7,1.2,.17,.84,5,.78,1.29,600
3,12.51,1.24,2.25,17.5,85,2,.58,.6,1.25,5.45,.75,1.51,650
3,12.6,2.46,2.2,18.5,94,1.62,.66,.63,.94,7.1,.73,1.58,695
3,12.25,4.72,2.54,21,89,1.38,.47,.53,.8,3.85,.75,1.27,720
3,12.53,5.51,2.64,25,96,1.79,.6,.63,1.1,5,.82,1.69,515
3,13.49,3.59,2.19,19.5,88,1.62,.48,.58,.88,5.7,.81,1.82,580
3,12.84,2.96,2.61,24,101,2.32,.6,.53,.81,4.92,.89,2.15,590
3,12.93,2.81,2.7,21,96,1.54,.5,.53,.75,4.6,.77,2.31,600
3,13.36,2.56,2.35,20,89,1.4,.5,.37,.64,5.6,.7,2.47,780
3,13.52,3.17,2.72,23.5,97,1.55,.52,.5,.55,4.35,.89,2.06,520
3,13.62,4.95,2.35,20,92,2,.8,.47,1.02,4.4,.91,2.05,550
3,12.25,3.88,2.2,18.5,112,1.38,.78,.29,1.14,8.21,.65,2,855
3,13.16,3.57,2.15,21,102,1.5,.55,.43,1.3,4,.6,1.68,830
3,13.88,5.04,2.23,20,80,.98,.34,.4,.68,4.9,.58,1.33,415
3,12.87,4.61,2.48,21.5,86,1.7,.65,.47,.86,7.65,.54,1.86,625
3,13.32,3.24,2.38,21.5,92,1.93,.76,.45,1.25,8.42,.55,1.62,650
3,13.08,3.9,2.36,21.5,113,1.41,1.39,.34,1.14,9.40,.57,1.33,550
3,13.5,3.12,2.62,24,123,1.4,1.57,.22,1.25,8.60,.59,1.3,500
3,12.79,2.67,2.48,22,112,1.48,1.36,.24,1.26,10.8,.48,1.47,480
3,13.11,1.9,2.75,25.5,116,2.2,1.28,.26,1.56,7.1,.61,1.33,425
3,13.23,3.3,2.28,18.5,98,1.8,.83,.61,1.87,10.52,.56,1.51,675
3,12.58,1.29,2.1,20,103,1.48,.58,.53,1.4,7.6,.58,1.55,640
3,13.17,5.19,2.32,22,93,1.74,.63,.61,1.55,7.9,.6,1.48,725
3,13.84,4.12,2.38,19.5,89,1.8,.83,.48,1.56,9.01,.57,1.64,480
3,12.45,3.03,2.64,27,97,1.9,.58,.63,1.14,7.5,.67,1.73,880
3,14.34,1.68,2.7,25,98,2.8,1.31,.53,2.7,13,.57,1.96,660
3,13.48,1.67,2.64,22.5,89,2.6,1.1,.52,2.29,11.75,.57,1.78,620
3,12.36,3.83,2.38,21,88,2.3,.92,.5,1.04,7.65,.56,1.58,520
3,13.69,3.26,2.54,20,107,1.83,.56,.5,.8,5.88,.96,1.82,680
3,12.85,3.27,2.58,22,106,1.65,.6,.6,.96,5.58,.87,2.11,570
3,12.96,3.45,2.35,18.5,106,1.39,.7,.4,.94,5.28,.68,1.75,675
3,13.78,2.76,2.3,22,90,1.35,.68,.41,1.03,9.58,.7,1.68,615
3,13.73,4.36,2.26,22.5,88,1.28,.47,.52,1.15,6.62,.78,1.75,520
3,13.45,3.7,2.6,23,111,1.7,.92,.43,1.46,10.68,.85,1.56,695
3,12.82,3.37,2.3,19.5,88,1.48,.66,.4,.97,10.26,.72,1.75,685
3,13.58,2.58,2.69,24.5,105,1.55,.84,.39,1.54,8.66,.74,1.8,750
3,13.4,4.6,2.86,25,112,1.98,.96,.27,1.11,8.5,.67,1.92,630
3,12.2,3.03,2.32,19,96,1.25,.49,.4,.73,5.5,.66,1.83,510
3,12.77,2.39,2.28,19.5,86,1.39,.51,.48,.64,9.899999,.57,1.63,470
3,14.16,2.51,2.48,20,91,1.68,.7,.44,1.24,9.7,.62,1.71,660
3,13.71,5.65,2.45,20.5,95,1.68,.61,.52,1.06,7.7,.64,1.74,740
3,13.4,3.91,2.48,23,102,1.8,.75,.43,1.41,7.3,.7,1.56,750
3,13.27,4.28,2.26,20,120,1.59,.69,.43,1.35,10.2,.59,1.56,835
3,13.17,2.59,2.37,20,120,1.65,.68,.53,1.46,9.3,.6,1.62,840
3,14.13,4.1,2.74,24.5,96,2.05,.76,.56,1.35,9.2,.61,1.6,560
```
