# Supplementary material for: A novel variant of social spider optimization using single centroid representation and enhanced mating for data clustering
Source: PeerJ Comput Sci. 2019 Jul 22;5:e201. doi: 10.7717/peerj-cs.201 (PMC7924720; doi:10.7717/peerj-cs.201)
Supplement: Supplemental Information 1 — The raw data used in the experiments. [file peerj-cs-05-201-s001.zip › datasets for peerj/UCI/Iris.pdf]

```
5.1,3.5,1.4,0.2,Iris-setosa
4.9,3.0,1.4,0.2,Iris-setosa
4.7,3.2,1.3,0.2,Iris-setosa
4.6,3.1,1.5,0.2,Iris-setosa
5.0,3.6,1.4,0.2,Iris-setosa
5.4,3.9,1.7,0.4,Iris-setosa
4.6,3.4,1.4,0.3,Iris-setosa
5.0,3.4,1.5,0.2,Iris-setosa
4.4,2.9,1.4,0.2,Iris-setosa
4.9,3.1,1.5,0.1,Iris-setosa
5.4,3.7,1.5,0.2,Iris-setosa
4.8,3.4,1.6,0.2,Iris-setosa
4.8,3.0,1.4,0.1,Iris-setosa
4.3,3.0,1.1,0.1,Iris-setosa
5.8,4.0,1.2,0.2,Iris-setosa
5.7,4.4,1.5,0.4,Iris-setosa
5.4,3.9,1.3,0.4,Iris-setosa
5.1,3.5,1.4,0.3,Iris-setosa
5.7,3.8,1.7,0.3,Iris-setosa
5.1,3.8,1.5,0.3,Iris-setosa
5.4,3.4,1.7,0.2,Iris-setosa
5.1,3.7,1.5,0.4,Iris-setosa
4.6,3.6,1.0,0.2,Iris-setosa
5.1,3.3,1.7,0.5,Iris-setosa
4.8,3.4,1.9,0.2,Iris-setosa
5.0,3.0,1.6,0.2,Iris-setosa
5.0,3.4,1.6,0.4,Iris-setosa
5.2,3.5,1.5,0.2,Iris-setosa
5.2,3.4,1.4,0.2,Iris-setosa
4.7,3.2,1.6,0.2,Iris-setosa
4.8,3.1,1.6,0.2,Iris-setosa
5.4,3.4,1.5,0.4,Iris-setosa
5.2,4.1,1.5,0.1,Iris-setosa
5.5,4.2,1.4,0.2,Iris-setosa
4.9,3.1,1.5,0.1,Iris-setosa
5.0,3.2,1.2,0.2,Iris-setosa
5.5,3.5,1.3,0.2,Iris-setosa
4.9,3.1,1.5,0.1,Iris-setosa
4.4,3.0,1.3,0.2,Iris-setosa
5.1,3.4,1.5,0.2,Iris-setosa
5.0,3.5,1.3,0.3,Iris-setosa
4.5,2.3,1.3,0.3,Iris-setosa
4.4,3.2,1.3,0.2,Iris-setosa
5.0,3.5,1.6,0.6,Iris-setosa
5.1,3.8,1.9,0.4,Iris-setosa
4.8,3.0,1.4,0.3,Iris-setosa
5.1,3.8,1.6,0.2,Iris-setosa
4.6,3.2,1.4,0.2,Iris-setosa
5.3,3.7,1.5,0.2,Iris-setosa
5.0,3.3,1.4,0.2,Iris-setosa
7.0,3.2,4.7,1.4,Iris-versicolor
6.4,3.2,4.5,1.5,Iris-versicolor
6.9,3.1,4.9,1.5,Iris-versicolor
5.5,2.3,4.0,1.3,Iris-versicolor
6.5,2.8,4.6,1.5,Iris-versicolor
5.7,2.8,4.5,1.3,Iris-versicolor
6.3,3.3,4.7,1.6,Iris-versicolor
4.9,2.4,3.3,1.0,Iris-versicolor
6.6,2.9,4.6,1.3,Iris-versicolor
5.2,2.7,3.9,1.4,Iris-versicolor
5.0,2.0,3.5,1.0,Iris-versicolor
5.9,3.0,4.2,1.5,Iris-versicolor
6.0,2.2,4.0,1.0,Iris-versicolor
6.1,2.9,4.7,1.4,Iris-versicolor
```

```
5.6,2.9,3.6,1.3,Iris-versicolor
6.7,3.1,4.4,1.4,Iris-versicolor
5.6,3.0,4.5,1.5,Iris-versicolor
5.8,2.7,4.1,1.0,Iris-versicolor
6.2,2.2,4.5,1.5,Iris-versicolor
5.6,2.5,3.9,1.1,Iris-versicolor
5.9,3.2,4.8,1.8,Iris-versicolor
6.1,2.8,4.0,1.3,Iris-versicolor
6.3,2.5,4.9,1.5,Iris-versicolor
6.1,2.8,4.7,1.2,Iris-versicolor
6.4,2.9,4.3,1.3,Iris-versicolor
6.6,3.0,4.4,1.4,Iris-versicolor
6.8,2.8,4.8,1.4,Iris-versicolor
6.7,3.0,5.0,1.7,Iris-versicolor
6.0,2.9,4.5,1.5,Iris-versicolor
5.7,2.6,3.5,1.0,Iris-versicolor
5.5,2.4,3.8,1.1,Iris-versicolor
5.5,2.4,3.7,1.0,Iris-versicolor
5.8,2.7,3.9,1.2,Iris-versicolor
6.0,2.7,5.1,1.6,Iris-versicolor
5.4,3.0,4.5,1.5,Iris-versicolor
6.0,3.4,4.5,1.6,Iris-versicolor
6.7,3.1,4.7,1.5,Iris-versicolor
6.3,2.3,4.4,1.3,Iris-versicolor
5.6,3.0,4.1,1.3,Iris-versicolor
5.5,2.5,4.0,1.3,Iris-versicolor
5.5,2.6,4.4,1.2,Iris-versicolor
6.1,3.0,4.6,1.4,Iris-versicolor
5.8,2.6,4.0,1.2,Iris-versicolor
5.0,2.3,3.3,1.0,Iris-versicolor
5.6,2.7,4.2,1.3,Iris-versicolor
5.7,3.0,4.2,1.2,Iris-versicolor
5.7,2.9,4.2,1.3,Iris-versicolor
6.2,2.9,4.3,1.3,Iris-versicolor
5.1,2.5,3.0,1.1,Iris-versicolor
5.7,2.8,4.1,1.3,Iris-versicolor
6.3,3.3,6.0,2.5,Iris-virginica
5.8,2.7,5.1,1.9,Iris-virginica
7.1,3.0,5.9,2.1,Iris-virginica
6.3,2.9,5.6,1.8,Iris-virginica
6.5,3.0,5.8,2.2,Iris-virginica
7.6,3.0,6.6,2.1,Iris-virginica
4.9,2.5,4.5,1.7,Iris-virginica
7.3,2.9,6.3,1.8,Iris-virginica
6.7,2.5,5.8,1.8,Iris-virginica
7.2,3.6,6.1,2.5,Iris-virginica
6.5,3.2,5.1,2.0,Iris-virginica
6.4,2.7,5.3,1.9,Iris-virginica
6.8,3.0,5.5,2.1,Iris-virginica
5.7,2.5,5.0,2.0,Iris-virginica
5.8,2.8,5.1,2.4,Iris-virginica
6.4,3.2,5.3,2.3,Iris-virginica
6.5,3.0,5.5,1.8,Iris-virginica
7.7,3.8,6.7,2.2,Iris-virginica
7.7,2.6,6.9,2.3,Iris-virginica
6.0,2.2,5.0,1.5,Iris-virginica
6.9,3.2,5.7,2.3,Iris-virginica
5.6,2.8,4.9,2.0,Iris-virginica
7.7,2.8,6.7,2.0,Iris-virginica
6.3,2.7,4.9,1.8,Iris-virginica
6.7,3.3,5.7,2.1,Iris-virginica
7.2,3.2,6.0,1.8,Iris-virginica
6.2,2.8,4.8,1.8,Iris-virginica
6.1,3.0,4.9,1.8,Iris-virginica
6.4,2.8,5.6,2.1,Iris-virginica
```

```
7.2,3.0,5.8,1.6,Iris-virginica
7.4,2.8,6.1,1.9,Iris-virginica
7.9,3.8,6.4,2.0,Iris-virginica
6.4,2.8,5.6,2.2,Iris-virginica
6.3,2.8,5.1,1.5,Iris-virginica
6.1,2.6,5.6,1.4,Iris-virginica
7.7,3.0,6.1,2.3,Iris-virginica
6.3,3.4,5.6,2.4,Iris-virginica
6.4,3.1,5.5,1.8,Iris-virginica
6.0,3.0,4.8,1.8,Iris-virginica
6.9,3.1,5.4,2.1,Iris-virginica
6.7,3.1,5.6,2.4,Iris-virginica
6.9,3.1,5.1,2.3,Iris-virginica
5.8,2.7,5.1,1.9,Iris-virginica
6.8,3.2,5.9,2.3,Iris-virginica
6.7,3.3,5.7,2.5,Iris-virginica
6.7,3.0,5.2,2.3,Iris-virginica
6.3,2.5,5.0,1.9,Iris-virginica
6.5,3.0,5.2,2.0,Iris-virginica
6.2,3.4,5.4,2.3,Iris-virginica
5.9,3.0,5.1,1.8,Iris-virginica
```
