# Supplementary material for: A novel variant of social spider optimization using single centroid representation and enhanced mating for data clustering
Source: PeerJ Comput Sci. 2019 Jul 22;5:e201. doi: 10.7717/peerj-cs.201 (PMC7924720; doi:10.7717/peerj-cs.201)
Supplement: Supplemental Information 2 — SSODCSC was written in Java. To run it, only JDK is required.The data files should be stored with .txt extension in current working directory. The program works for both text datasets and attribute based datasets. [file peerj-cs-05-201-s002.pdf]

```java
import java.util.*;
import java.io.*;
import java.util.Random;
import java.util.HashSet;
import java.util.Random;
public class ssodcsc
{
static int k=1;
static int n=50;
static double pf=0.7;
static int maxiter=400;
  static int globalmin=100000000;
static double[][] f=new double[k][100];
double avgweight1=0.0;
double avgweight2=0.0;

static int k1=6;


static String[][] cclass=
{
    {
"glass1.txt","glass2.txt","glass3.txt","glass4.txt","glass5.txt","glass6.
txt","glass7.txt","glass8.txt","glass9.txt","glass10.txt",
"glass11.txt","glass12.txt","glass13.txt","glass14.txt","glass15.txt","gl
ass16.txt","glass17.txt","glass18.txt","glass19.txt","glass20.txt",
"glass21.txt","glass22.txt","glass23.txt","glass24.txt","glass25.txt","gl
ass26.txt","glass27.txt","glass28.txt","glass29.txt","glass30.txt",
"glass31.txt","glass32.txt","glass33.txt","glass34.txt","glass35.txt","gl
ass36.txt","glass37.txt","glass38.txt","glass39.txt","glass40.txt",
"glass41.txt","glass42.txt","glass43.txt","glass44.txt","glass45.txt","gl
ass46.txt","glass47.txt","glass48.txt","glass49.txt","glass50.txt",
"glass51.txt",
"glass52.txt","glass53.txt","glass54.txt","glass55.txt","glass56.txt","gl
ass57.txt","glass58.txt","glass59.txt",
"glass60.txt","glass61.txt","glass62.txt","glass63.txt","glass64.txt","gl
ass65.txt","glass66.txt","glass67.txt","glass68.txt","glass69.txt",
"glass70.txt"
    },

{
"glass71.txt","glass72.txt","glass73.txt","glass74.txt","glass75.txt","gl
ass76.txt","glass77.txt","glass78.txt","glass79.txt","glass80.txt",
"glass81.txt","glass82.txt","glass83.txt","glass84.txt","glass85.txt","gl
ass86.txt","glass87.txt","glass88.txt","glass89.txt","glass90.txt",
"glass91.txt","glass92.txt","glass93.txt","glass94.txt","glass95.txt","gl
ass96.txt","glass97.txt","glass98.txt","glass99.txt","glass100.txt",
"glass101.txt","glass102.txt","glass103.txt","glass104.txt","glass105.txt
","glass106.txt","glass107.txt","glass108.txt","glass109.txt",
"glass110.txt","glass111.txt","glass112.txt","glass113.txt","glass114.txt
","glass115.txt","glass116.txt","glass117.txt","glass118.txt",
"glass119.txt","glass120.txt", "glass121.txt",
"glass122.txt","glass123.txt","glass124.txt","glass125.txt","glass126.txt
","glass127.txt",
```

```
"glass128.txt","glass129.txt","glass130.txt","glass131.txt","glass132.txt
","glass133.txt","glass134.txt","glass135.txt","glass136.txt",
"glass137.txt","glass138.txt","glass139.txt",
"glass140.txt","glass141.txt","glass142.txt","glass143.txt","glass144.txt
","glass145.txt",
"glass146.txt"
    },
{
"glass147.txt","glass148.txt","glass149.txt","glass150.txt","glass151.txt
","glass152.txt","glass153.txt","glass154.txt","glass155.txt","glass156.t
xt",
"glass157.txt","glass158.txt","glass159.txt","glass160.txt","glass161.txt
","glass162.txt","glass163.txt"
},
{
"glass164.txt","glass165.txt","glass166.txt",
"glass167.txt","glass168.txt","glass169.txt","glass170.txt","glass171.txt
","glass172.txt","glass173.txt","glass174.txt","glass175.txt","glass176.t
xt"
},
{
"glass177.txt","glass178.txt","glass179.txt","glass180.txt","glass181.txt
","glass182.txt","glass183.txt","glass184.txt","glass185.txt"
},

{
"glass186.txt","glass187.txt","glass188.txt","glass189.txt","glass190.txt
","glass191.txt","glass192.txt","glass193.txt","glass194.txt",
"glass195.txt","glass196.txt","glass197.txt","glass198.txt","glass199.txt
","glass200.txt","glass201.txt","glass202.txt","glass203.txt",
"glass204.txt","glass205.txt","glass206.txt","glass207.txt","glass208.txt
","glass209.txt","glass210.txt","glass211.txt","glass212.txt",
"glass213.txt","glass214.txt"
}
};

static String[][] cluster= new String[200][200];


         static  ArrayList<Map<double[], TreeSet<Integer>>> sp;
         static  ArrayList<Map<double[], TreeSet<Integer>>> spresult;

         static ArrayList<Integer> dmalpos;
         static ArrayList<Integer> dfmalpos;
      static Map<double[], TreeSet<Integer>> best1;
        static Map<double[], TreeSet<Integer>> best3;

 static Map<double[], TreeSet<Integer>> best2;
        static ArrayList<String> global = new ArrayList<String>();

        static int nf= (int) (n * (0.90- Math.random()*0.35));
        static  int nm=n-nf;
        static  double[] fitness;
```

```java
                        static  double[] weight;
                        static   double[] vib;
                        static   int gbest;
                        static  int lbest;
                        static int fbest;
                        static int cf=0;
                        static int ct=0;
 static ArrayList<double[]> vecspace = new ArrayList<double[]>();



                   static    int index=-1;

                    static Map<double[], TreeSet<Integer>> temp1;
                              static  Map<double[], TreeSet<Integer>>
temp2;

                           static   Map<double[], TreeSet<Integer>>
temp3;



    public static void main(String[]args) throws
IOException,InterruptedException

    {
             long starttime = System.nanoTime();

       //read in documents from all .txt files in same folder as sso.java
      //save parallel lists of documents (String[]) and their filenames,
and create global set list of words
        ArrayList<String[]> docs = new ArrayList<String[]>();
        ArrayList<String> filenames = new ArrayList<String>();
        //int maxiter=10;


        File folder = new File(".");
         // no.of. txt files
         List<File> files = Arrays.asList(folder.listFiles(new
FileFilter() {
             public boolean accept(File f) {
                 return f.isFile() && f.getName().endsWith(".txt");
             }
         }));
         BufferedReader in = null;
         for(File f:files){
           in = new BufferedReader(new FileReader(f));

           StringBuffer sb = new StringBuffer();
           String s = null;
           while((s = in.readLine()) != null){
                 sb.append(s);
```

```
            }
            //input cleaning regex
            String[] d =
sb.toString().replaceAll("[\\W&&[^\\s]]","").split("\\W+");
            for(String u:d)
                  if(!global.contains(u))
                                {
                        global.add(u);
                                ct++;
                                }
            docs.add(d);
            filenames.add(f.getName());
                cf++;
          }

             System.out.println("terms="+ ct + "filess="+ cf);


          //

            //compute tf-idf and create document vectors (double[])


          for(String[] s:docs){
            double[] d = new double[global.size()];
            for(int i=0;i<global.size();i++)
                  d[i] = tf(s,global.get(i)) * idf(docs,global.get(i));
            vecspace.add(d);


          }


                  for(double[] obj:vecspace)
                   {
                       for(int i=0;i<global.size();i++)
                        System.out.print(obj[i] + "    ");
                       System.out.println();
                  }


System.out.println("************************** vector space
representation of text files  is
over*******************************************");


    //ArrayList<Map<double[], TreeSet<Integer>>> sp = new
ArrayList<Map<double[], TreeSet<Integer>>>();

     for(int ss=1;ss<=maxiter;ss++)
    {
            sp = new ArrayList<Map<double[], TreeSet<Integer>>>();
             dmalpos = new ArrayList<Integer>();
          dfmalpos = new ArrayList<Integer>();
```

```
        fitness = new double[n];
          weight = new double[n];
          vib = new double[3];



        for(int j=1;j<= n;j++)
        {

                   if (j<=nf)
                   System.out.println("initialization of  female spider"
+ j);
                   else
                   System.out.println("initialization of  male spider" +
(j-nf));
                   HashMap<double[],TreeSet<Integer>> clusters = new
HashMap<double[],TreeSet<Integer>>();
                 HashMap<double[],TreeSet<Integer>> step = new
HashMap<double[],TreeSet<Integer>>();
                 HashSet<Integer> rand = new HashSet<Integer>();
         // TreeMap<Double,HashMap<double[],TreeSet<Integer>>> errorsums
= new TreeMap<Double,HashMap<double[],TreeSet<Integer>>>();
        // no.of clusters in each spider


            clusters.clear();
            step.clear();
            rand.clear();
            //randomly initialize cluster centers
                while(rand.size()< k)
                  rand.add((int)(Math.random()*vecspace.size()));
                for(int r:rand){
                  double[] temp = new double[vecspace.get(r).length];
                  System.arraycopy(vecspace.get(r),0,temp,0,temp.length);
                  step.put(temp,new TreeSet<Integer>());
               }




              for(Map.Entry m:step.entrySet()){
                                                  double[] c= (double[])
m.getKey();
                                                  for(int
i=0;i<global.size();i++)

System.out.print(c[i] + "   ");


System.out.println(m.getValue());



                                                  }
```

```
            sp.add(step);

       }
           System.out.println("           intialization of spiders is over
");

System.out.println("*************************************************
*************************************************************
****************");



     for(int iter=1; iter<= maxiter;iter++)
     {



          // step 1     Assigning each document to nearest cluster
centroid in each spider



                       for(int i=0; i<= n-1;i++)
                       {





                              // double d=0.0;



                               for(int j=0;j<=cf-1;j++)

                               {
                                            double[] dmincent = new
double[global.size()];

                                            double[] obj=
vecspace.get(j);
                                            Map<double[],
TreeSet<Integer>> s= sp.get(i);

                                            Collection c = s.keySet();


                                            Iterator itr = c.iterator();
                                               double  dmin=1000000;
```

```
ArrayList<double[]>  cc= new
ArrayList<double[]>();
  while(itr.hasNext())
  {

      double[] cent=
(double[]) itr.next();
       cc.add(cent);
      double sum=0.0;

        for(int
p=0;p<global.size();p++)
           sum += (obj[p] -
cent[p])  * (obj[p] - cent[p]);
      double d=
Math.sqrt(sum);


      if(d <= dmin)
      {
            dmin=d;

            dmincent=cent;
       }
  }

   if(s.get(dmincent)!=null)
   {
          TreeSet<Integer>
t= s.get(dmincent);
            t.add(j);

s.put(dmincent,t);

   }

   else
   {
           TreeSet<Integer>
t= new TreeSet<Integer>();
            t.add(j);
```

```
s.put(dmincent,t);
                                                }




                                     sp.set(i,s);

                              }


                        // System.out.println(sp.get(i));
                        }




            //  step2     caculate fitness of each spider

                           cal_fitness();



         //    step3    calculate weight of each spider

                               cal_weight();

                          int ii;
                          localmin=100000000;
                       for(ii=0;ii<=n-1;ii++)
                       {
                           if( fitness[ii]<localmin)
                           {
                                localmin=fitness[ii];
                                index=ii;
                                best1 =sp.get(index);

                           }
                       }
            //        System.out.println("local best in iteration" +
iter + best1);



                       if( globalmin>localmin)
                        {
                          globalmin=localmin;
                          gindex=index;
                          best2=sp.get(gindex);
                        }
```

```
                    if(iter==maxiter)
                     break;

            //     step4    calculate next positions of female spiders

                          female_next_pos();

            //    step5     calculate next positions of male spiders

                       male_next_pos();


             //   step6   perform mating operation

                         mating();



             }





              }


           for(int i=0; i<=k1-1;i++)
            {

            Map<double[], TreeSet<Integer>> s = spresult.get(i);
                     Collection c = s.keySet();

                      Iterator itr = c.iterator();

                        int i=0;
                       int[] cs =new int[10];

                       while(itr.hasNext())
                       {

                              System.out.println("cluster " + (i+1) +
"contains");

                              double[] cent= (double[]) itr.next();

                              TreeSet<Integer> t = s.get(cent);

                              Iterator<Integer> it = t.iterator();
                              int j2=0;
                             while(it.hasNext())
                            {
                                 Integer j= it.next();
                                 //System.out.println(vecspace.get(j));
                                 cluster[i][j2]=filenames.get(j);
```

```
                              System.out.println(cluster[i][j2]);
                              j2++;
                         }
                         cs[i]=j2;

                        i++;
                        System.out.println();
                    }

              }



                  long estimatedtime= System.nanoTime()-starttime;
                  System.out.println("elapsed time =" + estimatedtime);

             double fmeasure=0;

             for(int m=0;m<=k-1;m++)
             {
                       for(int j=0;j<=k-1;j++)
                       {
                                  double a1 =0;
                                  for(int
ni=0;i<cluster[j].length;ni++)
                                 {
                                          for(int
ii=0;ii<cclass[m].length;ii++)
                                          {


                                               if
((cclass[m][ii]!=null) &&(cluster[j][ni]!=null) &&
(cclass[m][ii].equals(cluster[j][ni])))
                                               {
                                                   a1++;
                                                   break;
                                               }
                                          }
                                  }



                                 double  b1= cclass[m].length-a1;
                                  double c1= cluster[j].length-a1;
                                 double  d1= cf-(a1+c1);
                                 double  recall=a1/(a1+b1);
                                 double prec=a1/(a1+c1);

                                 if((recall+prec)!=0)

                                 f[m][j] =
(2*recall*prec)/(recall+prec);
```

```
                                  else
                                  f[m][j]=0;

                        }



                        double max = f[m][0];
                        for(int jj=1;jj<=k-1;jj++)
                        {
                              if(f[m][jj]>max)
                              max=f[m][jj];
                        }


                    fmeasure +=((double)cclass[m].length/cf) *max;
          }




          System.out.println("F-measure of our clustering algorithm " +
fmeasure);


   double accuracy=0;
           double max =0;

            for(int m=0;m<=k-1;m++)
            {          max =0;
                      for(int j=0;j<=k-1;j++)
                      {
                                   double a1 =0;
                                   for(int
ni=0;ni<cluster[j].length;ni++)
                                  {
                                          for(int
ii=0;ii<cclass[m].length;ii++)
                                          {



                                               if
((cclass[m][ii]!=null) &&(cluster[j][ni]!=null) &&
(cclass[m][ii].equals(cluster[j][ni])))
                                               {
                                                   a1++;
                                                   break;
                                               }
                                          }
                                   }



                                  double  b1= 50-a1;
```

```
                                double c1= cs[j]-a1;
                               double  d1= 150-(a1 +b1+c1);
                               if ( (a1 + d1)/(a1+b1+c1+d1) >max)
                               max =(a1 + d1)/(a1+b1+c1+d1);


                      }






                   accuracy += max;
          }


          System.out.println("Accuracy =" +accuracy/k1);


     }

       static void find_cos_sim( Map<double[], TreeSet<Integer>> best3)
       {

                    double avg=0;

                     Map<double[], TreeSet<Integer>> s = best3;
                     Collection c = s.keySet();

                      Iterator itr = c.iterator();

                        int i=0;
                        double[] sim= new double[k];

                       while(itr.hasNext())
                       {
                               sim[i]=0;

                               double[] cent= (double[]) itr.next();

                               TreeSet<Integer> t = s.get(cent);

                               Iterator<Integer> it = t.iterator();

                              while(it.hasNext())
                             {
                                  Integer j= it.next();
                                  double[] cent1=vecspace.get(j);

                                  TreeSet<Integer> t1 = s.get(cent);

                                     Iterator<Integer> it1 =
t1.iterator();
```

```
                                    while(it1.hasNext())
                                    {
                                         Integer j1= it1.next();
                                         double[]
cent2=vecspace.get(j1);
                                          sim[i] += cosSim(cent1,cent2);
                                     }
                               }
                               sim[i]  /=(t.size()*t.size());

                               i++;
                        }


                        for(int ii=0;ii<=k-1;ii++)
                        {
                             avg +=sim[ii];
                        }
                        avg=avg/k1;



                    System.out.println("average cosine similarity= " + avg);




               }
```

```
     static double cosSim(double[] a, double[] b){
      double dotp=0, maga=0, magb=0;
      for(int i=0;i<a.length;i++){
           dotp+=a[i]*b[i];
           maga+=Math.pow(a[i],2);
           magb+=Math.pow(b[i],2);
      }
```

```
      maga = Math.sqrt(maga);
      magb = Math.sqrt(magb);
      double d = dotp / (maga * magb);
      return d==Double.NaN?0:d;
    }

 static void cal_fitness()
{

         for( int i=0;i<=n-1;i++)
         {
                double[] sum = new double[k];
                Map<double[], TreeSet<Integer>> s1=sp.get(i);

                 Iterator itr1 = s1.keySet().iterator();
                 fitness[i]=0;

                int  nc=0;
                 for(;itr1.hasNext();)
                 {

                          double[] cent1= (double[]) itr1.next();
                          sum[nc]=0;



                         TreeSet<Integer> ts=  s1.get(cent1);

                         Iterator<Integer> itr = ts.iterator();

                             while(itr.hasNext())
                            {
                                 Integer j= itr.next();


                                 double[] cent2  = vecspace.get(j);
                                 double d=0.0;
                                 for(int p=0;p<=global.size()-1;p++)
                                 d += (cent1[p]-cent2[p]) * (cent1[p]-
cent2[p]);

                                 d=Math.sqrt(d);
                                 sum[nc] +=d;
                              }

                              if (ts.size()!=0)
                              sum[nc] /=ts.size();

                        nc++;

                       if(nc>k1-1)
                       break;
               }

              for(int j=0;j<=k1-1;j++)
```

```
              fitness[i] +=sum[j];

           // System.out.println(" fitness  of   spider " + (i+1) + " is "
+ fitness[i]);

    }
}



  static  void cal_weight()

  {
                 //
System.out.println("*************************************************
*************************************************************************
***");
                 //  System.out.println("                 weights  of
spiders                    ");

              double  minfitness=100000;
              double  maxfitness=0.0;
              for(int i=0;i<=n-1;i++)
              {
                     if (fitness[i] < minfitness)
                       minfitness=fitness[i];
                     if (fitness[i] > maxfitness)
                       maxfitness=fitness[i];
              }

              for(int i=0;i<=n-1;i++)
              {
                  weight[i]=    (  fitness[i] - minfitness) / (
maxfitness - minfitness);
               //   System.out.println(" weight  of   spider " + (i+1) +
" is " + weight[i]);

               }
}




  static double tf(String[] doc, String term){
      double n = 0;
      for(String s:doc)
            if(s.equalsIgnoreCase(term))
                 n++;
      return n/doc.length;
    }
```

```
    static double idf(ArrayList<String[]> docs, String term){
      double n = 0;
      for(String[] x:docs)
           for(String s:x)
                 if(s.equalsIgnoreCase(term)){
                       n++;
                       break;
                 }
      return Math.log(docs.size()/n);
    }


    static  void  vibper( int  i)
    {
        double bestweight=1;

        for(int j=0;j<=n-1;j++)
        {
                 if (weight[j] < bestweight)
                 {
                          bestweight=weight[j];
                          gbest=j;
                 }
        }

        vib[0]= weight[gbest] *  Math.exp( -
Math.pow(distance(sp.get(gbest), sp.get(i)),2));
      // System.out.println("  intensity of vibration from global best
spider for spider " + (i+1) +"    " +  vib[0]);


         double mind=0;
        for(int j=0;j<=n-1;j++)
        {         double d=distance(sp.get(i),sp.get(j));
                 if( d < mind)
                 mind= d;
        }



        for(int j=0;j<=n-1;j++)
        {
                 if (weight[j] < weight[i] &&
distance(sp.get(i),sp.get(j))== mind)
                 {

                          lbest=j;
                 }
        }

         vib[1]= weight[lbest] *  Math.exp( -
Math.pow(distance(sp.get(lbest), sp.get(i)),2));
        // System.out.println("  intensity of vibration from local best
spider for spider " + (i+1) +"    " +  vib[1]);
```

```
          mind=0;
        for(int j=0;j<=nf-1;j++)
        {         double d=distance(sp.get(i),sp.get(j));
                  if( d < mind)
                  mind= d;
        }



        for(int j=0;j<=nf-1;j++)
        {
                  if (weight[j] < weight[i] &&
distance(sp.get(i),sp.get(j))== mind)
                  {

                            fbest=j;
                  }
        }

         vib[2]= weight[fbest] *  Math.exp( -
Math.pow(distance(sp.get(fbest), sp.get(i)),2));
      //  System.out.println("  intensity of vibration from nearer female
spider for spider " + (i+1) +"    " +  vib[2]);


   }



    static double  distance(Map<double[], TreeSet<Integer>> s1,
Map<double[], TreeSet<Integer>> s2)
    {


                                                Iterator itr1 =
s1.keySet().iterator();
                                                Iterator itr2 =
s2.keySet().iterator();




                                                  System.out.println();

                                                  double sum=0.0;





for(;itr1.hasNext()&&itr2.hasNext();)
                                                       {
```

```
                                                            double[] cent1=
(double[]) itr1.next();

                                                            double[] cent2=
(double[]) itr2.next();


System.out.println();
                                                             for(int
p=0;p<global.size();p++)
                                                                    sum +=
(cent1[p] - cent2[p])  * (cent1[p] - cent2[p]);
                                                      }



                                                      return Math.sqrt(sum);

 }



  static Map<double[], TreeSet<Integer>> add(Map<double[],
TreeSet<Integer>> s1, Map<double[], TreeSet<Integer>> s2)
  {
                            HashMap<double[], TreeSet<Integer>> temp =
new HashMap<double[], TreeSet<Integer>>();



                                 Iterator itr1 = s1.keySet().iterator();
                                 Iterator itr2 = s2.keySet().iterator();



for(;itr1.hasNext()&&itr2.hasNext();)
                                     {


                                                            double[] cent1=
(double[]) itr1.next();

                                                            double[] cent2=
(double[]) itr2.next();

                                                            double[]  cent3=
new double[global.size()];

                                                               for(int
p=0;p<global.size();p++)
                                                                {

cent3[p]= cent1[p] + cent2[p];

                                                                }
```

```
temp.put(cent3,new TreeSet<Integer>());
                                       }

                                      return temp;


}




   static Map<double[], TreeSet<Integer>> minus(Map<double[],
TreeSet<Integer>> s1, Map<double[], TreeSet<Integer>> s2)
  {
     HashMap<double[], TreeSet<Integer>> temp = new HashMap<double[],
TreeSet<Integer>>();



                                 Iterator itr1 = s1.keySet().iterator();
                                 Iterator itr2 = s2.keySet().iterator();



for(;itr1.hasNext()&&itr2.hasNext();)
                                      {


                                                         double[] cent1=
(double[]) itr1.next();

                                                         double[] cent2=
(double[]) itr2.next();

                                                         double[]  cent3=
new double[global.size()];

                                                            for(int
p=0;p<global.size();p++)
                                                             {

cent3[p]= cent1[p] - cent2[p];
                                                             }



temp.put(cent3,new TreeSet<Integer>());
                                       }

                                      return temp;
```

```
  }

  static Map<double[], TreeSet<Integer>> addr(Map<double[],
TreeSet<Integer>> s1,  double r)
  {
           HashMap<double[], TreeSet<Integer>> temp = new
HashMap<double[], TreeSet<Integer>>();

                                 Iterator itr1 = s1.keySet().iterator();

                                     for(;itr1.hasNext();)
                                     {

                                                               double[] cent1=
(double[]) itr1.next();

                                                               double[]  cent3=
new double[global.size()];
                                                                  for(int
p=0;p<global.size();p++)
                                                                   {
cent3[p]= cent1[p] + r;
                                                                   }

temp.put(cent3,new TreeSet<Integer>());
                                      }
                                     return temp;

  }

  static Map<double[], TreeSet<Integer>> mulr(Map<double[],
TreeSet<Integer>> s1, double r)
  {
             HashMap<double[], TreeSet<Integer>> temp = new
HashMap<double[], TreeSet<Integer>>();
```

```
                                Iterator itr1 = s1.keySet().iterator();


                                   for(;itr1.hasNext();)
                                   {

                                                    double[] cent1=
(double[]) itr1.next();



                                                    double[]  cent3=
new double[global.size()];

                                                      for(int
p=0;p<global.size();p++)
                                                       {

cent3[p]= cent1[p] * r;
                                                       }



temp.put(cent3,new TreeSet<Integer>());
                                    }

                                   return temp;


  }



      static void  female_next_pos()
      {
              double r;


              for(int i=0;i<=nf-1;i++)
               {


                    vibper(i);
                    Map<double[], TreeSet<Integer>> s1 =
sp.get(i);
                    Map<double[], TreeSet<Integer>> s2 =
sp.get(gbest);
                    Map<double[], TreeSet<Integer>> s3 =
sp.get(lbest);
                    r= Math.random();
```

```
                              if( r > pf)
                              {


                               temp1 = mulr(mulr(minus(s3,s1),vib[1]),
beta() );
                               temp2 = mulr(mulr(minus(s2,s1),vib[0]),
beta());
                               temp3=
addr(add(add(s1,temp1),temp2),beta()) ;

                             }

                               else
                               {
                                 temp1 =
mulr(mulr(minus(s3,s1),vib[1]),beta());
                               temp2 =
mulr(mulr(minus(s2,s1),vib[0]),beta());
                                temp3=
addr(minus(minus(s1,temp1),temp2),beta()) ;

                                }


                                  /* r= Math.random();

                                    if(r>pf)
                                    sp.set(i,temp3);
                                    else
                                    centroids(i);*/
                        }



          }


          static void male_next_pos()
          {
                        double[] mweight = new double[n-nf];
                        double median;
                        for(int i=nf,j=0; i<=n-1; i++,j++)
                          mweight[j]=weight[i];


                        Arrays.sort(mweight);

                        if (mweight.length % 2 == 0)
                            median = (  mweight[mweight.length/2] +
mweight[mweight.length/2 - 1]) / 2;
                        else
                           median =  mweight[mweight.length/2];
```

```
for(int i=nf;i<=n-1;i++)
{
    if( weight[i]> median)
    dmalpos.add(i);
}



for(int i=nf; i<=n-1;i++)
{        Map<double[], TreeSet<Integer>> s1 =
sp.get(i);
        if(dmalpos.contains(i))
        {
                vibper(i);


                Map<double[], TreeSet<Integer>>
s2 = sp.get(fbest);

        temp1=     mulr(  mulr(
minus(s2,s1),vib[2]),beta());
            temp2= addr(temp1,0.34);

        temp3= add(temp2,s1);

//sp.set(i,temp3);
        }

        else
         {
             temp1 =  minus( weight_mean_male(),
sp.get(i));
           temp2 =  mulr(temp1, beta());

                temp3=  add(temp2,sp.get(i));

             // sp.set(i,temp3);
        }

        /* double  r= Math.random();

            if(r<pf)
            sp.set(i,temp3);
            else
             centroids(i);*/



}

}
```

```
static Map<double[], TreeSet<Integer>>
weight_mean_male()
{

    double denominator =0.0;

    Map<double[], TreeSet<Integer>>  numerator
= mulr(sp.get(nf),weight[nf]);

    for(int i=nf; i<=n-1;i++)
    {
        denominator += weight[i];
    }

     for(int i=nf+1;i<=n-1;i++)
     {
            numerator
=add(numerator,mulr(sp.get(i),weight[i]));
     }

    return  mulr(numerator,1/denominator);
}


static void mating()
{
        for(int i=nf; i<=n-1;i++)
        {


int worst=index_of_worst();

Map<double[], TreeSet<Integer>>  sworst = sp.get(worst);

find_dfmalpos();

Map<double[], TreeSet<Integer>> snew= find_new(i);

snew=assign_to(snew);

double fnew=  find_fitness(snew);

double  wnew=  find_weight(snew,fnew);

if(wnew < weight[worst])    //1
```

```
sp.set(worst,snew);

                                  }

                           }

                         static int index_of_worst()
                         {

                         double max=0;//2
                         int index=-1;
                          for(int i=0;i<=n-1;i++)
                         {
                             if( weight[i] > max) //3
                             {
                                   max=weight[i]; //4
                                   index=i;
                             }
                        }

                        return index;
                        }

                       static double find_fitness(Map<double[],
TreeSet<Integer>> s1)
                       {
                               double[] sum = new double[k];

                               Iterator itr1 = s1.keySet().iterator();
                               double fitness=0;

                               int  nc=0;
                               for(;itr1.hasNext();)
                              {

                                 double[] cent1= (double[]) itr1.next();
                                sum[nc]=0;

                                TreeSet<Integer> ts=  s1.get(cent1);
```

```
                                Iterator<Integer> itr = ts.iterator();

                                while(itr.hasNext())
                               {
                                   Integer j= itr.next();


                                   double[] cent2  = vecspace.get(j);
                                   double d=0.0;
                                   for(int p=0;p<=global.size()-1;p++)
                                   d += (cent1[p]-cent2[p]) * (cent1[p]-
cent2[p]);

                                   d=Math.sqrt(d);
                                   sum[nc] +=d;
                                }

                                if (ts.size()!=0)
                                sum[nc] /=ts.size();

                          nc++;

                           if(nc>k1-1)
                           break;
                }

             for(int j=0;j<=k1-1;j++)
             fitness +=sum[j];


             return fitness;


    }






                  static double find_weight(Map<double[],
TreeSet<Integer>> s, double f)
                  {

                            double  minfitness=100000;
                              double  maxfitness=0.0;
                            for(int i=0;i<=n-1;i++)
                            {
                                       if (fitness[i] < minfitness)
                                         minfitness=fitness[i];
                                      if (fitness[i] > maxfitness)
                                           maxfitness=fitness[i];
                           }
```

```
                      double  weight=    (  f - minfitness) / (
maxfitness - minfitness);

                      return weight;

                 }

            static   Map<double[], TreeSet<Integer>>
assign_to(Map<double[], TreeSet<Integer>> s)
            {




                               double d=0.0;



                               for(int j=0;j<=cf-1;j++)

                               {
                                             double[] dmincent = new
double[global.size()];

                                             double[] obj=
vecspace.get(j);
                                             Collection c = s.keySet();


                                             Iterator itr = c.iterator();
                                                double  dmin=10000;
                                             ArrayList<double[]>  cc= new
ArrayList<double[]>();

                                               while(itr.hasNext())
                                               {
                                                   double[] cent=
(double[]) itr.next();
                                                     cc.add(cent);
                                                    double sum=0.0;

                                                       for(int
p=0;p<global.size();p++)
                                                          sum += (obj[p] -
cent[p])  * (obj[p] - cent[p]);
                                                     d= Math.sqrt(sum);



                                                    if(d <= dmin)
                                                    {
```

```
                                                dmin=d;

                                                dmincent=cent;
                                        }
                                }

                                if(s.get(dmincent)!=null)
                                {

                                TreeSet<Integer> t=
s.get(dmincent);
                                     t.add(j);
                                    s.put(dmincent,t);
                                }
                                else
                                {
                                       TreeSet<Integer> t=
new TreeSet<Integer>();
                                     t.add(j);
                                    s.put(dmincent,t);
                                }



                }
                        return s;
        }


        static  Map<double[], TreeSet<Integer>>  find_new(int i)
         {

                        double sw=weight[i];
                        for(int j=0;j<=dfmalpos.size()-1;j++)
                        {
                            int index =dfmalpos.get(j);
                          sw +=weight[index];
                        }


                            Map<double[], TreeSet<Integer>> snew =
mulr(sp.get(i),weight[i]/sw);


                         for(int j=0;j<=dfmalpos.size()-1;j++)
                        {
                            snew = add(snew,
mulr(sp.get(dfmalpos.get(j)),weight[dfmalpos.get(j)]/sw));
                        }

                  return snew;
```

```
                }



static void centroids(int i)
{
           Map<double[], TreeSet<Integer>> s=sp.get(i);
            HashMap<double[], TreeSet<Integer>> ns = new
HashMap<double[],TreeSet<Integer>>();
          Collection c = s.keySet();
           double[] ncent =new double[global.size()];
           ArrayList<double[]> acent= new ArrayList<double[]>();
          ArrayList<double[]> ancent= new ArrayList<double[]>();

         Iterator itr = c.iterator();
          while(itr.hasNext())
         {
                    double[] cent= (double[]) itr.next();

                     TreeSet<Integer> ts=  s.get(cent);
                     for(int p=0;p<global.size();p++)
                     {
                         ncent[p]=0;
                     }


                    Iterator<Integer> itr1 = ts.iterator();

                    while(itr1.hasNext())
                    {
                              Integer j= itr1.next();


                              double[] cent1  = vecspace.get(j);

                              for(int p=0;p<global.size();p++)
                              {
                                       ncent[p] +=cent1[p];
                              }
                    }

                   for(int p=0;p<global.size();p++)
                  {
                     ncent[p] /= ts.size();
                  }
                 ns.put(ncent,new TreeSet<Integer>());
        }

      sp.remove(i);
      sp.add(ns);


   }
```

```
    static double beta()
    {
            int a=40,b=100;

             double[] r= new double[141];
             for(int i=0;i<=140;i++)
             {
                 r[i]= Math.random();

              }

             Arrays.sort(r);

             return r[39];
    }






}
```
